# Supplementary figures and images for: Robot-assisted gait training in patients with various neurological diseases: A mixed methods feasibility study
Source: PLoS One. 2024 Aug 27;19(8):e0307434. doi: 10.1371/journal.pone.0307434 (PMC11349200; doi:10.1371/journal.pone.0307434)

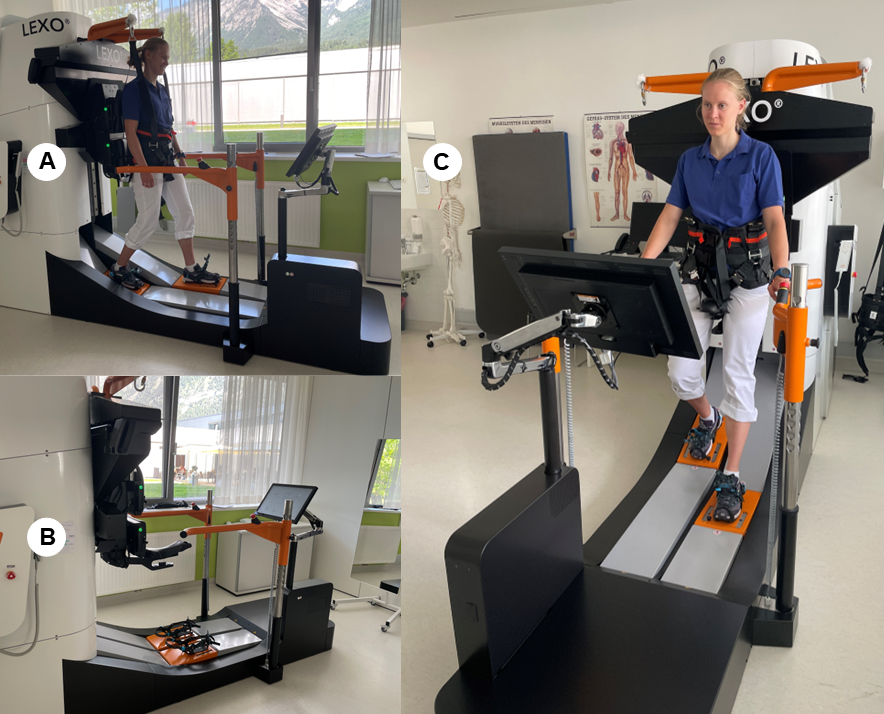

Supplement: S1 Fig — The figure shows the LEXO® gait trainer with harness system (A) and with the saddle (B and C). Reprinted from device training material under a CC BY license, with permission from Tyromotion GmbH, Graz, Austria, original copyright 2023. Written consent for publication was obtained from the person depicted in the pictures. (TIF) [file pone.0307434.s002.tif]

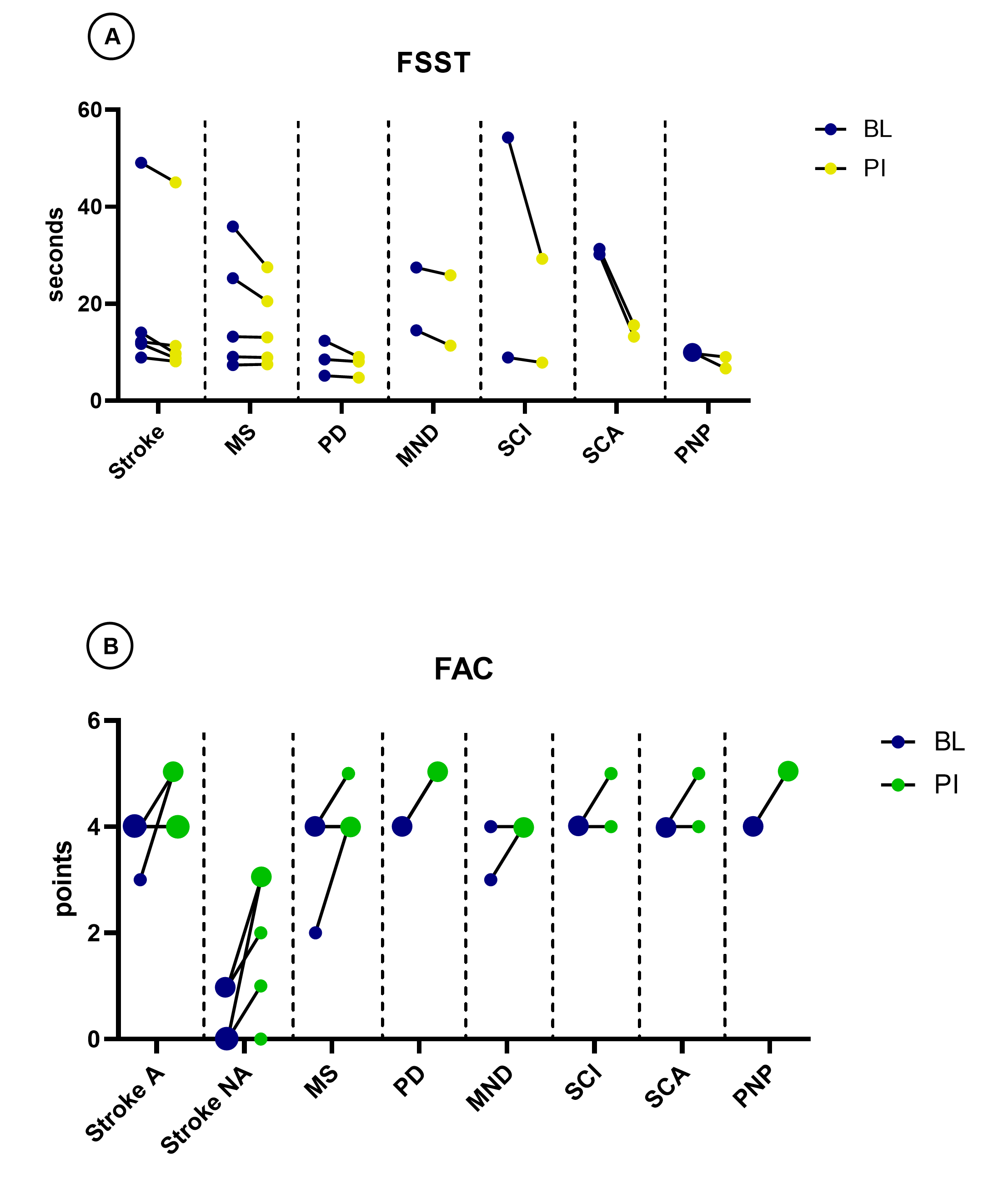

Supplement: S2 Fig — The before-after graphs represent individual patients’ walking and balance performance at baseline (blue dots) and post-intervention (yellow/green dots) on the (A) FSST and (B) FAC. A larger dot size indicates a higher number of patients achieving the same score. With the FAC, an increase in scores represents improvement. With the FSST, a decrease in duration indicates improvement. A, ambulatory patients; BL, baseline; FAC, Functional Ambulation Categories; FSST, Four Square Step Test; MND, motor neuron disease; MS, multiple sclerosis; NA, non-ambulatory patients; PD, Parkinson’s disease; PI, post-intervention; PNP, acute or chronic inflammatory demyelinating polyneuropathy; SCA, spinocerebellar ataxia; SCI, spinal cord injury (spastic para- or tetraplegia). (TIF) [file pone.0307434.s003.tif]
