# Supplementary material for: Robot-assisted gait training in patients with various neurological diseases: A mixed methods feasibility study
Source: PLoS One. 2024 Aug 27;19(8):e0307434. doi: 10.1371/journal.pone.0307434 (PMC11349200; doi:10.1371/journal.pone.0307434)
Supplement: S1 File — (PDF) [file pone.0307434.s004.pdf]

S1 File. Study protocol.

CLINICAL INVESTIGATION PLAN

Robot-assisted gait training in patients with neurological disorders: a comparative multiple case study

Short Title: RAGT-Neur-1

German Title: Geräteunterstütztes Gangtraining bei Patient*innen mit neurologischen Erkrankungen: eine vergleichende multiple Fallstudie

**Clinical investigation Design**	Comparative multiple case study<br>„Other clinical investigation” according to Art. 82 paragraph 1 of the MDR (EU) 2017/745
**Registration**	Once a favourable opinion has been obtained from the ethics committee, an application will be submitted for inclusion in the German Clinical Trials Register (DRKS) or another primary register in accordance with WHO criteria
**Investigational Device:**	LEXO
Version and Date:	1.0 of 04.11.2021
Supersedes Version:	--
Amendment Number:	--

DOCUMENT HISTORY

Version no.	Date	Amendment no.	Author(s)	Amendments/ Reason
Clinical investigation plan, version 1.0	04.11.2021	-	Dr. Barbara Seebacher, MSc	First version

FUNDING

This clinical investigation is a researcher driven study, which is conducted with the financial support of Tyromotion GmbH, Graz, Austria with respect to the provision of a LEXO® for the study site. A Research Agreement has been signed between the Reha Zentrum Münster Betriebs GmbH (RZM) and Tyromotion GmbH.

The agreement states that the purpose of this Collaboration is to systematically collect, analyse, and publish protocols and data on the clinical use of the LEXO® robotic gait trainer in the inpatient setting. A comparative multiple case study will be used to investigate a variety of patients with different main diagnosis.

TYROMOTION primarily aims to collect data on effectiveness (performance) and safety (adverse events, side-effects, and drop-outs) on the LEXO® robotic gait trainer, but also on usability and feasibility, and the quality of the product.

A Purchase Order has been signed between the two parties, where Tyromotion will provide a LEXO® robotic gait trainer for the Reha Zentrum Münster that will conduct the planned clinical investigation followed by a larger randomised controlled trial.

The people involved in decision-marking about this funding have no influence on the planning, conduct and publication of the clinical investigation. None of the investigators or other members of the study team receive a financial compensation for their contributions in this clinical investigation.

Table of content

CONFIDENTIALITY STATEMENT .... FEHLER! TEXTMARKE NICHT DEFINIERT.
DOCUMENT HISTORY **2**
LIST OF ROLES AND RESPONSIBILITIES .... FEHLER! TEXTMARKE NICHT DEFINIERT.
PARTICIPATING INVESTIGATIONAL SITE .... FEHLER! TEXTMARKE NICHT DEFINIERT.
DECLARATION OF THE SPONSOR .... FEHLER! TEXTMARKE NICHT DEFINIERT.
DECLARATION OF THE PRINCIPLE INVESTIGATOR .... FEHLER! TEXTMARKE NICHT DEFINIERT.
DECLARATION OF THE INVESTIGATOR .... FEHLER! TEXTMARKE NICHT DEFINIERT.
DECLARATION OF THE STATISTICIAN .... FEHLER! TEXTMARKE NICHT DEFINIERT.
FUNDING **3**
ABBREVIATIONS **7**
STUDY SYNOPSIS **9**
CLINICAL INVESTIGATION SUMMARY IN LOCAL LANGUAGE **20**
1. INTRODUCTION **22**
1.1. BACKGROUND 22
1.2 RATIONALE 24
1.3 RISK-BENEFIT ANALYSIS 25
2 PURPOSE, OBJECTIVES AND HYPOTHESES OF THE CLINICAL INVESTIGATION **29**
2.1. STUDY PURPOSE 29
2.2. PRIMARY OBJECTIVES 29
2.3. SECONDARY OBJECTIVES 30
2.4. NULL-HYPOTHESIS 31
3 INVESTIGATIONAL MEDICAL DEVICE **31**
3.1. DESCRIPTION 31
3.2. INTENDED PURPOSE 35
3.3. MANUFACTURER 35
3.4. IDENTIFICATION AND TRACEABILITY OF THE INVESTIGATIONAL MEDICAL DEVICE 35
3.5. INTENDED PURPOSE IN THE CLINICAL INVESTIGATION 36
3.6. POPULATION, INDICATIONS AND CONTRAINDICATIONS OF THE INVESTIGATIONAL MEDICAL DEVICE 36
3.7. TRAINING, USAGE AND SAFETY ASPECTS OF USING THE INVESTIGATIONAL MEDICAL DEVICE 40
3.8. DEVICE APPROVAL STATUS 42
4 STUDY DESIGN AND PROCEDURES **42**
4.1. GENERAL ASPECTS, JUSTIFICATION AND STUDY RELATED PROCEDURES 42
4.2 CLINICAL INVESTIGATION SITE 44
4.3 RESPONSIBLE ETHICS COMMITTEE 44
4.4 STUDY REGISTRATION 44
4.5 DATA COLLECTION 44
4.6 SCREENING DATA 45
4.7 DEMOGRAPHIC AND DISEASE-SPECIFIC DATA 45
4.8 PRIMARY AND SECONDARY ENDPOINTS 47
5 PARTICIPANTS **54**
5.1. SAMPLING AND PARTICIPANT RECRUITMENT 54
5.2 RECORDING ADHERENCE TO THE INTERVENTION 54
5.3 SAMPLE SIZE 55
5.4 INCLUSION CRITERIA 55

5.5 EXCLUSION CRITERIA 56
5.6 TIMELINE 58

6 INVESTIGATION PREPARATION PROCEDURES 59

6.1. INVESTIGATOR SELECTION 59
6.2. APPROVAL OF ETHICS COMMITTEE 60
6.3. HANDLING OF AMENDMENTS TO THE CLINICAL INVESTIGATION PLAN 60
6.4. TRAINING REQUIREMENTS 61
6.5. CLINICAL INVESTIGATION MATERIALS AND EQUIPMENT 61
6.6. INVESTIGATIONAL DEVICE / DEVICE ACCOUNTABILITY 62

7. STUDY PROCEDURES 62

7.1. ASSESSMENT AND TREATMENT SCHEDULE 62
7.2. INTERVENTION 63
7.3 SEMI-STRUCTURED OBSERVATION OF THE INTERVENTION 65
7.4 CONCOMITANT TREATMENT 66
7.5 PARTICIPANTS' DROP-OUT 66
7.6 EARLY TERMINATION OR SUSPENSION OF THE CLINICAL INVESTIGATION 67
7.7 MEDICAL CARE AFTER EXIT FROM THE CLINICAL INVESTIGATION 67
7.8 END AND CLOSURE OF THE CLINICAL INVESTIGATION 68

8. STATISTICAL DESIGN AND ANALYSIS 68

8.1. GENERAL ASPECTS OF THE ANALYSIS 68
8.2. PLANNED STATISTICAL ANALYSIS 69
8.3 PLANNED QUALITATIVE DATA ANALYSIS 69
8.1. HANDLING OF MISSING DATA 71
8.2. STRENGTHS AND LIMITATIONS OF THE CLINICAL INVESTIGATION 71

9. SAFETY 74

9.2 ADVERSE EVENT - DEFINITION 74
9.3 ADVERSE REACTION AND SIDE EFFECT - DEFINITIONS 74
9.4 SERIOUS INCIDENT - DEFINITION 74
9.5 SEVERITY 74
9.6 CAUSALITY 75
9.7 ASSESSMENT AND DOCUMENTATION 75
9.8 REPORTING OF SERIOUS INCIDENTS (SAE/SAR) 77

10. QUALITY CONTROL PROCEDURES 77

10.1. DATA COLLECTION METHODS 77
10.2 INVESTIGATOR SITE FILE 77
10.3 STORAGE OF STUDY DOCUMENTS, DATA STORAGE & ERASURE 78
10.4 DOCUMENTATION 78
10.5 DATA MANAGEMENT 79
10.6 DATA QUALITY ASSURANCE MEASURES AND MONITORING 79
10.7 HANDLING QUERIES 80

11. REPORTS 81

11.1. REPORT OF THE STUDY RESULTS 81
11.2. PUBLICATION 81

12. ETHICS AND GOVERNANCE 81

12.1 COMPLIANCE WITH ETHICAL AND REGULATORY REQUIREMENTS 81
12.2 ETHICS APPROVAL 82
12.3 PATIENT INFORMATION AND CONSENT FORM 82
12.4 PATIENT INSURANCE 82
12.5 DATA PROTECTION AND CONFIDENTIALITY 83

12.6 CHANGES WHILE THE CLINICAL INVESTIGATION IS BEING CONDUCTED 84
12.7 INFORMING CLINICAL INVESTIGATION PARTICIPANTS ABOUT THE STUDY RESULTS 85
12.8 DECLARATION OF CONFLICT OF INTEREST 85
12.9 REMUNERATION FOR CLINICAL INVESTIGATION PARTICIPANTS 85

13. REFERENCES 85

ABBREVIATIONS

AD	Autonomic dysreflexia
AIS	American Spinal Injury Association Impairment Scale
ALAP	As low as possible
ALS	Amyotrophic Lateral Sclerosis
ALS-FRS-R	Amyotrophic Lateral Sclerosis Functional Rating Scale, revised version
ASIA	American Spinal Injury Association
BDI-II	Beck Depression Inventory, second edition
CER	Clinical evaluation report
CFO	Chief Financial Officer
CIDP	Chronic inflammatory demyelinating polyneuropathy
CNS	Central nervous system
CP	Cerebral palsy
CRF	Case Report Form
CTO	Chief Technology Officer
DSG	Austrian Data Protection Act
DRKS	Deutsches Register Klinischer Studien, German Clinical Trials Register
EC	Ethics committee
EDSS	Expanded Disability Status Scale
EN ISO 14155	Clinical investigation of medical devices for human subjects – Good clinical practice
EQ-5D-5L	5-level EQ-5D version
FES-I	Falls Efficacy Scale- International Version
GAS	Goal Attainment Scaling
GBS	Guillain Barré Syndrome
GBSDS	Guillain Barré Syndrome Disability Score
GDPR	General Data Protection Regulation
H&Y	Hoehn & Yahr Scale
HRQoL	Health-related quality of life
ICF	Informed Consent Form
ICF	International Classification of Functioning, Disability and Health
ISF	Investigator Site File
MAS	Modified Ashworth Scale
MDSD	Most Different Systems Design
MFRT	Modified Functional Reach Test

MMSE	Mini-Mental State Examination
MSSD	Most Similar Systems Design
MS	Multiple Sclerosis
NIHSS	National Institutes of Health Stroke Scale
PD	Parkinson’s Disease
PI	Principle Investigator
PLS	Primary lateral sclerosis
PMA	Progressive muscular atrophy
PROM	Patient-rated outcome measure
RAGT	Robot-assisted gait training
SARA	Scale for the Assessment and Rating of Ataxia
SD	Standard deviation
SCI	Spinal cord injury
SPIRIT	Standard Protocol Items: Recommendations for Interventional Trials Statement
TA	Thematic Analysis
TCT	Trunk Control Test
TBI	Traumatic brain injury
UPDRS	Unified Parkinson’s Disease Rating Scale
VAS	Visual Analogue Scale
6MWT	6-Minute Walk Test
10MWT	10-Metre Walk Test

STUDY SYNOPSIS

Study title	Robot-assisted gait training in patients with neurological disorders: a comparative multiple case study
Short title	RAGT-Neur-1
DRKS ID	Once a favourable opinion has been obtained from the ethics committee, an application will be submitted for inclusion in the DRKS or another primary register in accordance with WHO criteria
Sponsor	Tyromotion GmbH, Graz, Austria
Principle Investigator	Univ.-Doz. Dr. Christian Brenneis, Reha Zentrum Münster Betriebs GmbH, Münster, Austria
Sites/Countries	Reha Zentrum Münster Betriebs GmbH, Gröben 700, 6232 Münster
Study design	Prospective comparative multiple case study
Objectives	<u>Primary objective:</u><br>The primary objective of this clinical investigation is to explore the feasibility of the methods and of a full-scale randomised controlled trial as assessed by the recruitment rate, retention rate and adherence rate, adverse events as monitored by a structured log, the acceptability of the intervention as evaluated by a Smiley Likert Scale asking four predefined questions, as well as a semi-structured observation during the RAGT intervention for understanding the intervention context and process.<br><u>Secondary Objectives</u><br>Secondary objective 1 of this clinical investigation is to investigate the changes in the fall-rate after a 4-week RAGT as assessed narratively by the number of falls in ambulatory patients.<br>Secondary objective 2 of this clinical investigation is to investigate the changes in walking ability after a 4-week RAGT as assessed by the Functional Ambulation Categories (FAC).<br>Secondary objective 3 of this clinical investigation is to investigate the changes in walking speed after a 4-week RAGT as assessed by the 10-Metre Walk Test (10MWT) in ambulatory patients.

	Secondary objective 4 of this clinical investigation is to investigate the changes in dynamic balance after a 4-week RAGT during walking as assessed by the Functional Gait Assessment (FGA) in ambulatory patients.<br>Secondary objective 5 of this clinical investigation is to investigate the changes in walking distance after a 4-week RAGT as assessed by the 6-Minute Walk Test (6MWT) in ambulatory patients.<br>Secondary objective 6 of this clinical investigation is to investigate the changes in functional mobility after a 4-week RAGT as assessed by the Timed Up and Go test (TUG) in ambulatory patients.<br>Secondary objective 7 of this clinical investigation is to investigate the changes in dynamic balance after a 4-week RAGT as assessed by the Four-Square Step Test (FSST) in ambulatory patients.<br>Secondary objective 8 of this clinical investigation is to investigate the changes in trunk movement after a 4-week RAGT as assessed by the Trunk Control Test (TCT) in non-ambulatory patients.<br>Secondary objective 9 of this clinical investigation is to investigate the changes in the limits of stability with respect to trunk movements after a 4-week RAGT as assessed by the Modified Functional Reach Test (MFRT) in non-ambulatory patients.<br>Secondary objective 10 of this clinical investigation is to investigate the changes in fall-related self-efficacy after a 4-week RAGT as assessed by the Falls Efficacy Scale-International Version (FES-I).<br>Secondary objective 11 of this clinical investigation is to investigate the changes in health-related quality of life (HRQoL) after a 4-week RAGT as assessed by the 5-level EQ-5D version (EQ-5D-5L).<br>Secondary objective 12 of this clinical investigation is to investigate the changes in depression after a 4-week RAGT as assessed by the Beck Depression Inventory, second edition (BDI-II).

	Secondary objective 13 of this clinical investigation is to investigate the changes in fatigue after a 4-week RAGT as assessed by the Fatigue Severity Scale (FSS).<br>Secondary objective 14 of this clinical investigation is to explore the amount of individual and predefined goal achievement after a 4-week RAGT as assessed by Goal Attainment Scaling (GAS).<br>Secondary objective 15 of this clinical investigation is to explore the changes in gait parameters (gait speed, walking distance, number of steps and step length) over the RAGT intervention period as measured by the LEXO® robotic gait trainer.<br>Secondary objective 16 of this clinical investigation is to explore the changes in body weight and gait support (percentage of body weight support, percentage of active/passive gait modes) over the RAGT intervention period as measured by the LEXO® robotic gait trainer.<br>Secondary objective 17 of this clinical investigation is to develop a theory on the causal relationships between the RAGT and study outcomes with respect to the different study patient populations. This objective does not involve patient assessments.
Endpoints	Primary endpoint:<br>The primary endpoint of this clinical investigation is the feasibility of the methods and of a full-scale randomised controlled trial. This will be assessed by the recruitment, retention and adherence rates, a structured adverse events log, a Smiley Likert Scale asking four predefined questions on the acceptance of the intervention, as well as a semi-structured observation during the RAGT intervention for understanding the intervention context and process.<br>Secondary endpoints and assessments:<br>1.1 Number of falls– falls log [ambulatory patients]<br>1.2 Walking ability - Functional Ambulation Categories (FAC) [ambulatory patients]<br>1.3 Walking speed - 10-Metre Walk Test (10MWT) [ambulatory patients; assessed in clinical routine]<br>1.4 Dynamic balance during walking - Functional Gait Assessment (FGA) [ambulatory patients]

	1.5 Walking distance - 6-Minute Walk Test (6MWT) [ambulatory patients; assessed in clinical routine]<br>1.6 Functional mobility - Timed Up and Go test (TUG) [ambulatory patients]<br>1.7 Dynamic balance - Four-Square Step Test (FSST) [ambulatory patients]<br>1.8 Trunk movement - Trunk Control Test (TCT) [non-ambulatory patients]<br>1.9 Limits of stability with respect to trunk movement - Modified Functional Reach Test (MFRT) [non-ambulatory patients]<br>1.10 Fall-related self-confidence - Falls Efficacy Scale-International Version (FES-I)<br>1.11 Health-related quality of life - 5-level EQ-5D version (EQ-5D-5L)<br>1.12 Depression - Beck Depression Inventory, second edition (BDI-II)<br>1.13 Fatigue - Fatigue Severity Scale (FSS)<br>1.14 Goal achievement - Goal Attainment Scaling (GAS)<br>1.15 Gait parameters (gait speed, walking distance, number of steps and step length) – measured by LEXO® during the intervention as part of the clinical routine<br>1.16 Body weight and gait support (percentage of body weight support, percentage of active/passive gait modes) - measured by LEXO® during the intervention as part of the clinical routine
Study population	<u>Inclusion criteria</u><br>Potential study participants must meet all listed inclusion criteria in order to be eligible for participating in this clinical investigation. Patients will be recruited into this clinical investigation if they have been diagnosed with a first-ever ischemic or hemorrhagic stroke, multiple sclerosis (MS), Parkinson’s Disease (PD), a spastic para- or tetraplegia caused by any type of spinal cord lesion, mild to medium grade hereditary ataxia, acute or chronic inflammatory demyelinating polyneuropathy (e.g., GBS, CIDP) or a motoneuron disease (e.g., ALS). All patients must be in a clinically stable phase of their disease. Diagnoses will be confirmed using established criteria as outlined in Chapter

	such (1) Stroke is diagnosed based on current criteria according to the Guideline of the German Society of Neurology (2017) (Hennerici & Kern, 2017) (2) MS is diagnosed according to the revised McDonald criteria valid at the time of diagnosis (Polman et al., 2011; Thompson et al., 2018). (3) (Idiopathic) PD is diagnosed according to the UK Brain Bank criteria (Hughes et al., 1992). (4) SCI is diagnosed based on current criteria according to the Guideline of the German Society of Neurology (DGN 2012) or current NICE Guideline (Spinal injury: assessment and initial management. Spinal injury assessment: assessment and imaging for spinal injury. NICE Guideline NG41, 2016) valid at the time of diagnosis.(5) Hereditary ataxia is diagnosed according to the Ataxia Medical Guidelines 2016 or earlier versions, developed by Ataxia UK, London (de Silva et al., 2019). (6) For GBS, the current diagnostic criteria are used (Willison et al., 2016). Chronic inflammatory demyelinating polyneuropathy (CIDP) is diagnosed according to diagnostic criteria of the European Federation of Neurological Societies/Peripheral Nerve Society Guideline on the management of chronic inflammatory demyelinating polyradiculoneuropathy- First Revision ("European Federation of Neurological Societies/Peripheral Nerve Society Guideline on management of chronic inflammatory demyelinating polyradiculoneuropathy: report of a joint task force of the European Federation of Neurological Societies and the Peripheral Nerve Society--First Revision," 2010) or earlier versions.(7) Motoneuron disease is diagnosed using the diagnostic criteria for ALS from the World Federation of Neurology as the El Escorial Criteria (Brooks, 1994), in their revised version as the Airlie House criteria (or El Escorial revised) (Brooks et al., 2000) and the Awaji criteria (de Carvalho et al., 2008). There are no formal criteria for progressive muscular atrophy (PMA) and primary lateral sclerosis (PLS). Recently, the Gold Coast criteria were developed and are proposed (Johnsen, 2020).<br>Patients with subacute, severe stroke who are non-ambulatory are eligible if they can be verticalised for at least 1 hour twice a day (which is a requirement for inpatient rehabilitation at the Reha Zentrum Münster). Patients with motor neuron disease in the early, active stage of the disease

	will be included and patients with mild to moderate hereditary ataxia. Patients will only be included if they are able to participate in this clinical investigation over its complete duration, which will be evaluated by the study PI. Further inclusion criteria will be impairment in walking according to the neurological exam assessed by a neurologist and Functional Ambulation Categories (FAC) of 0-4, intact cognitive function as defined by a score of ≥24/30 points on the Mini Mental State Examination (MMSE) (Folstein et al., 1975).<br><br>Adult patients aged between 18 and 99 of female, male and diverse genders, of any ethnicity and German speaking verbally and written language will be included.<br><br><u>Exclusion criteria</u><br><br>Patients will be excluded from this clinical investigation if they meet any of the criteria listed as follows.<br><br>• Concomitant disease (such as malignant disease, other severe neurological, orthopaedic or psychiatric diseases, cardiac contraindications)<br>• Acute, pronounced pain symptoms despite conventional pain therapy<br>• joint contractures, joint arthrodesis or severe spasticity (stiff/immobile joint) in the area of the lower extremity, extremely disproportionate growth of the legs and/or spinal column<br>• Body weight of less than 15 kg or more than 180 kg<br>• Body height of less than 100 cm or more than 200 cm<br>• Insufficient compliance e.g., patients with a serious mental illness or severe neurosis<br>• Significant reduction in bone density (osteopenia or osteoporosis), increased risk of bone fracture<br>• Osseous or joint instability (non-consolidated fractures, osteogenesis imperfecta, unstable spinal column, pseudoarthrosis, hip, knee or ankle joint instability)<br>• Cardiac contraindications<br>• Severe vascular diseases of the lower limbs<br>• High-grade ataxia

	• Skin lesions in areas which get in contact with the device or the harness system (decubitus); infections, skin ulcers, late effects of previous injuries, especially of the lower extremity<br>• States of health preventing active rehabilitation (e.g., respiratory diseases, orthopaedic diseases, cognitive impairments restricting communication, aphasia, neuropsychological disorders, infections or inflammatory diseases, osteomyelitis)<br>• Severe apraxia<br>• Severe osteoarthritis in the area of the joints of the lower extremity<br>• Risk of autonomic dysreflexia (AD) in patients with spinal cord injury (SCI) with neurological levels of TH6 or cranial<br>• Reduced compliance<br>• Uncooperative or (self)aggressive behavior<br>• Cardiac diseases, e.g., heart failure and thoracotomy, uncontrolled orthostatic hypotension or other circulatory problems, circulatory disorders of the lower limbs<br>• Recent joint injury or endoprosthetics (e.g., total endoprosthetics, knee or hip joint replacement, reconstruction of the cruciate ligament or meniscus) with contraindicated ranges of movement or load limits<br>• Consolidated fractures in the area of the lower extremity within a period of 6 months after injury<br>• Insufficiently treated epilepsy<br>• Recently occurred seizures or increased risk of seizures<br>• Mechanical ventilation<br>• Lack of head control<br>• Long-term infusions (e.g., Baclofen pump, intrathecal pumps, PEG tube …) or<br>• stimulators (e.g., pacemakers, nerve stimulators)<br>• Enterostomy/ostomy<br>• Sensory impairments of the lower extremities and the torso, especially reduced sensation of pain<br>• Pregnancy as assessed by a pregnancy test in women of childbearing age before enrolment into the study<br>• An MS relapse or other disease exacerbation for any of the included diseases during the intervention period will lead to the exclusion of a participant from the study

	• Any changes or adjustments to medication that has an influence on the participant's walking ability/performance will lead to the exclusion of the patient.
Indication and sample size	Total sample size N=28<br>5 patients after severe stroke in subacute stage; 5 patients after mild to moderate stroke in chronic stage; 5 patients with multiple sclerosis; 5 patients with Parkinson's disease; 2 patients with spastic para- or tetraplegia; 2 patients with hereditary ataxia; 2 patients with acute or chronic inflammatory demyelinating polyneuropathy and 2 patients with motor neuron disease.
Intervention	Robot-assisted gait training (RAGT) using the LEXO® robotic gait trainer (Tyromotion, Graz, Austria) and supervised by the responsible physiotherapists will be performed as part of the clinical rehabilitation routine 45 minutes, 4x/week, for 4 weeks. The RAGT will be integrated into patients' inpatient rehabilitation and be planned at similar daytimes to reduce patient burden and facilitate comparability. A standard RAGT protocol will be used and adapted to the patients, with regard to the different neurological disease groups and related symptoms. Second, the training will be adapted according to the individual performance level of the participants. During the 45 min training period, participants will be allowed to take a break at any time, if this is desired or necessary. Based on the findings of the previous literature and with advances in walking performance, the training parameters for the different patient groups have been selected and will be progressed in difficulty (e.g., step length, gait speed, body weight support). Explicit feedback on the walking performance will be given by the supervising therapist and implicit feedback will be provided as augmented performance feedback via a computer screen in front of the patient, based on the different measurements of parameters by the robot. For example, visual implicit feedback will be given on distance, duration, and number and length of steps.
Study procedures	After eligibility screening with respect to the inclusion and exclusion criteria of this clinical investigation, patients will be informed about the study by the study PI or investigator and asked for their interest in participating in this clinical

	investigation. If they express their interest in participating, they will be screened by a psychologist for cognitive impairment and for walking impairment using the Functional Ambulation Categories in addition to a neurological exam. If patients pass the screening (if Mini Mental State Examination ≥24/30 points, walking impairment, FAC 0-4/5 points)), written informed consent will be obtained from them, followed by a Baseline examination. Then, the clinical investigation intervention will be conducted for 4 weeks, and a semi-structured observation of the intervention at weeks 1 and 4. After the post-intervention examination, the clinical investigation will be terminated.
Statistical Analysis	IBM SPSS Software, release 27.0 (IBM Corporation, Armonk, NY, USA) and GraphPad Prism 9, San Diego, California, will be used for the data analyses. Statistical significance will be defined as a two-tailed p-value <0.05. The percentage of missing data will be recorded. Using Little's test of missing completely at random (MCAR) (Little, 1988) the data will be checked, signified by a p-value >0.05.<br>Descriptive statistics will be used for the baseline demographic variables, primary and secondary endpoints. Continuous data will be checked for normal distribution using the Shapiro Wilk Test, Q-Q plots and histograms. Raw count (absolute and relative frequencies, N (%) will be presented for count data (N females and males, N falls, most affected body part, and N adverse events if any, adherence (N of training sessions), recruitment, retention and adherence rates) and nominal data (gender)). Medians (range; 25th and 75th percentiles) will be reported for ordinal data (MMSE, NIHSS, EDSS, UPDRS, H&Y, ASIA Scale, SARA, MAS, ONLS, ALSFRS-R, FAC, TCT, FES-I, EQ-5D-5L, BDI-II, FSS, GAS, Smiley Likert Scale), mean (standard deviation (SD)) will be reported for continuous data (age, years of education, disease duration, 10MWT, FGA, 6MWT, TUG, FSST and MFRT).<br>With respect to the primary endpoint of feasibility, the recruitment rate (%) will be estimated by dividing the number of consenting patients by the N of eligible patients, multiplied by 100. The retention rate determined by dividing the N of patients who completed the clinical investigation by the N of the total sample, times 100. Adherence rates will be

	calculated by dividing the N of RAGT sessions (16x) performed by the patients, divided by the N of the scheduled RAGT training sessions over the 4 week study period, times 100 (Osterberg & Blaschke, 2005). The eligibility, recruitment and adherence rates will be calculated with their 95% CI according to the Wilson 'score' method cited by Newcombe (Newcombe, 1998); when the proportion is close to 0 or 1, a Poisson approximation as described by Brown and colleagues will be used (Brown et al., 2001). Furthermore, the number of (serious) adverse events and side effects will be reported.<br>The first phase of the analysis will involve a descriptive analysis for all disease groups using the mean (SD) or median (range, 25th and 75th percentiles). The second phase of the analysis will include inferential statistics using a pre-post analysis of the data from the total sample (one group). For continuous data, a paired t-test will be performed and for ordinal data, a Wilcoxon Signed Rank test.
Timeline of the clinical investigation	Study-specific:<br>Recruitment duration: 15 months<br>1.2.2022 Planned start: First Patient First Visit (FPFV)<br>31.3.2023, 2022 Last Patient First Visit (LPFV)/Last Subject In (LSI)<br>30.4.2023 Planned end: Last Patient Last Visit (LPLV)/Last Subject Out (LSO)<br><br>Patient-specific:<br>Active intervention duration: 4 weeks (baseline assessment, 4-week intervention, post-intervention assessment)
Funding	This clinical investigation is a researcher driven study, which is conducted with the financial support of Tyromotion GmbH, Graz, Austria with respect to the and provision of a LEXO® for the study site. A Research Agreement has been signed between the Reha Zentrum Münster Betriebs GmbH (RZM) and Tyromotion GmbH.<br>The agreement states that the purpose of this Collaboration is to systematically collect, analyse, and publish protocols and data on the clinical use of the LEXO® robotic gait trainer in the inpatient setting. A prospective comparative multiple case study will be used to investigate a variety of patients with different main diagnosis.

	TYROMOTION primarily aims to collect data on effectiveness (performance) and safety (adverse events, side-effects, and drop-outs) on the LEXO® robotic gait trainer, but also on usability and feasibility, and the quality of the product.<br>A Purchase Order has been signed between the two parties, where Tyromotion will provide a LEXO® robotic gait trainer for the Reha Zentrum Münster that will conduct the planned clinical investigation followed by a larger randomised controlled trial.<br>The people involved in decision-marking about this funding have no influence on the planning, conduct and publication of the clinical investigation. None of the investigators or other members of the study team receive a financial compensation for their contributions in this clinical investigation.
Compliance Statement	This clinical investigation will be conducted in compliance with the clinical investigation plan, the current version of the declaration of Helsinki, the EN ISO 14155 as well as all national and local legal and regulatory requirements.

CLINICAL INVESTIGATION SUMMARY IN LOCAL LANGUAGE

Hintergrund: Gangstörungen stellen ein relevantes Symptom bei Patient*innen mit neurologischen Erkrankungen dar. Bei Multipler Sklerose (MS) können beispielsweise Symptome wie Schwäche und Spastik, Verlust der Propriozeption und Koordination, vestibuläre, kognitive und visuelle Dysfunktion, Fatigue und Schmerzen zu einer Gangbeeinträchtigung beitragen. Beim Idiopathischen Parkinsonsyndrom (PD) wird die Mobilität durch Rigor, Hypo- und Bradykinesie, Flexionshaltung und Haltungsinstabilität beeinträchtigt. Nach einem Schlaganfall ist eine hemiparetische, spastische Gehbehinderung ein häufiges Symptom bei ca. 75 % der Patient*innen. Hereditäre Ataxien sind primär durch progrediente Koordinationsdefizite von Bewegung und Sprechen sowie einem breitbasigen, unkoordinierten, unsicheren Gang charakterisiert. Bei Patient*innen mit spastischer Para- oder Tetraplegie, akuter oder chronischer entzündlicher demyelinisierender Polyneuropathie und Motoneuronerkrankung ist eine Gangstörung ebenso ein wesentlicher Faktor ihrer Behinderung, die zu Einschränkungen in ihrem Alltag führt. Zahlreiche Studien stellten fest, dass eine Gehbehinderung zu Depressionen und Angstzuständen beiträgt, die soziale Teilhabe und die Lebensqualität stark beeinträchtigt. Darüber hinaus wiesen einschlägige Studien auf den Zusammenhang zwischen Gehbehinderung und Erwerbstätigkeit bei Patient*innen mit neurologischen Erkrankungen hin. Patient*innen mit neurologischen Erkrankungen können aufgrund ihrer Gangstörung und Beeinträchtigung bei Aktivitäten des täglichen Lebens ihrer Arbeit nicht mehr nachgehen und sind bezüglich sozialer Aktivitäten beeinträchtigt. Neurologische Defizite wie Gang-, Gleichgewichts- und Koordinationsstörungen können durch intensives und repetitives aufgabenorientiertes Training reduziert werden. Motorisches Lernen, basierend auf dem Prozess der neuronalen Plastizität, kann bei Patient*innen mit leichter bis schwerer Behinderung durch Neurorehabilitationsstrategien gefördert werden. Prinzipien einer effektiven Neurorehabilitation sind bereits bekannt und werden in der stationären und ambulanten Neurorehabilitation routinemäßig angewandt. In der stationären Rehabilitation dient etwa ein geräteunterstütztes Gangtraining (robot-assisted gait training, RAGT) dazu, um die Prinzipien des massierten (konzentrierten) Trainings, hoher Dosis, aufgaben- und zielorientierten Trainings unter progressiver Anpassung der Schwierigkeit und implizitem sowie explizitem Feedback zur Verbesserung des Ganges zu nützen.

Notwendigkeit für die Durchführung dieser Klinische Prüfung: Unseres Wissens untersuchte bisher keine Studie in der klinischen Routine eingesetztes RAGT bei verschiedenen Patient*innengruppen mit neurologischen Erkrankungen in einem stationären Rehabilitationssetting. Der Zweck dieser Klinische Prüfung ist daher, in einer vergleichenden multiplen Fallstudie die Machbarkeit und Akzeptanz sowie die präliminären Effekte von RAGT bei Patient*innen nach Schlaganfall, mit MS, PD, spastischer Para- oder Tetraplegie, hereditärer Ataxie, akuter / chronischer entzündlicher demyelinisierender Polyneuropathie und Motoneuronerkrankung zu untersuchen.

Methoden: Die vergleichende multiple Fallstudie wird am Reha Zentrum Münster, Tirol durchgeführt. 28 Patient*innen nach schwerem Schlaganfall in der subakuten Phase (n=5) und nach mild bis moderatem Schlaganfall in der chronischen Phase (n=5), mit MS (n=5), PD (n=5), spastischer Para- oder Tetraplegie (n=2), hereditärer Ataxie (n=2), akuter / chronischer entzündlicher demyelinisierender Polyneuropathie (n=2) und Motoneuronerkrankung (n=2) im Alter von 18-99 Jahren werden anhand definierter Ein- und Ausschlusskriterien nach einem Screening für kognitive Beeinträchtigung und Gangstörung und schriftlicher informierter Einwilligung in diese Klinische Prüfung eingeschlossen. Die Intervention ist in der klinischen Routine durchgeführtes RAGT: 45 min, 4x/Woche, 4 Wochen lang. Zur Baseline- und Postinterventionstestung werden Gang- und Gleichgewichtstests, Fragebögen und eine Zielevaluierung durchgeführt. Die Analyse beinhaltet eine deskriptive Statistik innerhalb der jeweiligen Krankheitsgruppen und eine Inferenzstatistik mit prä-post Analysen der Gesamtstichprobe.

1. INTRODUCTION

1.1. Background

Walking impairment is a relevant symptom in patients with neurological diseases. For example, in multiple sclerosis (MS), symptoms such as weakness and spasticity, loss of proprioception and coordination, vestibular, cognitive and visual dysfunction, fatigue and pain can all contribute (Pearson et al., 2004). With Parkinson’s disease, mobility is determined by rigidity, hypo- and bradykinesia, flexion posture and postural instability, and other symptoms such as REM sleep disorder associated fatigue can have important effects (Cheng et al., 2012). After a stroke, walking impairment is a disabling and frequent symptom (Rose et al., 2017). Approximately 75% of stroke survivors live with some form of gait disorder (Sullivan et al., 2009). Symptoms of the hereditary ataxias are progressive coordination deficits of movement and speech, and a wide-based, uncoordinated, unsteady gait (Bird, 1993). Other clinical symptoms include spasticity, neuropathy and cognitive or behavioral deficits (Bird, 1993). In patients with spastic para-or tetraplegia, acute or chronic inflammatory demyelinating polyneuropathy and motoneuron disease, walking impairment is also a main contributor to their disability, which leads to restrictions in their everyday lives. Numerous studies have shown that walking disability contributes to depression and anxiety, heavily impacts social participation and quality of life (Ochoa-Morales et al., 2019; Pearson et al., 2004). Furthermore, relevant studies have pointed out the relationship between walking disability and employment in patients with neurological disorders (Jarvis et al., 2019; Raggi et al., 2016). People who have had a stroke or MS are unable to work or participate in social activities because of their walking difficulties and impairments in activities of daily living (Jarvis et al., 2019).

Patients post stroke are usually treated intensively in the acute and subacute phases to enhance motor performance. This is because the recovery of walking and further sensorimotor and cognitive functions mainly takes place within the first 11 weeks after a stroke. A principle here is: the milder the paralysis, the faster the recovery (Jørgensen et al., 1995). As for patients with more severe disability, *the* principle of neurorehabilitation is ‘use it or lose it’ [function] (Shors et al., 2012). In patients with amyotrophic lateral sclerosis (ALS), training is more intensive in the first, so called active phase after diagnosis, when the physical condition is better, in order to maintain the walking and other motor functions for as long as possible (Calabrò et al., 2019). In patients with a progressive disease such as MS, Parkinson’s, ALS or hereditary ataxias, a main reason for undergoing neurorehabilitation is to preserve their ability to walk (Cheng et al., 2012; Wirz et al., 2001). Neurological deficits, such as walking impairment, poor coordination and balance loss, can be reduced by intensive and repetitive task-oriented training (Nilsson et al., 2001; Van Peppen et al., 2004).

Motor learning is present in patients with mild to severe disability, based on the process of neural plasticity, and can be boosted by a range of neurorehabilitation strategies (Maier et al., 2019). The principles underlying effective neurorehabilitation are well-known yet, and these are applied in inpatient and outpatient neurorehabilitation on a routine basis. A relevant review has identified the most salient principles of motor learning based on the evidence so far. These comprise massed practice, dosage, task-specific practice, goal-oriented practice, variable practice, increasing difficulty, explicit feedback in terms of knowledge of results and implicit feedback in terms of knowledge of performance (Maier et al., 2019).

The first principle, massed practice, has been defined as work episodes with very brief to no rest periods (Schmidt & Lee, 2011). In these episodes, repetitive training for a skill can be trained in a constant or blocked way (Mulligan et al., 1980). For the field of rehabilitation, this means prolonged and repeated use of the affected limb (Taub et al., 1999), which is clearly achieved for the lower limbs with a robotic gait trainer, and sufficient time for rest is ensured at the same time. Dosage generally operationalises the number of hours of therapy (Kwakkel, 2006; Veerbeek et al., 2014), the duration and frequency of sessions (Dobkin, 2005) or the stimulation of learning for the amount of training required (Wadden et al., 2017). The number, duration and frequency of the therapy sessions are specified by the clinical investigation plan. The third principle of motor learning is task-oriented training. Task-specific training assumes that changes in the condition of a task can cause a change in the required skills (Schmidt & Lee, 2011). Task-specific training i.e., training of a task such as walking facilitates motor learning and retention (Boyd et al., 2010). Fourth, goal-oriented practice indicates that when attention is focused on the effect of the movement rather than the movement itself, motor performance and learning is improved (Wulf & Prinz, 2001) Task-specific exercise and goal-oriented exercise are addressed by different factors in robot-assisted gait training (RAGT): through the targeted training of walking and the possibility of an additional virtual environment.

The fifth principle of motor learning is the progression in difficulty. This shows that training that is adapted to the learner's abilities leads to better learning outcomes than when the level of difficulty is fixed (Wickens et al., 2013). The difficulty of the RAGT can be continuously adapted by varying the difficulty of the training from passive to supportive to active and through other parameters for example body weight support and step length. Further two principles of motor learning are explicit and implicit feedback. Explicit feedback has been defined as verbal, terminal and reinforced feedback on goal attainment (Salmoni et al., 1984). Here, qualitative or quantitative feedback is given regarding a task outcome e.g., correctness and accuracy (Mazzoni & Krakauer, 2006; Subramanian et al., 2010). Implicit feedback is defined as feedback on movement performance in the form of verbal description, replays of recordings or demonstrations (Gentile, 1972). In RAGT, explicit feedback can be given by the supervising therapist and implicit feedback can be provided as

augmented performance feedback via a computer screen in front of the patient, based on the different measurements of parameters by the robot. For example, visual implicit feedback can be given on distance, duration, and number and length of steps.

In inpatient neurorehabilitation, the principles of motor learning are applied to facilitate the rehabilitation outcomes in patients e.g., with the help of robot-assisted therapy. A variety of studies has shown that RAGT is based on the theories and principles of motor learning (Bruni et al., 2018; Calabrò et al., 2018; Portaro et al., 2017). RAGT can be categorised into gait training using different systems, the stationary system and the overground system (Calabrò et al., 2016). Stationary devices are realised by a movable floor platform in a fixed structure and can be divided into treadmill gait trainers (such as the Lokomat® exoskeleton [Hocoma AG, Volketswil, Switzerland]) and programmable foot end effector trainers (e.g., the GE-O-System® [Reha Technology AG, Olten, Switzerland] and LEXO® [Tyromotion, Graz, Austria]) depending on the type of mobile platforms used (Bruni et al., 2018; Calabrò et al., 2016). The Reha Zentrum Münster uses a Lokomat® exoskeleton and a LEXO® foot end-effector trainer for RAGT in patients with neurological disabilities. The end effector system promotes the patient's own activity and enables active and more physiological gait training, due to adaptability of the pelvis support.

1.2 Rationale

To the best of our knowledge so far, no study has evaluated routinely used RAGT in different groups of patients with neurological diseases in an inpatient rehabilitation setting. No comparative study has ever investigated RAGT in patients with stroke, multiple sclerosis, Parkinson’s Disease, spastic para- or tetraplegia caused by any type of spinal cord lesion, hereditary ataxia, acute or chronic inflammatory demyelinating polyneuropathy and motoneuron disease. These neurological diseases are associated with different symptom complexes, involving distinct central and peripheral nervous system sensorimotor areas and pathways, which is why patients may interact differently with a robotic gait trainer. In addition, no study has ever combined a quantitative and qualitative data collection approach using subjective and objective methods in these populations. Therefore, a multiple case-oriented research strategy is necessary to acquire knowledge about the outcomes from RAGT within the different patient (disease) groups, learn about similarities and differences between individual cases and neurological disorders within the study intervention period and about emerging patterns (Ababacar et al., 2020; Nordbeck, 2013). A synthesis across cases seems useful that extends beyond the comparison of similarities and differences to generate information on the reasons and explanations as to the findings from the RAGT intervention. Thus, a prospective comparative multiple case study is necessary for the development of theory, to inform the hypotheses of a follow-up randomised controlled trial (Goodrick, 2014).

1.3 Risk-benefit analysis

The performance of the RAGT intervention and walking/balance tests may trigger adverse events and side effects. These may include increased and reversible fatigue and mild muscle pain associated with the gait training and will be minimised by using predefined and individualised strategies for progression of difficulty and breaks if needed, as outlined in detail in Chapter 7.2. The risk of this is therefore classified as low. RAGT is performed as part of the clinical routine in inpatient rehabilitation settings using different devices, with the LEXO® robotic gait trainer being a CE marked medical device that has undergone a comprehensive risk assessment (please see below for details). Any contraindications for the RAGT and any additional exclusion criteria related to this clinical investigation will be complied with. In addition, continuous monitoring for adverse events will be performed using a structured adverse events log. In addition, the clinical investigation site is an inpatient rehabilitation centre with a large medical team that is available on a 0-24/7 basis. At weeks 1 and 4, a semi-structured observation of the intervention will take part as a qualitative study measure, and any inconveniences of the participants will be noted and taken care of. So far, based on a systematic search within the worldwide most important databases for medical devices, no results and no adverse events were reported for the LEXO and its operating software tyroS. Furthermore, no adverse events happened with LEXO® from its market introduction. With respect to the walking, mobility and dynamic balance assessments, some of them are also performed as part of the clinical routine at the clinical investigation site and at other Austrian rehabilitation centres, with the remaining being carefully selected, standardised, validated assessments with excellent psychometric properties in patients with neurological diseases. As for the walking tests, it is assumed that falls may occur in rare cases, but these may also occur outside of the physiotherapy and study assessment setting in the daily lives of patients with neurological disabilities. To keep the risk of falls as low as possible, the walking assessment will be performed along a wall and under the supervision of a trained physiotherapist. Physical contact should not take place in order not to impact test performance but will be used to avoid a fall.

The RAGT will be integrated into patients' inpatient rehabilitation and be planned at similar daytimes to reduce patient burden and facilitate comparability. The baseline and post-intervention examinations involve walking and balance tests and self-report questionnaires. This means that the patients may alternate between physical and mental activities, will be supported as required and as permissible with respect to the test instructions, and they may take a rest at any time. Therefore, the clinical investigation procedures are classified as low burdensome. Since the clinical investigation will be conducted in an inpatient rehabilitation environment, the participants will not be required to travel to the clinical investigation site.

With respect to the benefits from the RAGT, it is expected that the patients will improve their walking or gain walking ability (severely affected patients post stroke in the subacute phase). The results gained from this clinical investigation will help to optimise RAGT

protocols with respect to patients with different neurological diseases, which will contribute to better rehabilitation outcomes in the long-term. In summary, the expected benefits – treatment optimisation and more efficient and effective rehabilitation – outweigh the low-level risks and burden for patients, therefore the study procedures only represent a minor health risk and merely slight burden for patients, based on the current state of medical knowledge.

Risk Assessment

The prevention of risks was carried out according to the criteria that are specified by the International Organization for Standardization (ISO) EN ISO 14971 risk management, as well as by the application of the medical device directive 93/42/EEC concerning medical devices. After risk mitigation strategies for the LEXO® robotic gait trainer have been employed by the manufacturer, risks were rated as “ACCEPTABLE”.

The overall medical benefit outweighs the risk of specific endangerments, the possible combinations of all known endangerments, as well as the total residual risk device status

Demonstration of an acceptable risk-benefit-ratio

Demonstration that known and expected risks for the medical product were reduced to a minimum, and that these risks are acceptable when weighed against the benefits for the patient, when compared to current treatment options

- if there are risks arising of the use of the LEXO® system
- if the benefit/risk profile, undesirable side-effects (whether previously known or newly emerged) and risk mitigation measures are still
    - compatible with a high level of protection of health and safety and acceptable according to current knowledge/ the state of the art
    - correctly addressed by the manufacturer's current post-market surveillance plan
- if there are any specific clinical concerns that have newly emerged and need to be addressed

The risk management file identified risks arising from therapy using of the LEXO® robotic gait trainer. All identified risks were rated for their probability of occurrence (frequent, probable, occasional, remote, improbable) and for the severity of consequences (negligible, marginal, critical, catastrophic) before and after corrective actions.
The prevention of risks was carried out according to the criteria that are specified by the international standard EN ISO 14971 risk management, as well as by the application of the medical device directive 93/42/EEC concerning medical devices. After risk mitigation

strategies for the LEXO® robotic gait trainer have been employed by the manufacturer, risks were rated as “ACCEPTABLE”.
The overall medical benefit outweighs the risk of specific endangerments, the possible combinations of all known endangerments, as well as the total residual risk.

Claims on clinical safety

The LEXO® system
1. has been CE marked as a medical product class lla in Europe according to directive 93/42/EWG
2. has passed the 60601-1 test, the machinery directive 2006/42/EG, and the 60601-1-2 test
3. is developed, approved, and produced according to the following standards: ISO 13485, IEC 62366, ISO 14971,
ISO 10993, RoHS/Reach, and IEC 62304
4. has biocompatibility according to ISO 10993

The following is a summary of the risk analysis for the Lexo with the Intended Use and Intended Population described above (CE mark). Risks have been analysed and evaluated during Design and Development, Manufacturing and Feedback from customers.

The risks from all identified hazards were assessed.

Evaluation of the overall residual risk:

ACCEPTABLE	X	ALAP		UNACCEPTABLE	

Risk-Benefit Analysis:

Med. Benefit outweighs risk of the individual hazard examined	Yes
Med. Benefit outweighs possible combinations of all hazards considered	Yes
Med. Benefit outweighs total residual risk	Yes

if no: risk is not acceptable - no placing on the market possible

The overall risk is classified by management as ACCEPTABLE (no risk or no risk that can be further minimized).

LEXO® has been tested and CE marked for the intended use and the intended population mentioned in the user manual.

Claims on clinical safety: LEXO®

- *Has passed the 60601-1 test, the machinery directive 2006/42/EG and the 60601-1-2 test*
- *Is developed, approved and produced according to the following ISO standards: ISO 13485, IEC 62366; ISO 14971; ISO 10993 and IEC 62304*

An adverse event data base report has been made based on the systematic search within the worldwide most important databases for medical devices. The results cover adverse event reports, product recalls, and complaints for all years available up to present. Identified search terms targeted Tyromotion devices and software as well as equivalent devices named in the clinical evaluation report (CER). No results and no adverse events were reported for any Tyromotion device and its operating software tyroS version 6.1.

No adverse events happened with LEXO® from its market introduction.

Expected benefit

The LEXO® device is intended for the robot-assisted gait rehabilitation of patients with a limited walking ability due to neurological damage to the central and peripheral nervous systems, as well as due to orthopaedic, geriatric and pediatric symptoms.

Depending on national differences, the LEXO® is typically used in a physiotherapy environment in order to support, enhance, and intensify therapy. The repetitive movements of the lower limbs, and consequently of the gait pattern, generate intrinsic and extrinsic stimuli which favour the reorganisation of the brain. Repeated, active practice and training encourages the neuronal plasticity and thus changes (adjustments) of synapses, nerve cells or even entire brain areas to recover lost movements. For more details, refer to the LEXO® user manual.

With the LEXO® it is possible to reach a high number of steps and intensity. The device is easy to handle and it takes not much time i.e., ~2 minutes to setup the patients.

Claims on clinical performance: The LEXO® is intended for the robot-assisted gait rehabilitation of patients with a limited ability to walk. The LEXO® system has an easy and fast setup, is an end-effector, enables active walking, gamification, has a compact design.

Main features:

- Weight support with harness system or saddle and trunk fixation
- Active 3D Pelvis
- Passive/Assistive/Active Walking
- Backrest for unstable patients
- Patient self-transfer and lifter transfer
- Variable step length, cadence, velocity (electrically adjustable)
- Augmented performance feedback

Results of a systematic evaluation of the specific scientific literature

- Walking ability (independent walking), gait velocity and maximum walking distance are central parameters for measuring the success of rehabilitation of gait after a stroke.
- The use of robotics can positively affect the outcome of a gait rehabilitation in patients with stroke (Bruni et al., 2018).

- People who receive electromechanical-assisted gait training in combination with physiotherapy after stroke are more likely to achieve independent walking than people who receive gait training without these devices (Mehrholz et al., 2017).
- The earlier the training starts, the better the gait recovery (Bruni 2018). People in the first three months after stroke and those who are not able to walk seem to benefit most from robot-assisted gait training (Mehrholz et al., 2017). Robotic rehabilitation is a valuable post-stroke treatment, leading to the best results in the subacute phase (Bruni et al., 2018).
- With respect to gait velocity, gait training assisted by end-effector apparatus (like the LEXO®) lead to a statistically significant and clinically relevant improvement in post-stroke patients, while none of the other interventions (i.e. no gait training, conventional gait training (reference category), training on a treadmill with or without body weight support, training on a treadmill with or without a speed paradigm, and electromechanically assisted gait training with exoskeleton apparatus) improved gait velocity to any significant extent (Mehrholz et al., 2018).

With respect to the maximum walking distance, gait training assisted by end-effector apparatus and treadmill training with body weight support led to a significant improvement.

2 PURPOSE, OBJECTIVES AND HYPOTHESES OF THE CLINICAL INVESTIGATION

2.1. Study purpose

The purpose of this clinical investigation is to investigate the feasibility and acceptability and compare the preliminary effects of RAGT a mixed population with neurological diseases consisting of patients with stroke in the subacute and chronic phases and with severe and mild to moderate disability, MS, Parkinson's Disease, spastic para-or tetraplegia, mild to medium grade hereditary ataxia, acute or chronic inflammatory demyelinating polyneuropathy and motoneuron disease. A further purpose of this clinical investigation is to synthesise quantitative and qualitative results for examining causality i.e., the extent to which the RAGT intervention may have caused the outcomes.

2.2. Primary objectives

The primary objective of this clinical investigation is to explore the feasibility of the methods and of a full-scale randomised controlled trial as assessed by the recruitment rate, retention rate and adherence rate, adverse events as monitored by a structured log, the acceptability of the intervention as evaluated by a Smiley Likert Scale asking four predefined questions, as well as a semi-structured observation during the RAGT intervention for understanding the intervention context and process.

2.3. Secondary objectives

Secondary objective 1 of this clinical investigation is to investigate the changes in the fall-rate after a 4-week RAGT as assessed narratively by the number of falls in ambulatory patients.

Secondary objective 2 of this clinical investigation is to investigate the changes in walking ability after a 4-week RAGT as assessed by the Functional Ambulation Categories (FAC).

Secondary objective 3 of this clinical investigation is to investigate the changes in walking speed after a 4-week RAGT as assessed by the 10-Metre Walk Test (10MWT) in ambulatory patients.

Secondary objective 4 of this clinical investigation is to investigate the changes in dynamic balance after a 4-week RAGT during walking as assessed by the Functional Gait Assessment (FGA) in ambulatory patients.

Secondary objective 5 of this clinical investigation is to investigate the changes in walking distance after a 4-week RAGT as assessed by the 6-Minute Walk Test (6MWT) in ambulatory patients.

Secondary objective 6 of this clinical investigation is to investigate the changes in functional mobility after a 4-week RAGT as assessed by the Timed Up and Go test (TUG) in ambulatory patients.

Secondary objective 7 of this clinical investigation is to investigate the changes in dynamic balance after a 4-week RAGT as assessed by the Four-Square Step Test (FSST) in ambulatory patients.

Secondary objective 8 of this clinical investigation is to investigate the changes in trunk movement after a 4-week RAGT as assessed by the Trunk Control Test (TCT) in non-ambulatory patients.

Secondary objective 9 of this clinical investigation is to investigate the changes in the limits of stability with respect to trunk movements after a 4-week RAGT as assessed by the Modified Functional Reach Test (MFRT) in non-ambulatory patients.

Secondary objective 10 of this clinical investigation is to investigate the changes in fall-related self-efficacy after a 4-week RAGT as assessed by the Falls Efficacy Scale- International Version (FES-I).

Secondary objective 11 of this clinical investigation is to investigate the changes in health-related quality of life (HRQoL) after a 4-week RAGT as assessed by the 5-level EQ-5D version (EQ-5D-5L).

Secondary objective 12 of this clinical investigation is to investigate the changes in depression after a 4-week RAGT as assessed by the Beck Depression Inventory, second edition (BDI-II).

Secondary objective 13 of this clinical investigation is to investigate the changes in fatigue after a 4-week RAGT as assessed by the Fatigue Severity Scale (FSS).

Secondary objective 14 of this clinical investigation is to explore the amount of individual and predefined goal achievement after a 4-week RAGT as assessed by Goal Attainment Scaling (GAS).

Secondary objective 15 of this clinical investigation is to explore the changes in gait parameters (gait speed, walking distance, number of steps and step length) over the RAGT intervention period as measured by the LEXO® robotic gait trainer.

Secondary objective 16 of this clinical investigation is to explore the changes in body weight and gait support (percentage of body weight support, percentage of active/passive gait modes) over the RAGT intervention period as measured by the LEXO® robotic gait trainer.

Secondary objective 17 of this clinical investigation is to develop a theory on the causal relationships between the RAGT and study outcomes with respect to the different study patient populations. This objective does not involve patient assessments.

2.4. Null-Hypothesis

A 4-week RAGT does not change the fall rate, functional ambulation, walking speed, dynamic balance, walking distance, functional mobility, trunk movement, limits of stability with respect to trunk movements, fall-related self-efficacy, HRQoL, depression, fatigue and gait parameters in people with neurological disorders (stroke, MS, Parkinson's disease, spastic para- or tetraplegia, hereditary ataxia, acute/chronic inflammatory demyelinating polyneuropathy and motor neuron disease).

3 INVESTIGATIONAL MEDICAL DEVICE

3.1. Description

A description of the medical device is provided according to EN ISO 14155 A.2 (International Organization for Standardization (ISO), 2018): The LEXO® is a mechatronic therapy system for body weight-supported gait rehabilitation of patients with a limited ability to walk. The LEXO® consists of a gait unit which includes an electrically driven movement mechanism, an electrically driven body weight support system with static and dynamic body weight support, an electrically driven pelvic guidance system and a computer-based control, and operating unit for setting the therapy parameters (see Figures 1 to 3 and Table 2).

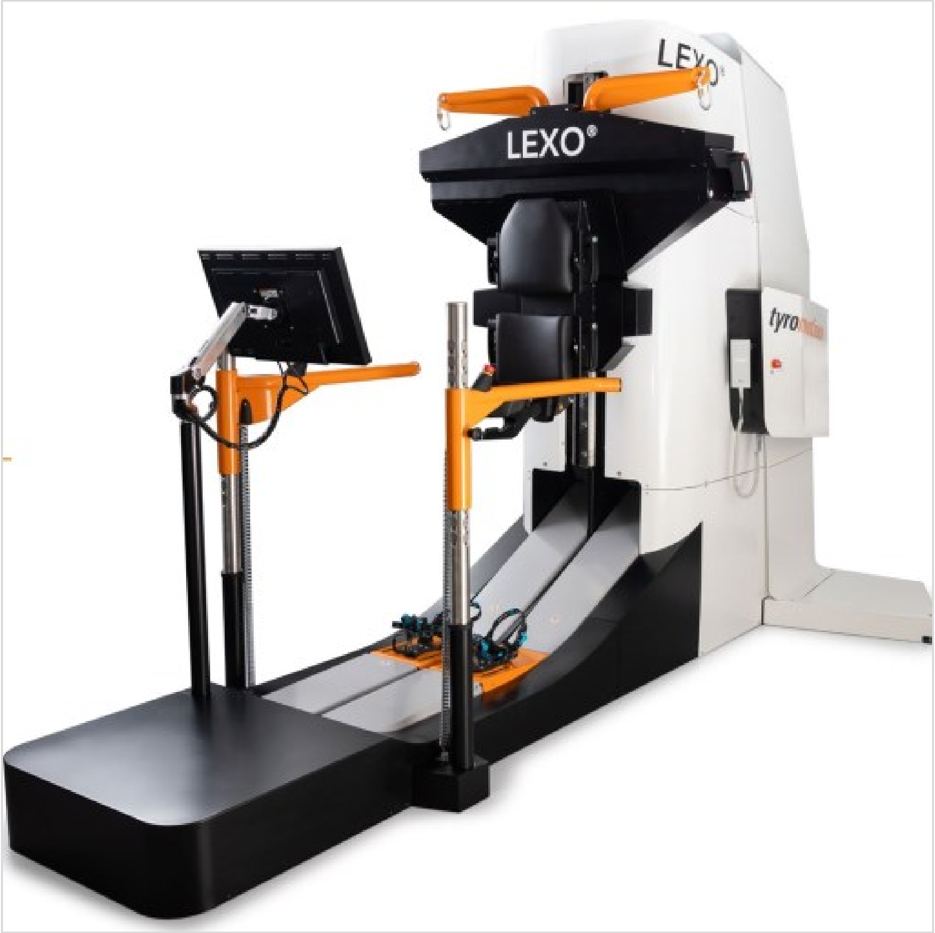

Figure 1: LEXO® robotic gait trainer overview

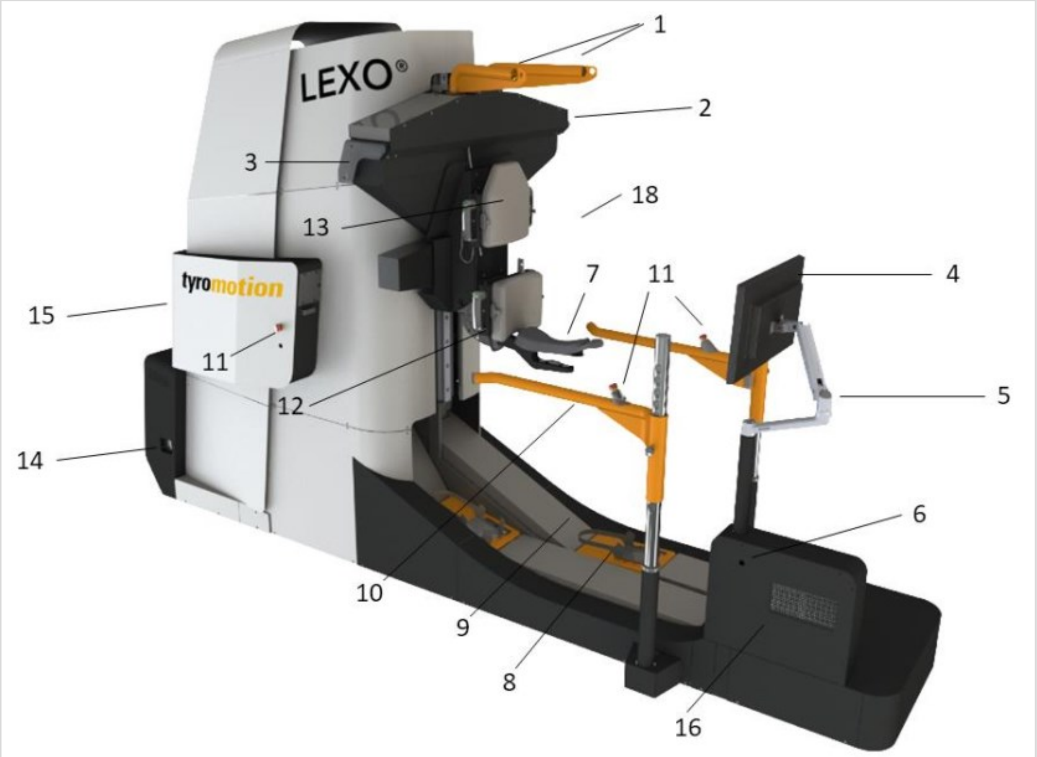

Figure 2: LEXO® robotic gait trainer, lateral front view

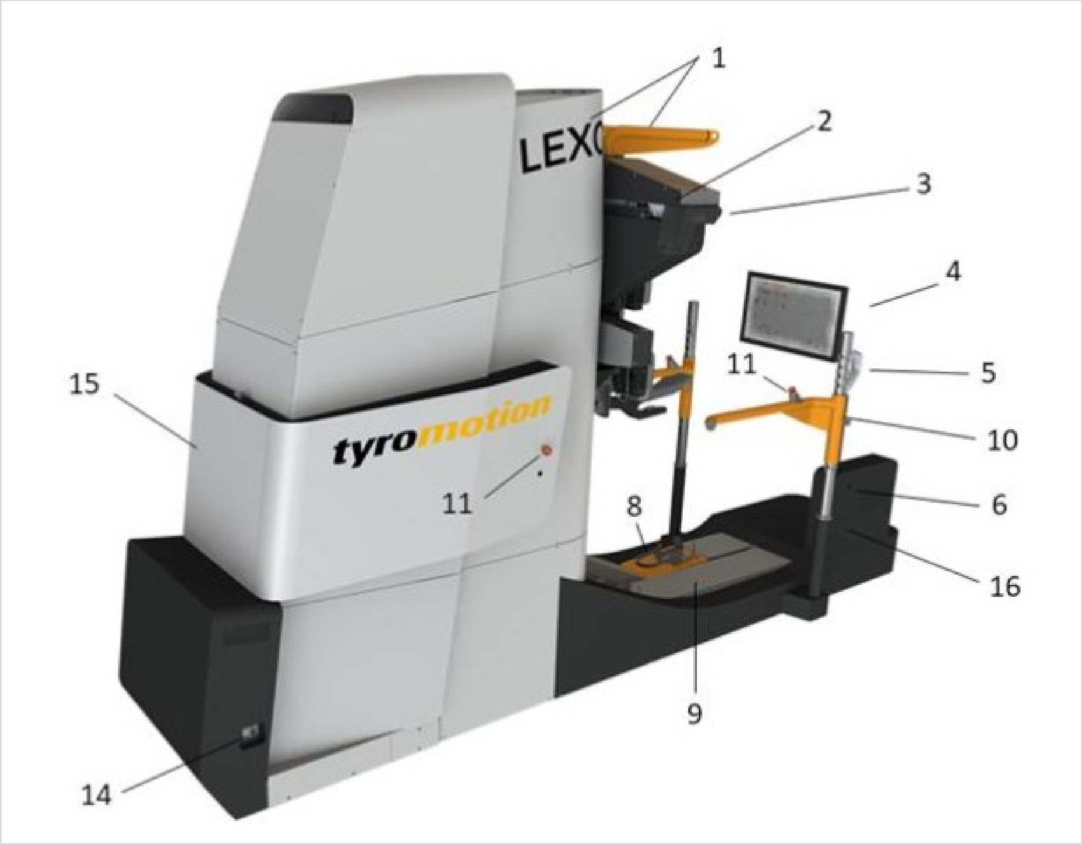

Figure 3: LEXO® robotic gait trainer, lateral rear view

Table 2: Components of the LEXO® robotic gait trainer as shown in Figures 2 and 3:

1	Swiveling harness suspension
2	Patient transfer system
3	Operating unit of the patient transfer system
4	Monitor
5	Pivoting monitor arm
6	On-Off switch PC
7	Removable saddle with sliding board attachment
8	Foot plate with foot fixation
9	Gait unit
10	Height-adjustable and swiveling handrails
11	EMERGENCY STOP button
12	Adjustable pelvic attachment for pelvic guidance
13	Adjustable back attachment
14	Power supply / control cabinet
15	Access for emergency lowering and crank handle
16	Access to USB ports (service purposes)

The LEXO® is a device for the rehabilitation of the relevant prerequisites of gait: balance, locomotion, musculoskeletal integrity, and neurocognitive control.

The LEXO® robotic gait trainer consists of the following components included in the scope of delivery:

- Monitor
- Pivoting monitor arm
- On-Off switch PC
- Foot plate with foot fixation
- Gait unit
- Height-adjustable and swiveling handrails
- EMERGENCY STOP button
- Adjustable pelvic attachment for pelvic guidance
- Adjustable back attachment
- Power supply / control cabinet
- Access for emergency lowering and crank handle
- Base unit
- BOX PC (built into LEXO) and touch screen
- Power cable
- Removable saddle with sliding board attachment
- Sliding transfer board for patient transfer
- Operating unit of the patient transfer system
- Swiveling harness suspension system with harnesses in 3 different sizes (S, M, L)
- Emergency lowering crank
- Lubricant: Bosch Rexroth Dynalub 510
- Lubrication press
- USB flash drive (User Manual and accompanying documents)
- Access to USB ports (service purposes)

A User Manual is provided as a hard copy

No medicinal substances, human or animal tissues or their derivatives, or other biologically active substances will be used in this clinical investigation.

3.2. Intended purpose

The intended purpose of the medical device as outlined in the technical documentation and reported in compliance with EN ISO 14155 A.2 a) (International Organization for Standardization (ISO), 2018) is as follows: The LEXO® is intended for the robot-assisted gait rehabilitation of patients with a limited ability to walk due to neurological damage to the central and peripheral nervous system as well as due to orthopaedic, geriatric and paediatric symptoms. Depending on national differences, the LEXO® is typically used in a physical therapy environment in order to support, enhance, and intensify therapy. The repetitive movements of the lower limbs, and consequently of the gait pattern, generate intrinsic and extrinsic stimuli which favour the reorganisation of the brain. Repeated, active practice and training encourages the neuronal plasticity and thus changes (adjustments) of synapses, nerve cells or even entire brain areas to recover lost movements.

Claims on clinical performance: The LEXO® is intended for the robot-assisted gait rehabilitation of patients with a limited ability to walk. The LEXO® system has an easy and fast setup, is an end-effector, enables active walking, gamification, has a compact design.

Main features:

- Weight support with harness system or saddle and trunk fixation
- Active 3D Pelvis
- Passive/Assistive/Active Walking
- Backrest for unstable patients
- Patient self-transfer and lifter transfer
- Variable step length, cadence, velocity (electrically adjustable)
- Augmented performance feedback

3.3. Manufacturer

Tyromotion GmbH, located in Graz, Austria is a leading manufacturer of technology-based therapeutic devices with the goal of improving independence and quality of life for people around the globe. Tyromotion develops, manufactures and sells therapy devices for neurorehabilitation and has more than 40 distribution partners and more than 4.000 installed devices worldwide. The devices are software-supported and contain actuators and sensors to document the success of the therapy and to get the most out of a therapy.

3.4. Identification and traceability of the investigational medical device

Possible Device Coding According to the Single National Classification (CND): GMDN CODE 47516; GAIT REHABILITATION SYSTEM, AUTOMATED-GUIDANCE

According to the Declaration of Conformity, the name of the investigational medical device is LEXO ® and the type is (14083) LEXO, including (14299) tyroS Software Version 6.1. Only one LEXO will be used in this clinical investigation and traceability will be achieved using its serial

number: LEX-02035. Systems Components are as follows: (-) Base Unit; (-) Box PC; (-) Saddle; (-) Transfer Board; (14528) Harness System S; (14527) Harness M; (14526) Harness L; (-) Emergency lowering crank; (14530) Harness XS; (14529) Harness XL and (-) Lexo VR Package.

3.5. Intended purpose in the clinical investigation

The intended purpose of LEXO ® in the clinical investigation will be to perform RAGT in patients with neurological disorders embedded into their inpatient rehabilitation, for a period of 4 weeks and 4 times per week for 45 minutes.

3.6. Population, indications and contraindications of the investigational medical device

Indications

The **functional indications** for movement therapy with the LEXO® robotic gait trainer are given according to the categories of the ICF (see Table 3).

Table 3: Indications for the movement therapy using the LEXO® robotic gait trainer according to the categories of the ICF (International Classification of Functioning, Disability and Health of the World Health Organization 2001).

IMPAIRMENTS OF BODY FUNCTIONS<br>Body functions are the physiological functions of body systems. Impairments are problems in body function as a significant deviation or loss.		
b1. Mental functions		
b140	Attention functions	Specific mental functions of focusing on an external<br>stimulus or internal experience for the required period of time<br>Functions of sustaining attention, shifting attention, dividing attention, sharing attention; concentration; distractibility
b156	Perceptual functions	Specific mental functions of recognizing and interpreting sensory stimuli<br>Functions of auditory, visual, olfactory, gustatory,<br>tactile and visuospatial perception
b7. Neuromusculoskeletal and movement-related functions		
b710	Mobility of joint functions	Functions of the range and ease of movement of a<br>joint

b730	Muscle power functions	Functions related to the force generated by the contraction of a muscle or muscle groups
b740	Muscle endurance functions	Functions related to sustaining muscle contraction<br>for the required period of time
b760	Control of voluntary movement functions	Functions associated with control over and coordination of voluntary movements.<br>Functions of control of simple voluntary movements and of complex voluntary movements,<br>coordination of voluntary movements, supportive<br>functions of arm or leg, right left motor coordination, impairments such as control and coordination problems
b770	Gait pattern functions	Functions of movement patterns associated with<br>walking. Impairments such as spastic gait, hemiplegic gait, paraplegic gait, asymmetric gait,<br>limping and stiff gait pattern
ACTIVITIES AND PARTICIPATION<br>Activity is the execution of a task or action by an individual. Participation is involvement in a life situation. Activity limitations are difficulties an individual may have in executing activities. Participation restrictions are problems an individual may experience in involvement in life situations.		
d4. Mobility		
d415	Maintaining a body position	d4154 Maintaining a standing position - Staying in<br>a standing position for some time as required
d450	Walking	Moving on foot, step by step.<br>d4500 Walking short distances - Walking for less<br>than a kilometer<br>d4501 Walking long distances - Walking for more<br>than a kilometer, such as across a village or town,<br>between villages or across open areas;
b770	Gait pattern functions	Functions of movement patterns associated with<br>walking. Impairments such as spastic gait, hemiplegic gait, paraplegic gait, asymmetric

		gait,<br>limping and stiff gait pattern

Population

One or more of the functional **indications** for using the LEXO® listed in Table 3 can occur in connection with the following **main diagnoses**:

- Stroke (cerebral hemorrhage, ischemic damage)
- Traumatic brain injury (TBI)
- Spinal cord injury (SCI)
- Paraplegia, tetraplegia
- Spastic and flaccid paralyses
- Cerebral palsy (CP)
- Spinal muscular atrophy
- Chronic disease, such as multiple sclerosis (MS)
- Parkinson's disease (PD)
- Cardiovascular disease as far as permitted by the attending medical expert
- Motor neuron disease, e.g., amyotrophic lateral sclerosis (ALS)
- Postoperative rehabilitation, e.g. After knee or hip joint replacement (K-TEP, H-TEP), condition after the reconstruction of the cruciate ligament
- Fractures and injuries of the lower extremity (remodeling phase)
- Degenerative joint disease of the lower extremity (e.g., arthrosis)
- Myopathy
- Muscular dystrophy
- Muscle weakness due to a lack of mobility
- Amputation
- Edema

Just as for any other therapy, the attending healthcare professional is responsible for the medical diagnosis, indication and selection of the appropriate therapy. Knowing the contraindications is essential to not put patients at risk. It must be noted that patients may also have additional indications and/or contraindications which are not listed here, but are still relevant, and that the following list is not exhaustive. In the event of uncertainties or feedback, TYROMOTION GmbH will be contacted by the study PI.

Absolute contraindications

The device must not be used if the patient has any of the following symptoms:

- Acute, pronounced pain symptoms despite conventional pain therapy
- Adaptability and patient's position: Do not carry out any training session using the system if it is not possible to adapt the device to the individual physiological patient's position (e.g., in the event of contractures or severe spasticity (stiff/immobile joint) in

the area of the lower extremity, extremely disproportionate growth of the legs and/or spinal column).

- Body weight of less than 15 kg or more than 180 kg
- Body height of less than 100 cm or more than 200 cm
- Insufficient compliance e.g., patients with a serious mental illness or severe neurosis
- For patients with a significant reduction in bone density (osteopenia or osteoporosis), the training is contraindicated. Due to the forces acting on the lower extremities, LEXO® training must not be carried out for patients whose history indicates a significant loss of bone density or an increased risk of fracture (e.g., for people with long-term limited weight bearing), and/or for patients who have already been diagnosed with a significant loss of bone density.
- Osseous instability (non-consolidated fractures, osteogenesis imperfecta, unstable spinal column, pseudoarthrosis)
- Cardiac contraindications
- Use of ventilators
- Highly pronounced vascular diseases of the lower limbs
- High-grade ataxia
- Skin lesions in areas which get in contact with the device or the harness system (decubitus)
- Severely limited range of movement of the lower extremity (e.g., fixated joint contractures, joint arthrodesis) for which even the lightest passive movement training already represents a risk of injury
- States of health preventing active rehabilitation (e.g., respiratory diseases, pregnancy, orthopedic diseases, cognitive impairments restricting communication, neuropsychological disorders, infections or inflammatory diseases, osteomyelitis)

Risk factors (relative contraindications)

Each patient must be assessed individually by the study PI to determine whether the LEXO® robotic gait trainer is suitable for the patient in the event of:

- Apraxia
- Arthritis in the area of the joints of the lower extremity
- Risk of autonomic dysreflexia (AD) in patients with spinal cord injury (SCI) (severity TH6 or above; an AD occurred in the past increases the risk of a new episode)
- Reduced compliance, such as patients with cognitive impairments, medication
- Uncooperative or (self)aggressive behavior (e.g., transitory psychotic
- syndrome)

- Cardiac diseases, e.g., heart failure and thoracotomy, uncontrolled orthostatic hypotension or other circulatory problems, circulatory disorders of the lower limbs
- Recent joint injury/endoprosthetics (e.g., total endoprosthetics, knee or hip joint replacement, reconstruction of the cruciate ligament or meniscus) with contraindicated ranges of movement or load limits
- Consolidated fractures in the area of the lower extremity
- Epilepsy
- Infections/swellings/skin ulcers/late effects of previous injuries, especially of the lower extremity
- Recently occurred seizures or increased risk of seizures
- Mechanical ventilation
- Lack of head control
- Long-term infusions (e.g., Baclofen pump, intrathecal pumps, PEG tube …) or
- stimulators (e.g., pacemakers, nerve stimulators)
- Enterostomy/ostomy/anus praeter
- Osteoporosis
- Sensory impairments of the lower extremities and the torso, especially reduced sensation of pain. Patients with sensory impairments might not be able to give feedback on possibly occurring pain.
- Skin problems: Before and after every training session, it is a requirement to watch out for wounds and pressure marks either resulting from the training or that were already there, especially in those areas of the body regions which are in contact with the device.
- Pregnancy
- Uncontrolled instability of the hip, knee or ankle joint which would represent a risk despite using the body weight support.

3.7. Training, usage and safety aspects of using the investigational medical device

Tyromotion Training concept with respect to usage of the LEXO®

The LEXO® is a complex, technical device. For the safety of the patients, the users and the device itself as well as a successful training, users of the LEXO® gait trainer must be trained beforehand have read the user manual. Reading the manual alone does not convey enough competence for handling the LEXO® gait trainer.

Trained operators, who are responsible for the LEXO®, are obliged to urge personnel, patients, and other persons who are in the environment of the device to fully comply with the safety precautions stated herein. The system may only be operated by properly trained operators. It will be ensured that the system is not manipulated by unauthorised persons. The system must (and will) be unpacked and installed by service representatives authorised by TYROMOTION GmbH only. The system will exclusively be trained by TYROMOTION GmbH authorized trainers. Independent commissioning of the device before training is not permitted and will not be done.

Prospective users must have basic medical training as a prerequisite (e.g., physiotherapy). TYROMOTION GmbH rejects any liability for damage that arose in connection with a training session that was not performed by a trained user. The delivery of the LEXO® includes a structured training for future users. In the case of this clinical investigation, extra training will be provided for a selected team of physiotherapists of the Reha Zentrum Münster to guarantee an optimal use of the device during the clinical investigation. In addition, users (physiotherapists within the clinical investigation team) will perform repeated trainings with the LEXO®. Users must not allow other people to use the LEXO® or instruct other members of the rehabilitation team. Users will be trained by a member of TYROMOTION GmbH or through a trained instructor delegated by TYROMOTION GmbH. The study team will also be thoroughly informed on any contraindications in order to not carrying out the therapy using the LEXO®.

The training content will include:

- LEXO® system / component overview
- Function and application overview
- Indications and contraindications
- Adjustment options and safety instructions for gear unit
- Connection system
- Transfer to the LEXO® system
- Therapy with the LEXO® system
- Functionality of the emergency lowering system and warning notices
- Cleaning the device

Safety aspects of using LEXO®

Any warnings with respect to a safe use of LEXO® are specified in detail in the user manual. Additionally, the patient will be explained that he or she may use an emergency STOP button to interrupt the power supply of the drives. The study team will undergo a structured training by Tyromotion.

Residual risk

Despite all safety precautions, there can be unforeseeable residual risks when carrying out a therapy session using the LEXO®. Even if the device is used properly, the patient or operator may still suffer from minor pinch or crush injuries in rare cases. A detailed risk analysis has been described in Chapter 1.2.

3.8. Device approval status

The LEXO® robotic gait trainer is approved in Austria and has gained an EC-Certificate regarding a Full quality assurance system. The certificate states that Tyromotion has implemented and maintains a full quality assurance system which applies to the products at every stage from design to final controls.

Through an audit, documented in a report, it was verified that the management system fulfills the requirements of Annex II – excluding Section 4 of Council Directive 93/42/EEC concerning medical devices with respect to the LEXO®. The LEXO is classified as a class IIa medical device.

As stated in Chapter 3.4. “Identification and traceability of the investigational medical device”, according to the Declaration of Conformity the name of the investigational medical device is LEXO®.

Conformity has been assessed in compliance with RL 93/42/EWG/ processes (RL 93/42/EEC Annex II without Sec. 4). The EC-Verification Certificate, Annex II.

Compliance with harmonized standards was assessed and demonstrated as shown in Table 4. The manufacturer Tyromotion declares under sole responsibility that the LEXO® as delivered are in compliance with directive 93/42/EEC and the Austrian medical- device-law BGBl. 657/1996. The LEXO® is CE marked.

Table 4: Used harmonized standards

**EN 60601-1:2006 + Cor.:2010 +A1:2013**<br>**EN ISO 13485:2016 + AC:2016**<br>**EN 14971:2012**<br>**EN 62366-1:2015 + A1:2015**<br>**EN 62304:2006 + Cor.:2008 + A1:2015**	**EN 60601-1-2:2015**<br>**EN ISO 10993-1:2009**<br>**EN ISO 15223-1:2016**<br>**EN 1041:2008+A1:2013**

4 STUDY DESIGN AND PROCEDURES

4.1. General aspects, justification and study related procedures

This clinical investigation protocol follows the Standard Protocol Items for Clinical Trials (SPIRIT; Statement 2013) (Chan et al., 2013), the World Medical Association (WMA) Declaration of Helsinki - Ethical Principles for Medical Research involving human subjects (2013) (WMA, 2013), the Guidelines of the International Organization for Standardization (ISO), ICH E6 Guideline for good clinical practice (2016) and Guideline for Clinical investigation of medical devices for human subjects - Good clinical practice (ISO/DIS 14155:2018) (2018).

A prospective comparative multiple case study design with mainly quantitative data but also qualitative data analyses and a cross-case analysis will be used. A multiple case-oriented research strategy will be adopted to acquire knowledge about the outcomes from RAGT within the 8 different patient (disease) groups, learn about similarities and differences between individual cases and neurological disorders within study intervention periods and emerging patterns (Ababacar et al., 2020; Nordbeck, 2013). The synthesis across cases will extend beyond the comparison of similarities and differences to generate information on the reasons and explanations as to the findings from the RAGT intervention. That is, the prospective comparative multiple case study design will allow for theory development with regard to causality to inform the hypotheses of a follow-up randomised controlled trial (Goodrick, 2014). In other words, except some degree of comparison that will be fundamental to the comparative multiple case study design, a distinctive feature of this study type is the emphasis on examining causality i.e., the extent to which the RAGT intervention caused the outcomes (Goodrick, 2014).

In practice, this comparative multi case study will involve the analysis and synthesis of the similarities, differences and patterns across multiple cases, with the shared common goal of gaining knowledge about the feasibility, acceptability and preliminary effects of RAGT. Case selection will be performed using two different strategies: within disease groups, the 'Most Similar Systems Design' (MSSD) will be applied and between disease groups, the 'Most Different Systems Design' (MDSD) (Anckar, 2008; Przeworski & Teune, 1970) will be used, considering the 8 neurological disease groups with different symptom complexes involving distinct central and peripheral nervous system (CNS, PNS) sensorimotor system areas and pathways (upper and lower motor neurons including both the cerebrum and spinal cord, extra pyramidal motor system, peripheral nervous system and cerebellum). As for MSSD, it will mean to purposively choose study patients that are similar in as many background characteristics as possible, but without systematically matching cases on all the relevant control variables. As for MDSD, the strategy will be to purposively select patients that are as different as possible with respect to extraneous variables. The basic logic is that differences cannot explain similarities (Przeworski & Teune, 1970). Both a quantitative and qualitative data collection and analyses will be used to achieve these goals.

All cases will receive RAGT training using a standard protocol that will allow individualisation according to the neurological disease, functional status and walking performance of the patient, the stage of the disease, age of the patient etc. No control intervention will be used because this is the first study to compare a LEXO-based RAGT in patients with 7 different neurological disorders, one of them including patients at different stages of recovery and functional status (subacute, severe stroke and chronic, mild to moderate stroke).

Figure 4 presents a sample flow diagram of the clinical investigation including analyses.

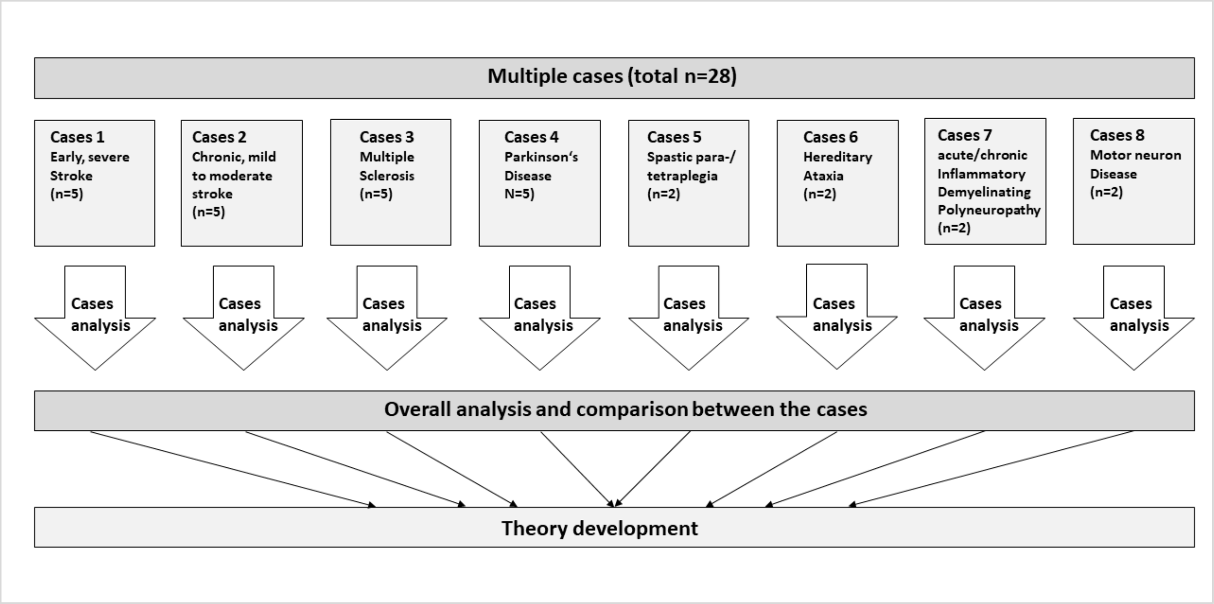

Figure 4: Sample flow diagram of the clinical investigation including analyses

4.2 Clinical investigation site

The clinical investigation will be performed at the Reha Zentrum Münster Betriebs GmbH, Gröben 700, 6232 Münster, Austria.

4.3 Responsible ethics committee

The responsible ethics committee (EC) for this clinical investigation is the Ethics Committee of the Medical University of Innsbruck.

4.4 Study registration

Once a favourable opinion has been obtained from the ethics committee, an application will be submitted for inclusion in the German Clinical Trials Register (DRKS) or another primary register in accordance with WHO criteria

4.5 Data collection

After eligibility screening, a screening for cognitive impairment (Scr) will be performed. In eligible patients who pass the cognitive screening, study specific assessment data will be collected at baseline (T1) and post-intervention (T2) at 4 weeks after completion. Assessments will be performed at similar daytimes to increase comparability. Information on demographic and disease specific patient characteristics will be extracted from patients' charts. Further disease-specific data will be collected by neurologists (PI, investigator), cognitive screening will be performed by psychologists as part of the clinical routine, and movement and function-related data will be collected by trained physiotherapists. The study participants will be

supported as required and as permissible with respect to the test instructions, and they may take a rest at any time.

4.6 Screening data

Screening for cognitive and walking impairment will be performed using two assessment strategies for each as presented in Table 5. The Mini Mental State Examination and a neurological exam are ***performed within clinical routine*** at the Reha Zentrum Münster ***(highlighted green)***, the Functional Ambulation Categories are performed ***by the study PI or investigator as part of the clinical investigation (highlighted yellow) and does not involve any activity of the patient***.

Table 5: Screening

Parameters	Assessment	Patient inclusion if	Scr
Cognitive function	Mini Mental State Examination (MMSE)	≥24/30 points	x
Walking impairment	Neurological exam	Walking impairment confirmed	x
Walking impairment	Functional Ambulation Categories (FAC)	0-4 points	x

Abbreviation: Scr=Screening.

4.7 Demographic and disease-specific data

Standardised assessments for rating walking and overall motor ability, spasticity, overall impairment or disability in the different patient groups will either be ***collected in routine care (highlighted green)*** or ***as part of the clinical investigation (highlighted yellow)*** (see Table 6 for details). Disease specific instruments will be used as appropriate.

Table 6: Demographic and disease specific information checked at eligibility screening and collected at baseline

Parameters	Assessment (if applicable)	BL
Age (years; date of birth)	--	x
Gender: male, female, diverse	--	x
Education (years)	--	x
Diagnosed disease	As per defined criteria*	x
Date of initial diagnosis	--	x
Most affected body part	--	x

Impairment caused by a stroke	National Institutes of Health Stroke Scale, or NIH Stroke Scale (NIHSS) (Brott et al., 1989; Lyden et al., 1999), validated German version (Berger et al., 1999)	x
Disability caused by MS	Expanded Disability Status Scale (EDSS) (Kurtzke, 1983)	x
Disability and impairment in PD	Unified Parkinson's Disease Rating Scale (UPDRS) (Fahn & Elton, 1987)	x
Functional disability caused by PD	Hoehn & Yahr Scale (H&Y) (Hoehn & Yahr, 1967)	x
Neurological level of injury in SCI	As per defined criteria*	x
Classification of the acquired impairment in SCI	American Spinal Injury Association (ASIA) Impairment Scale (AIS) (Kirshblum et al., 2011; Roberts et al., 2017) in its German version ("Traumatische Querschnittlähmung," 2019)	x
Impairment caused by ataxia due to MS, hereditary ataxia, GBS or stroke	Scale for the Assessment and Rating of Ataxia (SARA) (Schmitz-Hübsch et al., 2006; Weyer et al., 2007).	x
Spasticity caused by a stroke, MS, spastic para- and tetraplegia or motor neuron disease	Modified Ashworth Scale (MAS) (Bohannon & Smith, 1987; Harb & Kishner, 2021)	x
Disability caused by GBS	Guillain-Barré syndrome disability scale (GBSDS) (van Koningsveld et al., 2007) in its validated German version (aus: (*Diagnose und Therapie des Guillain-Barré Syndroms im Kindes- und Jugendalter, 4. Auflage, Version 1.0*))	x
Peripheral neuropathy e.g., due to GBS or CIDP	Overall Neuropathy Limitations Scale (ONLS) (Graham & Hughes, 2006)	x
Functional status and functional change in patients with ALS	Amyotrophic Lateral Sclerosis Functional Rating Scale, revised version (ALS-FRS-R) (Cedarbaum et al., 1999), in its validated German version (Abdulla et al., 2013)	x

Abbreviation: BL=baseline.
*All diagnoses are made based on a clinical exam of the patient and supportive investigation: (1) Stroke is diagnosed based on current criteria according to the Guideline of the German Society of Neurology (2017) (Hennerici & Kern, 2017)

(2) MS is diagnosed according to the revised McDonald criteria valid at the time of diagnosis (Polman et al., 2011; Thompson et al., 2018).
(3) (Idiopathic) PD is diagnosed according to the UK Brain Bank criteria (Hughes et al., 1992).
(4) SCI is diagnosed based on current criteria according to the Guideline of the German Society of Neurology (DGN 2012) or current NICE Guideline (*Spinal injury: assessment and initial management. Spinal injury assessment: assessment and imaging for spinal injury. NICE Guideline NG41*, 2016) valid at the time of diagnosis.
(5) Hereditary ataxia is diagnosed according to the Ataxia Medical Guidelines 2016 or earlier versions, developed by Ataxia UK, London (de Silva et al., 2019).
(6) For GBS, the current diagnostic criteria are used (Willison et al., 2016). Chronic inflammatory demyelinating polyneuropathy (CIDP) is diagnosed according to diagnostic criteria of the European Federation of Neurological Societies/Peripheral Nerve Society Guideline on the management of chronic inflammatory demyelinating polyradiculoneuropathy- First Revision ("European Federation of Neurological Societies/Peripheral Nerve Society Guideline on management of chronic inflammatory demyelinating polyradiculoneuropathy: report of a joint task force of the European Federation of Neurological Societies and the Peripheral Nerve Society--First Revision," 2010) or earlier versions.
(7) Motoneuron disease is diagnosed using the diagnostic criteria for ALS from the World Federation of Neurology as the El Escorial Criteria (Brooks, 1994), in their revised version as the Airlie House criteria (or El Escorial revised) (Brooks et al., 2000) and the Awaji criteria (de Carvalho et al., 2008). There are no formal criteria for progressive muscular atrophy (PMA) and primary lateral sclerosis (PLS). Recently, the Gold Coast criteria were developed and are proposed (Johnsen, 2020).

4.8 Primary and secondary endpoints

Primary endpoint

The primary endpoint of this clinical investigation is the **feasibility** of the methods and of a full-scale randomised controlled trial. This will be assessed by the recruitment rate, retention rate and adherence rate, a structured adverse events log, a Smiley Likert Scale asking four predefined questions on the acceptance of the intervention, as well as a semi-structured observation during the RAGT intervention for understanding the intervention context and process.

Recruitment, retention and adherence rates

The feasibility of the methods and of conducting a larger trial will be explored using predefined criteria, recording monthly recruitment and adherence rates to the RAGT intervention. Using a structured log, adherence to the RAGT will be recorded, including reasons (e.g., discontinuation due to lack of interest or injury). Any non-retention will also be recorded with reasons (e.g., consent withdrawn). ***This evaluation will not involve any***

additional measures for the patients.

The criteria for feasibility success will be:

a) a target recruitment rate 3 patients per month.,

b) a target retention rate of 85%,

c) a target minimum adherence rate of 75% to the RAGT interventions (12 sessions out of a maximum of 16)

Adverse events

Any adverse events and side effects will be systematically recorded and evaluated using a formal log including the type of adverse event, dates, severity, causality differentiated into serious/non-serious, actions to restore or improve the patient's wellbeing and outcome of the event. Study related adverse events such as training-related injuries will be treated and paid for. Severe adverse events will lead to early clinical investigation termination. ***This monitoring will be performed throughout the study.***

Acceptability of the intervention

Acceptability of the intervention will be assessed using a Smiley Likert Scale asking four predefined questions on the acceptance of the intervention (please see CRF), as well as a semi-structured observation during the RAGT intervention for understanding the intervention context and process, recognising patterns, and moving beyond selected outcome measures. This is of clinical relevance because high acceptability may help increase motivation and adherence in patients. ***The Likert scale will be used as part of the clinical investigation (2-4 min).***

Secondary endpoints

The secondary endpoints for this clinical investigation and how they will be assessed are presented in Table 7. The validated German version of all assessments will be used, which have demonstrated good to excellent properties. In addition, the collection timepoints are shown. The assessments which will be ***performed in clinical routine are highlighted in green***, with the remaining assessments being ***performed as part of the clinical investigation (highlighted yellow).***

Table 7: Secondary endpoints and assessment

No.	Secondary endpoint	Assessment	Scr	BL	PI	IV
1.1	Number of falls	Falls log [ambulatory patients] (see CRF)		x	x	
1.2	Walking ability	Functional Ambulation Categories (FAC) [ambulatory patients]	x	(c)	x	

1.3	Walking speed	10-Metre Walk Test (10MWT) [ambulatory patients, assessed in clinical routine]		x	x	
1.4	Dynamic balance during walking	Functional Gait Assessment (FGA) [ambulatory patients]		x	x	
1.5	Walking distance	6-Minute Walk Test (6MWT) [ambulatory patients, assessed in clinical routine]		x	x	
1.6	Functional mobility	Timed Up and Go test (TUG) [ambulatory patients]		x	x	
1.7	Dynamic balance	Four-Square Step Test (FSST) [ambulatory patients]		x	x	
1.8	Trunk movement	Trunk Control Test (TCT) [non-ambulatory patients]		x	x	
1.9	Limits of stability with respect to trunk movements	Modified Functional Reach Test (MFRT) [non-ambulatory patients]		x	x	
1.10	Fall-related self-efficacy	Falls Efficacy Scale- International Version (FES-I)		x	x	
1.11	Health-related quality of life	5-level EQ-5D version (EQ-5D-5L)		x	x	
1.12	Depression	Beck Depression Inventory, second edition (BDI-II)		x	x	
1.13	Fatigue	Fatigue Severity Scale (FSS)			x	
1.14	Goal definition and evaluation of its achievement	SMART defined movement-related individual goal (a); Goal Attainment Scaling (GAS) (b)	ax		bx	
1.15	Gait parameters (gait speed, walking distance, number of steps and step length)	Measured by LEXO® during the intervention as part of the clinical routine				x
1.16	Body weight and gait support (percentage of body weight support, percentage of active/passive gait modes)	Measured by LEXO® during the intervention as part of the clinical routine				x

Abbreviation: Scr=screening; SMART=specific, measurable, attractive, realistic and terminated; c=data carryover to baseline if patient has passed the sreening; BL=baseline; PI=post-intervention; IV=intervention period (at all intervention sessions)

Standardised assessment description

All chosen standardised assessments are valid, reliable and responsive to change. Validated German versions will be used as can be seen from the CRF.

The Functional Ambulation Categories (FAC) (Mehrholz et al., 2007; Wade, 1992) will be used in its validated German version (Klotz et al., 2019) to screen patients with respect to their walking ability and to assess walking ability post-intervention. The FAC is a 6-point functional walking test that evaluates ambulation ability, determining how much human support the patient requires when walking, regardless of whether or not they use a personal assistive device. The FAC is a quick visual measurement of walking, is simple to use, easy to interpret, and cost-effective because only stairs and 15m of indoor floor are needed to administer the test

The 10-Metre Walk Test (10MWT) (Bohannon, 1997) is a performance measure used to assess walking speed in meters per second over a short distance. It can be employed to determine functional mobility in various neurological populations. The 10MWT was originally developed to measure walking capacity in patients with stroke (Wade et al., 1987) and was shown to be valid and reliable in a mixed group of neurological patients (Rossier & Wade, 2001). Its psychometric properties have been well investigated in adult patients with incomplete spinal cord injury showing good reliability and excellent responsiveness (van Hedel et al., 2006; van Hedel et al., 2005). In these patients, walking speed could also predict functional walking performance well (van Hedel, 2009). In adult patients with stroke, the 10MWT proved sensitive to change (Salbach et al., 2001). The required equipment includes a stopwatch and a clear pathway with set a distance of 10 metres. The set-up involves marking the 10-metre walkway and adding a mark at 2 and 8 metres. Instructions are as follows: The patient walks without assistance for 10 metres, with the time measured for the intermediate 6 meters to allow for acceleration and deceleration. Assistive devices may be used but must be kept consistent and documented for each test. Timing is started when the toes pass the 2-metre mark and timing is stopped when the toes pass the 8-metre mark. The 10MWT can be tested at either preferred walking speed or maximum walking speed. In this clinical investigation the test will be performed at maximum speed. Two trials will be performed and the average calculated (Watson, 2002).

The Functional Gait Assessment (FGA) (Wrisley et al., 2004) will used to assess dynamic balance during walking and assess a patient's ability to perform multiple motor tasks while walking. The FGA consists of 10 items. The items address walking on a surface without incline at different speeds, with head turns, with quick turns and stops, climbing stairs and climbing obstacles. The individual items are assessed on a scale of 0-3 points, with 3 points representing the best possible performance of the task. The total number of points is 30. The more points are achieved, the better the dynamic balance of the test subjects. The implementation takes 5-20 minutes (Thieme et al., 2009).

The 6-Minute Walk Test (6MWT) will involve participants covering as much ground as possible in 6 minutes, with or without their assistive devices and without physical assistance, along a 30-metre hallway, with small cones at each end to mark the turnaround points. Warm-up will not be allowed before the test, as recommended by the American Thoracic Society (A. T. S.) guidelines for the 6MWT (2002). Participants will rest on a chair for at least 10 minutes, close to the starting line of the test. A second chair for rest will be positioned close to the end of the hallway, but out of the walking way. Assistive devices can be used, but will be kept consistent and documented from test to test. Participants will be instructed to turn at each end; rests are allowed at any time, and the number and duration of rests during the 6 minutes are measured (Potter et al., 2014). Standard phrases of encouragement by use of an even voice are provided every minute, but no other words of encouragement or body language are used (A. T. S. Committee on Proficiency Standards for Clinical Pulmonary Function Laboratories, 2002). The number of laps from the starting point is counted and recorded visible to the participant, and the number of metres in the final partial lap distance is marked after 6 minutes. The residual walking distance is measured with a calibrated measuring wheel.

The Timed-Up and Go (TUG) will be used to measure functional mobility (Podsiadlo and Richardson, 1991). The TUG can be used as an indicator of the risk of falling during everyday movement tasks. The activities tested are transferring from sitting to standing, walking, turning around in space, going back to the chair and sitting down. For everyday locomotion and reducing the risk of falling, the skills of transferring from sitting to standing and turning in space are of crucial importance (Cheng et al., 1998; Mong et al., 2010). The test begins in the seat, on a chair with a seat height of approx. 46 cm. For the implementation of the TUG, the time that the patient needs to get up from the chair, walk a distance of three meters, turn around, go back three meters to the chair and get back into it, is stopped to move to the starting position. The time required is documented in seconds. Walking aids may be used. All aids used must be documented. The TUG takes less than 5 minutes to complete.

The Four Step Square Test (FSST) (Dite and Temple, 2002) assesses balance and the ability to climb over obstacles forwards, sideways and backwards. The patients stand in a square created by a marked test cross on the floor (see CRF). The patients are asked to step clockwise and then counterclockwise into all four squares. To do this, they have to step forward, to the side and backwards over the marked test cross. There are always two runs, a test run and a run in which the time is stopped. The implementation time is less than 5 minutes.

The Trunk Control Test (TCT) (Franchignoni et al., 1997) will be used in its German version (Fischer, 2014) to assess trunk mobility in severely affected, non-ambulatory stroke patients in the subacute stage. The TCT assesses four movement activities: rolling from a supine position to the weak side (1) and to the strong side (2), sitting up from a lying-down position (3), and sitting balance (4). The scoring is as follows: 0, unable to perform movement

without assistance; 12, able to perform movement but in an abnormal manner; and 25, able to complete movement normally. The TCT score is the sum of the scores obtained on the four tests (range, 0 to 100) (Franchignoni et al., 1997; Hsieh et al., 2002).

The Modified Functional Reach Test (MFRT) will be used to assess the limits of stability with respect to trunk movements in severely affected, non-ambulatory stroke patients in the subacute stage. The MFRT is a modified version of the Functional Reach Test (Duncan et al., 1990) assessing the limits of stability by measuring the maximum distance a patient can reach forward while sitting in a predefined position (Katz-Leurer et al., 2009; Persson et al., 2014). The MFRT is performed using a leveled meterstick attached to the wall at the height of the patient’s acromion level of the non-affected arm while sitting on a chair near the wall (Katz-Leurer et al., 2009). The patient is seated with hips, knees and ankles positioned at 90° of flexion, and feet positioned flat on the floor. Initial reach is measured with the patient sitting against the wall with an upper limb flexed to 90°. The initial reach is measured with the patient sitting against the back of the chair with the upper limb flexed to 90 degrees, measure is taken from the distal end of the third metacarpal along the meterstick. Testing consists of three conditions, each containing three trials: Sitting with the unaffected side near the wall and leaning forward (1); sitting with the back to the wall and leaning right (2) and sitting with the back to the wall leaning left. Instructions include leaning as far as possible in each direction without rotation and without touching the wall. The distance is recorded in centimetres covered in each direction. If the patient is unable to raise the affected arm, the distance covered by the acromion during leaning is recorded. The first trial in each direction is a practice trial and is not included in the final result. A 15 second rest break is provided between trials (Katz-Leurer et al., 2009).

A large number of people with neurological disabilities and older people have psychological problems associated with falls. This applies both to patients who have already fallen and to those who have not yet fallen. In order to be able to address these problems, an assessment of the fear of falling and of self-efficacy in relation to falling is required. The Falls Efficacy Scale (Tinetti et al., 1990) was expanded to an international version (FES-I) in 2005 by an expert network for fall prevention (Prevention of Falls Network Europe, ProFaNE) (Yardley et al., 2005; Skelton et al., 2004). The PROM is used to record the fall-associated self-efficacy in patients. With the expansion, more complex, functional activities and social aspects of self-efficacy were added. The FES-I consists of 16 items that evaluate concerns about falls and various activities among the subjects. The questionnaire can be carried out as a self-assessment form or as a structured interview. The answers are classified using a 4-point Likert scale from 1 = “no concerns” to 4 = “very serious concerns”. The duration of the questionnaire administration is 5-10 minutes.

The 5-level EQ-5D version (EQ-5D-5L) (Herdman et al., 2011) will be used in its validated German version (Ludwig et al., 2018; Ludwig et al., 2017) to assess health-related quality of life (HRQoL). The EQ-5D-5L was introduced by the EuroQol Group in 2009 to improve the

instrument's sensitivity and reduce ceiling effects, as has been observed for the EQ-5D-3L. The EQ-5D-5L essentially consists of 2 pages: the EQ-5D descriptive system and the EQ visual analogue scale (EQ VAS). The descriptive system comprises five dimensions: mobility, self-care, usual activities, pain/discomfort and anxiety/depression. Each dimension has 5 levels: no problems, slight problems, moderate problems, severe problems and extreme problems. The patient is asked to indicate his/her health state by ticking the box next to the most appropriate statement in each of the five dimensions. This decision results in a 1-digit number that expresses the level selected for that dimension. The digits for the five dimensions can be combined into a 5-digit number that describes the patient's health state. The EQ VAS records the patient's self-rated health on a 0-100 vertical visual analogue scale, where the endpoints are labelled 'The best health you can imagine' and 'The worst health you can imagine'. The VAS can be used as a quantitative measure of health outcome that reflect the patient's own judgement.

The BDI-II (Beck et al., 1988) will be used to measure the severity of depression and is a secondary endpoint of this clinical investigation. The BDI-II consists of 21 items and is a PROM for recording the severity of the depression. The items are evaluated using a 4-point Likert scale from 0 to 3. This means that the sum of the items can be 0-63 points. The scale is validated from the age of 13 years. The duration of the questionnaire administration is 5-10 minutes.

The Fatigue Severity Scale (FSS) (Krupp et al., 1989) will be used in its validated German version (Valko et al., 2008) to assess fatigue in this clinical investigation. The FSS is one of the most frequently used inventories for measuring fatigue in people with chronic illnesses including those with neurological diseases. The original FSS is a nine-item unidimensional questionnaire. Each item consists of statements that are scored on a seven-point Likert type scale ranging from 1 ('strongly disagree') to 7 ('strongly agree'). The mean score of the items will be used as the FSS score and a cut-off score of 5 to indicate substantial fatigue (Pfeffer, 2008).

Goal Attainment Scaling (GAS) is an example of an individualised evaluative instrument, which is used for measurement of changes in individual patients and groups of patients based on self-selected goals. A 5-point Likert scale was developed by Kiresuk and Sherman (Kiresuk & Sherman, 1968). The following scores apply: − 2, much worse than expected; -1, somewhat worse than expected; 0, expected goal; + 1, somewhat more than expected, and + 2, much more than expected. The GAS proved applicable to patients with acquired brain lesions (Bouwens et al., 2009). In pediatric rehabilitation, the GAS has been shown to be reliable, as reported by a review on the validity, reliability and sensitivity of goal setting (Hurn et al., 2006).

5 PARTICIPANTS

5.1. Sampling and participant recruitment

Stratified sampling will be used in this clinical investigation, with eight strata representing the different neurological diseases, as well as patients post stroke in the subacute and chronic phases who are severely and mild to moderately affected, respectively. This strategy will be chosen based on the primarily quantitative nature of this study.

All patients with a first-ever ischemic or hemorrhagic stroke, multiple sclerosis (MS), Parkinson's Disease (PD), a spastic para- or tetraplegia caused by any type of spinal cord lesion, mild to medium grade hereditary ataxia, acute or chronic inflammatory demyelinating polyneuropathy (e.g., GBS, CIDP) or a motoneuron disease (e.g., ALS) treated at the Reha Zentrum Münster during the recruitment period will be checked by the medical staff with regard to their suitability for participation in the clinical investigation. All patients who meet the inclusion criteria of this study and do not meet any of the exclusion criteria will be informed verbally and in writing about the study by the PI or investigator. After determining the general eligibility (inclusion criteria) and the consent of the patient i.e., their interest in participating in the study, a screening will be carried out. Using predefined criteria for intact cognitive functioning, with a cut-off score ≥24/30 points on the Mini Mental State Examination (MMSE) (Folstein et al., 1975), patients with moderate to severe cognitive impairment will be excluded. Furthermore, patients without walking impairment as defined by a neurological exam and an FAC score of 5 will be excluded. In the absence of these exclusion criteria, the study inclusion described below will take place.

After having provided the patient with the patient information sheet, an appropriate period of reflection for the patient, the opportunity for queries and answers and the patient's consent to participate, the informed consent form will then be dated and signed by both the patient and the doctor. The investigator will note the participation on a specific patient identification list. It will be used to later identify the patient and contain the identification number (ID), the full name of the patient, the date of birth and the date of admission to the clinical investigation. The patient identification list will remain at the trial centre after the clinical investigation has been completed.

5.2 Recording adherence to the intervention

For the intervention phase i.e., from the time of baseline testing until post-intervention testing (four weeks), a structured log will be completed by the treating physiotherapist for recording the actual performance of the planned RAGT intervention sessions or their non-performance, together with reasons.

5.3 Sample size

This clinical investigation will be a prospective comparative multiple case study. A comparative multiple case study design with mainly quantitative data but also qualitative data analyses and a cross-case analysis will be used. Mixing methods is a form of triangulation in research seen as mitigating the weaknesses found in single methods (Heale & Forbes, 2013). Triangulation in research is the use of more than one approach to researching a question. The objective is to increase confidence in the findings through the confirmation of a proposition using two or more independent measures (Noble & Heale, 2019). The combination of findings from two or more rigorous approaches provides a more comprehensive picture of the results than either approach could do alone (Noble & Heale, 2019). A multiple case-oriented research strategy will be adopted to acquire knowledge about the outcomes from RAGT within the 8 different patient (disease) groups, learn about similarities and differences between individual cases and neurological disorders within study intervention periods and emerging patterns (Ababacar et al., 2020; Nordbeck, 2013).

The primary outcome of this study will be the feasibility of the methods and of a larger randomised controlled trial. Based on the study design (Yin, 2014) and primary outcome, no formal sample size calculation has been carried out. Based on the different prevalence rates of the diseases investigated in this clinical investigation, 5 patients with severe stroke in the subacute phase will be included in this study, 5 patients with mild to moderate stroke in the chronic stage, 5 patients with MS, 5 patients with PD, 2 patients with spastic para- or tetraplegia caused by any type of spinal cord lesion, 2 patients with mild to medium grade hereditary ataxia, 2 patients with acute or chronic inflammatory demyelinating polyneuropathy (e.g., GBS, CIDP) and 2 patients with motoneuron disease (e.g., ALS), resulting in a total sample size of 28. Based on the study design, methods and objectives, no further patients will be included to account for attrition, which is expected at 10%.

5.4 Inclusion criteria

Potential study participants must meet all listed inclusion criteria in order to be eligible for participating in this clinical investigation. Patients will be recruited into this study if they have been diagnosed with a first-ever ischemic or hemorrhagic stroke, clinically stable phase of multiple sclerosis (MS), Parkinson's Disease (PD), a spastic para- or tetraplegia caused by any type of spinal cord lesion, mild to medium grade hereditary ataxia, acute or chronic inflammatory demyelinating polyneuropathy (e.g., GBS, CIDP) or a motoneuron disease (e.g., ALS). All patients must be in a clinically stable phase of their disease. Diagnoses will be confirmed using current criteria such as the Guideline of the German Society of Neurology (2017) (Hennerici & Kern, 2017) for stroke, the revised McDonald criteria valid at the time of diagnosis (Polman et al., 2011; Thompson et al., 2018) with MS, the UK Brain Bank criteria (Hughes et al., 1992) with idiopathic PD, Guideline of the German Society of Neurology (DGN 2012) or current NICE Guideline (*Spinal injury: assessment and initial management. Spinal*

injury assessment: assessment and imaging for spinal injury. NICE Guideline NG41, 2016), valid at the time of diagnosis, with spinal cord injury (SCI), the Ataxia Medical Guidelines 2016 or earlier versions, developed by Ataxia UK, London (de Silva et al., 2019) with hereditary ataxias, the diagnostic criteria originally developed by the National Institute of Neurological Disorders and Stroke (NINDS) ("Criteria for diagnosis of Guillain-Barré syndrome," 1978) in its revised version (Asbury & Cornblath, 1990; Hadden et al., 1998; Uncini et al., 2010) with GBS, the diagnostic criteria of the European Federation of Neurological Societies/Peripheral Nerve Society Guideline on the management of chronic inflammatory demyelinating polyradiculoneuropathy- First Revision ("European Federation of Neurological Societies/Peripheral Nerve Society Guideline on management of chronic inflammatory demyelinating polyradiculoneuropathy: report of a joint task force of the European Federation of Neurological Societies and the Peripheral Nerve Society--First Revision," 2010) or earlier versions with chronic inflammatory demyelinating polyneuropathy (CIDP) and the diagnostic criteria for ALS from the World Federation of Neurology as the El Escorial Criteria (Brooks, 1994), in their revised version as the Airlie House criteria (or El Escorial revised) (Brooks et al., 2000) or Awaji criteria (de Carvalho et al., 2008), or the recently developed Gold Coast criteria (Johnsen, 2020). There are no formal criteria for progressive muscular atrophy (PMA) and primary lateral sclerosis (PLS).

Patients with subacute, severe stroke who are non-ambulatory are eligible if they can be verticalised for at least 1 hour twice a day (which is a requirement for inpatient rehabilitation at the Reha Zentrum Münster). Patients with motor neuron disease in the early, active stage of the disease will be included and patients with mild to moderate hereditary ataxia. Patients will only be included if the are able to participate in this clinical investigation over its complete duration, which will be evaluated by the study PI. Further inclusion criteria will be impairment in walking according to the neurological exam assessed by a neurologist and Functional Ambulation Categories (FAC) of 0-4, intact cognitive function as defined by a score of ≥24/30 points on the Mini Mental State Examination (MMSE) (Folstein et al., 1975).

Adult patients aged between 18 and 99 of female, male and diverse genders, of any ethnicity and German speaking verbally and written language will be included. Patients will be included if they provide a signed and dated informed consent and are willing to comply with all clinical investigation procedures.

5.5 Exclusion criteria

Patients will be excluded from this clinical investigation if they meet any of the criteria listed as follows.

- Concomitant disease (such as malignant disease, other severe neurological, orthopaedic or psychiatric diseases, cardiac contraindications)
- Acute, pronounced pain symptoms despite conventional pain therapy

- joint contractures, joint arthrodesis or severe spasticity (stiff/immobile joint) in the area of the lower extremity, extremely disproportionate growth of the legs and/or spinal column
- Body weight of less than 15 kg or more than 180 kg
- Body height of less than 100 cm or more than 200 cm
- Insufficient compliance e.g., patients with a serious mental illness or severe neurosis
- Significant reduction in bone density (osteopenia or osteoporosis), increased risk of bone fracture
- Osseous or joint instability (non-consolidated fractures, osteogenesis imperfecta, unstable spinal column, pseudoarthrosis, hip, knee or ankle joint instability)
- Cardiac contraindications
- Severe vascular diseases of the lower limbs
- High-grade ataxia
- Skin lesions in areas which get in contact with the device or the harness system (decubitus); infections, skin ulcers, late effects of previous injuries, especially of the lower extremity
- States of health preventing active rehabilitation (e.g., respiratory diseases, orthopaedic diseases, cognitive impairments restricting communication, aphasia, neuropsychological disorders, infections or inflammatory diseases, osteomyelitis)
- Severe apraxia
- Severe osteoarthritis in the area of the joints of the lower extremity
- Risk of autonomic dysreflexia (AD) in patients with spinal cord injury (SCI) with neurological levels of TH6 or cranial
- Reduced compliance
- Uncooperative or (self)aggressive behavior
- Cardiac diseases, e.g., heart failure and thoracotomy, uncontrolled orthostatic hypotension or other circulatory problems, circulatory disorders of the lower limbs
- Recent joint injury or endoprosthetics (e.g., total endoprosthetics, knee or hip joint replacement, reconstruction of the cruciate ligament or meniscus) with contraindicated ranges of movement or load limits
- Consolidated fractures in the area of the lower extremity within a period of 6 months after injury
- Insufficiently treated epilepsy
- Recently occurred seizures or increased risk of seizures
- Mechanical ventilation
- Lack of head control
- Long-term infusions (e.g., Baclofen pump, intrathecal pumps, PEG tube …) or
- stimulators (e.g., pacemakers, nerve stimulators)
- Enterostomy/ostomy

- Sensory impairments of the lower extremities and the torso, especially reduced sensation of pain
- Pregnancy as assessed by a pregnancy test in women of childbearing age before enrolment into the study
- Any changes or adjustments to medication that has an influence on the participant's walking ability/performance will lead to the exclusion of the patient.

5.6 Timeline

The timeline of the clinical investigation is described as follows:

Study-specific:

Recruitment duration: 15 months
1.2.2022 Planned start: First Patient First Visit (FPFV)
31.3.2023, 2022 Last Patient First Visit (LPFV)/Last Subject In (LSI)
30.4.2023 Planned end: Last Patient Last Visit (LPLV)/Last Subject Out (LSO)

Patient-specific:

Active intervention duration: 4 weeks (baseline assessment, 4-week intervention, post-intervention assessment)
Figure 5 presents a flowchart of the patient-specific study process.

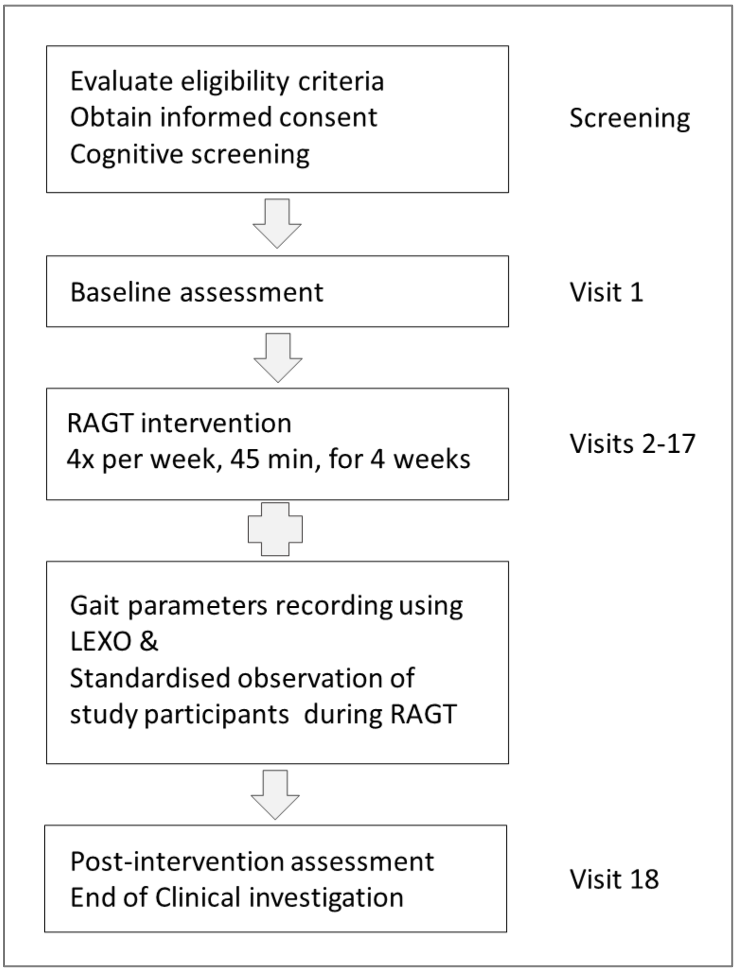

Figure 5: Flow diagram of the patient-specific study process

6 INVESTIGATION PREPARATION PROCEDURES

6.1. Investigator selection

Investigator selection criteria

The PI and the investigator are responsible for implementing, monitoring, and conducting all procedures related to this clinical investigation in accordance with the provisions of the clinical investigation protocol. Furthermore, the PI and investigator are responsible for making relevant decisions on the clinical investigation and medical treatment to ensure data integrity and the rights, safety, and well-being of the patients participating in the clinical investigation.

A further investigator may be included in the clinical investigation if he*she meets the following requirements:

- appropriately qualified and experienced in the field of neurology and/or neurorehabilitation
- experience in conducting clinical investigations or appropriate training,

- availability of sufficient time for following-up on the clinical investigation,
- willingness to comply with the clinical investigation protocol,
- willingness to undergo investigation-specific training,
- Familiarity with the requirements of ISO 14155 and if otherwise, willingness to undergo appropriate training,
- Willingness to sign and comply with the clinical investigation agreement,
- Willingness to undergo auditing by sponsors or regulatory authorities.

Clinical Investigation Agreement

A Research Agreement has been signed between the Reha Zentrum Münster Betriebs GmbH (RZM) and Tyromotion GmbH.

The agreement states that the purpose of this Collaboration is to systematically collect, analyse, and publish protocols and data on the clinical use of the LEXO® robotic gait trainer in the inpatient setting. A prospective comparative multiple case study will be used to investigate a variety of patients with different main diagnosis.

TYROMOTION primarily aims to collect data on effectiveness (performance) and safety (adverse events, side-effects, and drop-outs) on the LEXO® robotic gait trainer, but also on usability and feasibility, and the quality of the product.

A Purchase Order has been signed between the two parties, where Tyromotion will provide a LEXO® robotic gait trainer for the Reha Zentrum Münster that will conduct the planned clinical investigation followed by a larger randomised controlled trial.

The people involved in decision-marking about this funding have no influence on the planning, conduct and publication of the clinical investigation. None of the investigators or other members of the study team receive a financial compensation for their contributions in this clinical investigation.

6.2. Approval of Ethics Committee

The responsible ethics committee (EC) for this clinical investigation is the Ethics Committee of the Medical University of Innsbruck. The clinical investigation shall not begin until approval from the EC has been obtained. The EC will be informed of any subsequent changes to this clinical investigation (amendment).

6.3. Handling of amendments to the Clinical Investigation Plan

To ensure a consistent study procedure in the interests of flawless data evaluation, it is not intended for there to be changes from the clinical investigation conditions planned and included in the protocol.

In exceptional cases, changes to trial conditions are possible, however. These only take place following mutual agreement between the PI, investigator and the sponsor of the study. Any change to the clinical investigation procedures set out in the protocol must be made in

writing, specifying the reasons in question, and signed by all study investigators and the sponsor. The changes will then be part of the protocol. A distinction is made between essential and non-essential amendments. Substantial amendments are subject to approval and must be submitted to the competent EC. In case of non-essential amendments (for example purely formal changes) a written notification is sufficient. In the case of significant changes that have a direct impact on the safety of study participants or other significant changes, the consent of the responsible EC and/or authorities and the patient must be obtained for protocol amendments.

The investigator has the option to stop the study overall in emergency situations between the application for a protocol amendment and such amendments being rejected or coming into effect. This is mandatory if a protocol amendment or a change in the state of knowledge for study-specific examinations occurs that leads to a potential reassessment of the risk/benefit calculation. In this case, the study will only be continued once an updated opinion has been received from the EC.

6.4. Training requirements

The clinical investigation team will undergo different trainings prior to commencement of the study. All new study team members will undergo the training outlined here.

This first and foremost includes the responsibilities of the investigator as outlined in Chapter 6.1 and ISO 14155 requirements. The PI and investigator will inform patients and obtain their informed consent

The clinical investigation team (physiotherapists) that will conduct the RAGT intervention are trained on the use of the LEXO® device by authorised personnel from Tyromotion. The training will consist of three phases: (1) provision of the training documents for the LEXO® to the study team, followed by an evaluation; (2) a two-day training consisting of a theoretical and a practical part and (3) a refresher training day a few weeks after the first training. In addition, the LEXO® is used also in the clinical routine at the Reha Zentrum Münster, which is why the physiotherapy team is familiar with the procedures.

The clinical investigation team (physiotherapists) that will perform the motor assessments in this study will undergo training from Dr. Barbara Seebacher who is an experienced neurological physiotherapist and senior researcher to ensure consistency.

6.5. Clinical investigation materials and equipment

Except the LEXO®, no further investigation specific materials or equipment will be provided by the sponsor.

6.6. Investigational device / Device accountability

The Reha Zentrum Münster will be supplied with the LEXO® well before the start of the Clinical Investigation and be installed appropriately by authorised personnel from Tyromotion. The delivery of the device will be made as outlined in a formal checklist (described below) via a semitrailer, forklift truck and lift truck and using further appropriate measures for guaranteeing a safe delivery. A suitable therapy room has been chosen for the device and checked in terms of all technical requirements, delivery details, load-bearing capacity of the ground, infrastructure, patient entry side and maintenance. A formal checklist has been completed by the technical team from Reha Zentrum Münster including a structural engineer and returned to Tyromotion. According to the Clinical Investigation Agreement, the LEXO® will remain at the study site after the end of the clinical investigation as a larger follow-up randomised controlled trial is proposed. Following both clinical investigations in a high-quality manner and publication of the study results, the purchase of the LEXO® will be complete. Details of the clinical device to allow a clear identification are described in Chapter 3.

7. STUDY PROCEDURES

7.1. Assessment and treatment schedule

The clinical investigation will comprise a screening, a baseline examination (T1), 16 intervention units over 4 weeks, and a post-intervention examination (T2).

The duration and number of the examinations (Table 1) will be used in addition to the patient's regular inpatient rehabilitation treatment. The intervention itself and some assessments will be performed as part of the clinical routine (for details, see Chapter 4). The PI, investigator and psychologists will perform any disease and cognition related examinations. All therapists conducting the movement-related assessments will be trained, and they will therefore work based on the same principles. Furthermore, the physiotherapists at the study site are using some the assessments on a daily basis (e.g., 10MWT, 6MWT), but for ensuring consistency, their training will be refreshed. The clinical investigation team (physiotherapists) that will conduct the RAGT intervention are trained on the use of the LEXO® device by authorised personnel from Tyromotion. In addition, the LEXO® is used also in the clinical routine at the Reha Zentrum Münster, which is why the physiotherapy team is familiar with the procedures. The clinical investigation team (physiotherapists) that will perform the motor assessments in this study will undergo training from Dr. Barbara Seebacher who is an experienced neurological physiotherapist and senior researcher to ensure consistency. In Table 8, the study procedures and schedule are presented; the table includes both the procedures which are performed ***within the clinical routine (highlighted green)*** and ***in addition to the clinical routine (highlighted yellow).***

Table 8: Treatment and assessment regimen of a (screened, potential*) study participant

**Procedure**	**Dosage**	**Time**	**Duration within clinical routine**	**Duration in addition to clinical routine**
Screening*	1x	After checking for eligibility by the PI/investigator and the patient's consent	10-15 min	--
Study inclusion				
Baseline examination (T1)	1x	Day 0-1 after study inclusion	10 min	70-90 min
Movement-related goal setting	1x	Day 0-1 after study inclusion	--	5 min
Intervention	16x	Day 1-2 after inclusion<br>4x per week<br>(Total 4 weeks)	45 min	--
Semi-structured observation of the intervention	2x	Week 1<br>Week 4	--	No additional effort for patient
Post-intervention examination (T2)	1x	After 4 weeks	10 min	70-90 min

*If the patient meets one or more exclusion criteria after screening, the patient cannot be enrolled in the study.

7.2. Intervention

The intervention will be a part of the routine care within inpatient rehabilitation at the Reha Zentrum Münster. The LEXO® is intended for the robot-assisted gait rehabilitation of patients with a limited ability to walk due to neurological damage to the central and peripheral nervous systems as well as due to orthopedic, geriatric and pediatric symptoms. Depending on national differences, the LEXO® is typically used in a physiotherapy environment in order to support, enhance, and intensify therapy. The repetitive movements of the lower limbs, and consequently of the gait pattern, generate intrinsic and extrinsic stimuli which favor the reorganization of the brain. Repeated, active practice and training encourages the neuronal plasticity and thus changes (adjustments) of synapses, nerve cells or even entire brain areas to recover lost movements. The LEXO® is an end-point mechatronic therapy system that allows static and dynamic body weight support, electrically driven pelvic guidance and computer-based setting of therapy parameters. The gait training addresses balance, locomotion, musculoskeletal integrity and neurocognitive control.

With regard to adaptation criteria of the RAGT, first, the different neurological disease groups will be considered, with different symptom complexes involving distinct central and peripheral nervous system (CNS, PNS) sensorimotor system areas and pathways (upper and lower motor neurons including both the cerebrum and spinal cord, extra pyramidal motor system, peripheral nervous system and cerebellum). Main motor symptoms include spasticity, rigidity, weakness, ataxia, brady- and hypokinesia and tremors and sensory symptoms include a loss in proprioception and hypesthesia among others. Second, the training will be adapted according to the individual performance level of the participants (see Table 9). RAGT will be performed within the inpatient rehabilitation routine setting for 45 minutes, 4 times a week, for 4 weeks, and will be supervised by the responsible physiotherapists. The RAGT will be integrated into patients' inpatient rehabilitation and be planned at similar daytimes to reduce patient burden and facilitate comparability. During the 45 min training period, participants will be allowed to take a break at any time, if this is desired or necessary. Based on the findings of the previous literature and with advances in walking performance, the training parameters for the different patient groups have been selected and will be progressed in difficulty (Capecci et al., 2019; Freivogel et al., 2008; Luca et al., 2013; Pohl et al., 2007; Wu et al., 2014). Explicit feedback on the walking performance will be given by the supervising therapist and implicit feedback will be provided as augmented performance feedback via a computer screen in front of the patient, based on the different measurements of parameters by the robot. For example, visual implicit feedback will be given on distance, duration, and number and length of steps.

Table 9: RAGT parameters and their adjustment for achieving easy to hard training levels

**Parameter**	**Easy**	**Medium**	**Hard**
Body weight support	≤100% body weight support	≤30% body weight support	0-5% body weight support
Training mode and duration	≤100% passive walking mode	≤50% passive walking mode, ≤49% assistive walking mode	≥50% assistive walking mode
Step length (27-61cm)	27-39 cm	40-50 cm	51-61 cm
Max. training velocity	≤1,4 km/h	1,5-2,0 km/h	≥2,0 km/h

Walking distance	≤150 steps	151-500 steps	≥500 steps
Break time	As much as needed		
	Reduction of the RAGT time if required	Up to 20% of the training time (9/45 Min); either standing or reducing walking speed	0-5% of the training time (2,25/45 Min); reducing walking speed, if necessary

All participants will be secured with a safety harness that is able to dynamically support body weight. The harness is positioned over the LEXO® and can also be used to lift non-ambulatory participants into the training position. For participants out of Cases 1 (early, severe stroke) and severely affected participants out of Cases 7 and 8 (acute/chronic inflammatory demyelinating polyneuropathy; motor neuron disease) the initial weight support will be adjusted individually (estimated 10-40% of the total body weight) and will be reduced as rapidly as possible. The physiotherapist will assist the paretic knee control, but with further improvement participants will practice independently. If possible, participants out of Cases 2 to 8 (chronic, mild to moderate stroke; multiple sclerosis; Parkinson disease; spastic para-/tetraplegia; hereditary ataxia; acute/chronic inflammatory demyelinating polyneuropathy; motor neuron disease) will perform the RAGT without body weight support. The step length will be adjusted individually to achieve a comfortable gait velocity for all participants. The first session will start with a walking speed of 1.5 km/h, which will be increased progressively to 2.2-2.5 km/h. The intensity of the RAGT training sessions, including the amount of break-time during training, will be adapted to the individual level of impairment, presence of weakness, pain or fatigue among others.

7.3 Semi-structured observation of the intervention

A semi-structured observation of the intervention will be conducted in week 1 and week 4 by a trained physiotherapist of the assessment team. The duration of the observation will be 50 min i.e., during the total RAGT duration and will not involve any additional effort of the study patient/participant. The structured part of the observation will involve the set-up and closure of the patient, the body position of the patient with relation to his or her trunk, lower and upper limbs, the reported percentage of the body weight support, the effort and potential fatigue or pain of the patient (that will be cared for as with any other rehabilitation strategy within a routine rehabilitation setting), the mimic and complexion/face colour of the patient, verbal and non-verbal utterings of the patient, verbal and non-verbal utterings of the therapist, the motivation of the patient, the communication between the patient and the therapist, the general atmosphere in the room, the overall environment and the overall

progression of the RAGT from week 1 to 4. In addition to the structured observation, an unstructured observation will be used to collect any other relevant information as to the performance of the patient and the conduct of the intervention.

7.4 Concomitant treatment

All study participants will receive their usual care including medical exams, rehabilitation assessments and goal setting, and medical treatment including medication. Study participants will undergo their usual inpatient rehabilitation comprising multidisciplinary rehabilitation (medical treatment, physiotherapy, occupational therapy, speech and language therapy, psychology counselling, dietetics etc.).

It is not planned to include patients who need relevant changes to their medication, but the medication will be adjusted and documented if the study PI considers this a requirement. Any changes or adjustments to medication that has an influence on the participant's walking ability/performance however will lead to the exclusion of the patient.

7.5 Participant drop-out

Study participants are free to withdraw their consent from the clinical investigation participation at any time without having to give a reason and without any consequences for their future treatment. In addition, patients may be withdrawn from the study by the PI if their health is in jeopardy.

The following points or events will lead to the patient's exclusion from the study if they occur after their initial admission in the study (dropout in terms of the biometric analysis):

- No questionnaire scores, assessment scores or LEXO® measures of interpretable quality.
- Withdrawal of consent to study participation by the study participant during integration into the study.
- An MS relapse or other disease exacerbation for any of the included diseases during the intervention period.
- Any changes or adjustments to medication that has an influence on the participant's walking ability/performance.

If the patient's health is jeopardised after the study intervention has started as a result of an unexpected deterioration in their state of health or general condition and/or mental/physical training is absolutely contraindicated, participation in the study will be ended immediately and a general physician or specialist will be consulted.

The reason for the patient's departure from the study will be documented in the CRF. The study is to be continued with the next identification number, with no replacement of study participants allowed. All patients leaving the study prematurely will be asked to undergo a

final examination with regard to their safety and as part of post-intervention testing at the next possible opportunity. The results of these tests will be documented in the CRF.

7.6 Early termination or suspension of the clinical investigation

The sponsor is authorised to terminate the study early or to suspend the study because of relevant medical or administrative causes. The criteria and reasons for ending the study are documented in detail. Patients who were still receiving treatment at the time of the termination of the trial will be asked to undergo a final examination as part of post-intervention testing at the next possible opportunity. The results of these tests will be documented in the CRF. Should an investigator have ethical concerns regarding the continuation of the study, these must be communicated to the sponsor immediately.

The sponsor is authorised to end the clinical trial prematurely if

- There is unequivocal evidence of treatment benefit or harm
- The patient recruitment rate cannot be reached,
- Serious, unresolvable problems arise with the quality of the data collected,
- Unforeseeable circumstances arise in the trial site, which means that the continuation of the clinical investigation is not permissible,
- Unexpected unacceptable side effects occur from the RAGT intervention,
- Unreasonable risks arise (decision after another risk/benefit assessment),
- New scientific knowledge acquired during the study no longer permits the continuation of the study (e.g., reports of unsuspected and unacceptable treatment side effects),
- No trends emerge as for the benefit of the RAGT intervention after a reasonable number of patients have been investigated and the results clinically interpreted,
- There is a lack of compliance in a large number of patients,
- Withdrawal of the LEXO® robotic gait trainer from the market

The PI can decide whether to terminate the study in consultation with the sponsor.

Should an investigator have ethical concerns regarding the continuation of the study, these must be communicated to the sponsor immediately.

7.7 Medical care after exit from the clinical investigation

In principle, the intervention of this clinical investigation is part of the clinical rehabilitation routine i.e., the usual treatment of the patients and not an add-on treatment. After patients have terminated their participation in this study, their treatment will be continued according to their treatment scheme in the same way as during the study. This includes their medication and usual physiotherapy or any other medical treatment or assessment.

7.8 End and closure of the clinical investigation

The end of the clinical investigation refers to the point of final data capture i.e., the date of last patient last visit (LPLV). This is followed by the study analyses and drafting a study report. The following end or closure of study notifications and further actions are required:

- End of study notification of the sponsor Tyromotion that the data collection has concluded as defined in the protocol.
- End of study notification of the responsible research ethics committee of by submitting a Declaration of the End of Study Form.
- Provision of the representative of the sponsor with a declaration of the end of study form.
- The end of study declaration and all related correspondence will be retained in the Trial Master File (TMF).
- Closure of study notification of the sponsor that the data analyses have been concluded as defined in the protocol.
- The PI will submit a summary report on the preliminary effectiveness (performance) and safety (adverse events, side-effects, and drop-outs) of the LEXO® robotic gait trainer, but also on usability and feasibility to the sponsor.
- Notification of the sponsor of all (planned) publications resulting from the research.

8. STATISTICAL DESIGN AND ANALYSIS

8.1. General aspects of the analysis

The analysis of this comparative multiple case studies uses mixed methods, integrating quantitative and qualitative data. These data will be analysed together with the intention of gaining an in-depth understanding of the cases. In addition, the use of mixed methods will purposefully be adopted to generate causal propositions. A comparison of the walking, balance, and other outcomes within the cases of one stratum (disease group) and between the cases (disease groups) will be at the heart of the comparative approach.

A single case can also be the source of new insights about critical success factors. For example, it may become apparent that RAGT has improved walking in one patient but failed in others. Therefore, such a case may be selected and the attributes and features that may be responsible for the differences explored in more depth. In addition, triangulation will be used in evaluation to check answers to descriptive questions about the way things are, for example, by confirming semi-structured observations of the intervention with direct measures from LEXO® or standardised subjective observations or motor test performances.

8.2. Planned statistical analysis

IBM SPSS Software, release 27.0 (IBM Corporation, Armonk, NY, USA) and GraphPad Prism 9, San Diego, California, will be used for the data analyses. Statistical significance will be defined as a two-tailed p-value <0.05. The percentage of missing data will be recorded. Using Little's test of missing completely at random (MCAR) (Little, 1988) the data will be checked, signified by a p-value >0.05.

Descriptive statistics will be used for the baseline demographic variables, primary and secondary endpoints. Continuous data will be checked for normal distribution using the Shapiro Wilk Test, Q-Q plots and histograms. Raw count (absolute and relative frequencies, N (%) will be presented for count data (N females and males, N falls, most affected body part, and N adverse events if any, adherence (N of training sessions), recruitment, retention and adherence rates) and nominal data (gender)). Medians (range; 25th and 75th percentiles) will be reported for ordinal data (MMSE, NIHSS, EDSS, UPDRS, H&Y, ASIA Scale, SARA, MAS, ONLS, ALSFRS-R, FAC, TCT, FES-I, EQ-5D-5L, BDI-II, FSS, GAS, Smiley Likert Scale), mean (standard deviation (SD)) will be reported for continuous data (age, years of education, disease duration, 10MWT, FGA, 6MWT, TUG, FSST and MFRT).

With respect to the primary endpoint of feasibility, the recruitment rate (%) will be estimated by dividing the number of consenting patients by the N of eligible patients, multiplied by 100. The retention rate determined by dividing the N of patients who completed the study by the N of the total sample, times 100. Adherence rates will be calculated by dividing the N of RAGT sessions (16x) performed by the patients, divided by the N of the scheduled RAGT training sessions over the 4 week study period, times 100 (Osterberg & Blaschke, 2005). The eligibility, recruitment and adherence rates will be calculated with their 95% CI according to the Wilson 'score' method cited by Newcombe (Newcombe, 1998); when the proportion is close to 0 or 1, a Poisson approximation as described by Brown and colleagues will be used (Brown et al., 2001). Furthermore, the number of (serious) adverse events and side effects will be reported.

The first phase of the analysis will involve a descriptive analysis for all disease groups using the mean (SD) or median (range, 25th and 75th percentiles). The second phase of the analysis will include inferential statistics using a pre-post analysis of the data from the total sample (one group). For continuous data, a paired t-test will be performed and for ordinal data, a Wilcoxon Signed Rank test.

8.3 Planned qualitative data analysis

(Jotted) field notes from the semi-structured observation will be expanded in writing and then entered into Excel files. A Thematic Analysis (TA) (Braun & Clarke, 2006, 2021) will be performed. TA is a method for systematically identifying, organising, and offering insight into

patterns of meaning (themes) across a dataset. Through focusing on meaning across a dataset, TA allows to observe and make sense of collective or shared meanings and experiences. TA is a flexible method that allows focusing on the data in numerous different ways i.e., both on analysing meaning across the entire dataset or examine one particular aspect of a phenomenon in depth. The TA analysis will be performed in 6 phases as suggested by Braun & Clarke (Braun & Clarke, 2006, 2021).

Phase 1: Familiarising oneself with the data

This phase involves immersing oneself in the data by reading and re-reading textual data. Making notes on the data as they are read is part of this phase. Reading data as data means reading the words actively, analytically and critically, and starting to think about what the data mean. The aim of this phase is to become intimately familiar with the dataset's content, and to begin to notice content that might be relevant to your research question.

Phase 2: Generating initial codes

The systematic analysis of the data through coding is started in phase 2. Codes are the building blocks of analysis as they identify and provide a label for a feature of the data that is potentially relevant to the research question. Coding can be done at the semantic (descriptive) or the latent (interpretative) level of meaning. Codes are succinct and work as shorthand for relevant aspects of the data. Codes will almost always be a mix of the descriptive and interpretative. Coding will be done in large or small chunks; some chunks won't be coded at all. Coding requires another thorough read of every data item. Each data item will be coded in its entirety before coding another. Codes will be named and the text around it marked using an inclusive, thorough and systematic approach.

Phase 3: Searching for themes

In this phase, themes are created from the dataset. A theme "captures something important about the data in relation to the research question, and represents some level of patterned response or meaning within the data set" (Braun & Clarke, 2006) (p. 82). This phase will involve reviewing the coded data to identify areas of similarity and overlap between codes. The basic process of generating themes and subthemes (the subcomponents of a theme) will involve collapsing or clustering codes that seem to share some unifying feature, so that they reflect and describe a coherent and meaningful pattern in the data. Certain concepts or issues may cut across themes and provide a unifying framework for telling a coherent story about what is going on in the data, overall. Another important element of this stage is starting to explore the relationship between themes, and to consider how themes will work together in telling an overall story about the data. Good themes are distinctive and, to some extent, stand-alone, but also need to work together as a whole. According to the recommendation from Clarke and Braun, a maximum of 6 to 7 themes will be generated. A

thematic map will be generated to outline the candidate themes, and all the data extracts relevant to each theme will be collated.

Phase 4: Reviewing potential themes

Phase 4 will involve a recursive process whereby the initially developed themes will be reviewed in relation to the coded data and entire dataset. The themes will be checked against the collated extracts of data (1. Is this a theme?) and explored whether the theme 'works' in relation to the data (2. Does this theme tell me something useful about the dataset, in relation with my research question?). If necessary, some codes will be discarded or relocated under another theme. Alternatively, the boundaries of a theme will be redrawn, so that it more meaningfully captures the relevant data (3. What are the boundaries of this theme?). It will further be checked whether there are sufficient meaningful data to support a theme (4.) and whether a theme contains coherent data (5.). If a theme does not work, it will be discarded altogether, split into two or more themes, or collapsed with another theme, and the process started again.

Phase 5: Defining and naming themes

In phase 5, it will be clearly stated what is unique and specific about each theme. Themes should ideally have a singular focus, scope and purpose (1); be related but don't overlap, so they aren't repetitive, although they may build on previous themes (2); and directly address the research question (3). This phase involves refining the analysis and starting the write-up. Extracts will be selected to quote and analyse, which provide the structure for the analysis. A descriptive TA will be used, where data tend to be used in illustrative ways, and conceptual/interpretative, where extracts tend to be analysed in more detail. Informative, concise, and catchy names will be used for naming the themes.

Phase 6: Producing the report

The purpose of the report is to provide a compelling 'story' about the data, based on the analysis.

8.1. Handling of missing data

The percentage of missing data will be recorded. Using Little's test of missing completely at random (MCAR) (Little, 1988) the type of missing data (MCAR, missing at random (MAR), missing not at random (MNAR)) will be checked, signified by a p-value >0.05.

8.2. Strengths and Limitations of the Clinical Investigation

Strengths

- Due to the different diagnoses treated at the Reha Zentrum Münster, no other medical facility is needed to recruit patients for this prospective comparative multiple case study.
- Great professional and scientific expertise of the study team
- Many different diagnoses can be investigated in a prospective comparative multiple case study.
- A case-oriented research strategy will be adopted to acquire knowledge about outcomes within the 8 disease groups, learn about similarities and differences between cases and neurological disorders within the individual intervention periods and emerging patterns, and for theory development (Ababacar et al., 2020; Nordbeck, 2013). However, the synthesis across cases will extend beyond the comparison of similarities and differences to generating information on the reasons and explanations as the findings from the RAGT intervention (Goodrick, 2014). In other words, except some degree of comparison that will be fundamental to the comparative multiple case study design, the differentiating feature of this study type is the emphasis on examining causality i.e., the extent to which the RAGT intervention caused the outcomes (Goodrick, 2014).
- In practice, this comparative multi case study will involve the analysis and synthesis of the similarities, differences and patterns across multiple cases, with the shared common goal of gaining knowledge about the feasibility, acceptability and preliminary effects of RAGT.
- Case selection will be performed using two different strategies: within disease groups, the ‘Most Similar Systems Design’ (MSSD) will be applied and between disease groups, the ‘Most Different Systems Design’ (MDSD) (Anckar, 2008; Przeworski & Teune, 1970) will be used, considering 8 neurological disease groups with symptom complexes involving distinct sensorimotor system parts of the central nervous system (upper and lower motor neurons including both the cerebrum and spinal cord, extra pyramidal motor system, peripheral nervous system, cerebellum and afferent pathways including their respective representations in the cortex). As for MSSD, it will mean to choose study patients that are similar in as many background characteristics as possible, but without systematically matching cases on all the relevant control variables. As for MDSD, the strategy will be to select patients that are as different as possible with respect to extraneous variables. The basic logic is that differences cannot explain similarities (Przeworski & Teune, 1970).
- The objectives, inclusion and exclusion criteria, endpoints and assessments and study procedures are clearly stated.
- Both a quantitative and qualitative data collection and analyses will be used.
- Mixing methods is a form of triangulation in research seen as mitigating the weaknesses found in single methods (Heale & Forbes, 2013). Triangulation in research is the use of more than one approach to researching a question. The

objective is to increase confidence in the findings through the confirmation of a proposition using two or more independent measures (Noble & Heale, 2019). The combination of findings from two or more rigorous approaches provides a more comprehensive picture of the results than either approach could do alone (Noble & Heale, 2019).

- The intervention of this study uses a standard RAGT protocol but is individualised and hence adapted to the different patient groups (Cases 1-8) and the individual performance level of the participants.
- Structured data documentation, management and evaluation will be used as outlined in this clinical investigation protocol
- Continuous monitoring for adverse events using a structured log and predefined evaluation criteria
- Comparative multiple case studies are useful for evaluating of feasibility, practicability, acceptability and preliminary results and for refining study procedures and therapy protocols before planning and commencing a large randomised controlled trial.

Limitations

- Low number of patients per diagnosis – due to low power, efficacy testing cannot be performed for the different disease groups but overall.
- Limited generalizability of results
- No control group
- No follow-up assessment

As outlined in Chapter 2.1, the purpose of this clinical investigation is to investigate the feasibility and acceptability and compare the preliminary effects of RAGT a mixed population with neurological diseases consisting of patients with stroke in the subacute and chronic phases and with severe and mild to moderate disability, MS, Parkinson's Disease, spastic para-or tetraplegia, mild to medium grade hereditary ataxia, acute or chronic inflammatory demyelinating polyneuropathy and motoneuron disease. A further purpose of this clinical investigation is to synthesise quantitative and qualitative results for examining causality i.e., the extent to which the RAGT intervention may have caused the outcomes.

The results from this study can be used for designing a full-scale randomised controlled trial. The study population and all study procedures have been specified a priori, the study team will strive to carry out all measures as planned and use a structured data documentation, management and evaluation as outlined in this clinical investigation protocol. The intervention of this study uses a standard RAGT protocol but is individualised and hence adapted to the different patient groups (Cases 1-8) and the individual performance level of the participants. Continuous monitoring for adverse events using a structured log and predefined evaluation criteria will be performed. Therefore, it is not expected that the limitations of this study negatively impact on the conduct of the clinical investigation, data quality and results.

9. SAFETY

9.2 Adverse event - definition

An adverse event (AE) is any unfavourable medical occurrence, unintended disease or injury or any unfavourable clinical sign that affects a patient during participation in this study and is not necessarily in a causal relationship with the study intervention. These may be diseases, signs and symptoms of disease that arise after the patient's inclusion in the study, or pre-existing diseases, signs and symptoms of disease that get worse after inclusion in the study. This excludes pre-existing diseases that do not get worse over the course of the study and adverse events that are associated with a concomitant medication. This definition includes events related to the study intervention and to the study procedures. According to the World Health Organization (2002), CIOMS (Council of International Organizations of Medical Sciences) and ICH (International Conference on Harmonisation), adverse events are medical occurrences temporally associated with the use of a medicinal product or intervention, but not necessarily causally related.

9.3 Adverse reaction and side effect - definitions

A side effect (SE) is any adverse event defined above that has a causal connection with the study intervention. Side effects are often expected, and often refer to symptoms that are less harmful than adverse reactions. An adverse reaction is 'A response to a drug [or intervention] which is noxious and unintended, and which occurs at doses normally used in man for the prophylaxis, diagnosis, or therapy of disease, or for the modifications of physiological function' (WHO 1972).

9.4 Serious incident - definition

A serious incident is any adverse event or any adverse reaction, which either

- Leads to death, or
- Is life-threatening, or
- Leads to lasting or serious disability or invalidity, or
- Makes inpatient treatment or an extension to inpatient treatment necessary (except for if the inpatient treatment was planned before participation in the study).

A distinction is made between a serious adverse event (SAE) or a serious adverse reaction (SAR), depending on whether it has a causal relationship with the study intervention.

9.5 Severity

The severity of incidents ((S)AE and (S)AR) are defined as follows:

- Mild – the intervention has to be interrupted due to the incident, but may be resumed on the same or the subsequent day. It is not necessary to consult a doctor for treatment in this case.

- Moderate – the intervention has to be interrupted due to the incident, but may be resumed on another day, but not immediately the subsequent day. It is not necessary to consult a doctor for treatment.

- Serious – the intervention must be stopped and cannot be continued due to the event (a drop-out according to Chapter 7.5)

9.6 Causality

Causality is defined as follows:

- Treatment-related – It is either known that the adverse event may be caused by the study intervention or there is a justified suspicion that the study intervention caused the adverse incident or is there is a temporal relationship between the study intervention and the occurrence of the adverse incident.

- Not treatment-related – there is no justified suspicion that the study intervention caused the adverse incident or is there is no temporal relationship between the study intervention and the occurrence of the adverse incident.

9.7 Assessment and documentation

As part of this study, all adverse events and adverse reactions are systematically recorded, evaluated and assessed together at the end of the study.

Assessment process

An authorized doctor must evaluate and confirm the possible association with the intervention, severity (mild, moderate, severe) as well as classification of the incident as serious or non-serious.

The incidents are classified based on the assessment scheme presented in Figure 6.

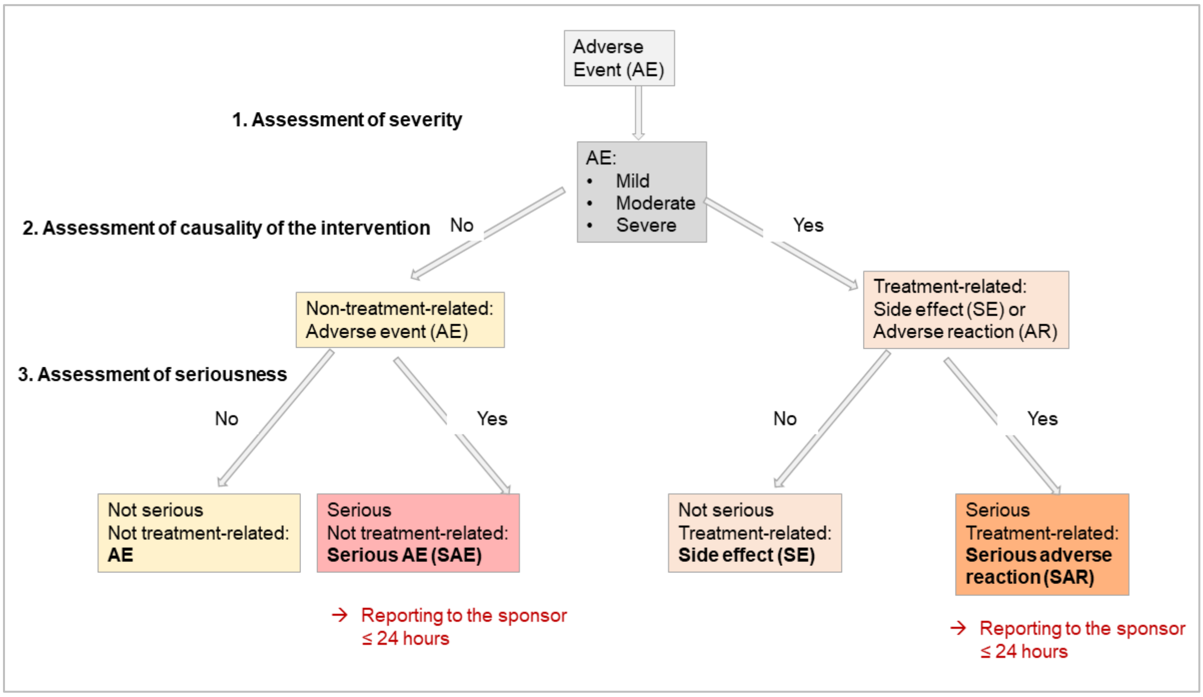

Figure 6: Assessment process for adverse events, side effects and adverse reactions

Documentation of adverse incidents (AE/AR)

During the intervention phase (Week 1 – Week 4), the occurrence of the aforementioned events will recorded by the responsible employee at the study site and assessed by a member of the medical team (investigator, PI).
All incidents and findings must be documented in the patient files and then in the AE log in the CRF.

The following information is necessary:

- Type of adverse event (signs, symptoms or disease)
- Start and end of occurrence
- Severity
- Causality of the intervention
- Differentiation (serious/non-serious)
- Measures concerning the intervention or actions to restore or improve the patient’s wellbeing
- Outcome of the event.

Documentation of serious incidents (SAE/SAR)

Serious incidents must also be entered into the form for reporting serious incidents in addition to documenting them in the AE log in the CRF.

When documenting serious incidents, the following points must be strictly observed:

- Each SAE/SAR must be reported in as much detail as possible.
- Pseudonymised findings (using the patient code) must be enclosed with the report

The investigator at the study site must check that the reporting form for serious incidents is complete and ensure that the information entered matches that in the AE log of the CRF and further data sources.

9.8 Reporting of serious incidents (SAE/SAR)

Investigator's notification obligations

All serious events that occur during the study must be reported immediately upon becoming aware of them (≤ 24 hours/1 working day) by sending the form for serious incidents to the study PI (email: christian.brenneis@reha-muenster.at).

Please note: Personal data (e.g., results) must be pseudonymised using the patient code before transmission (GDPR-compliant: personal, mail, analogue fax).

Sponsor's notification obligations

The sponsor must inform the Ethics Committee of the Medical University of Innsbruck of all serious incidents that arise over the course of the study in writing immediately upon becoming aware of them (≤ 24 hours/1 working day).

In the event of incidents that potentially lead to elevated risk, the ethics committee must be contacted again.

10. QUALITY CONTROL PROCEDURES

10.1. Data collection methods

Data to evaluate the endpoints will be collected exclusively with valid, reliable and responsive instruments, which are listed and described in Chapter 4.8.

10.2 Investigator Site File

An Investigator Site File (ISF) will be set up at the beginning of the study. It will collect all essential documents pertaining to the study, which in turn will allow for effective monitoring and supervision (auditing by the sponsor). The ISF will be set up in compliance with the ICH

Harmonised Guideline, Integrated Addendum to ICH E6(R1): Guideline for Good Clinical Practice E6(R2), current step 4 version (2016) and the protocol.

The ISF will be maintained at the study site in the PI's office and continuously updated by the PI and investigator or authorised study personnel. The ISF will contain copies of all relevant documents. The ISF will also include all site-specific essential documents e.g., the study protocol, the informed consent documentation, the Investigator's Brochure (IB) of the LEXO®, financial agreements, insurance policies, research ethics board review and approval, the pseudonymisation list, the case report file (CRF), assessment manuals and instructions, training documentation for the study team, patient screening log, signed informed consent forms, patient enrolment list, serious adverse events reports, and final study report. Documentation should be accurate, attributable, complete, legible, and filed in a timely manner. After completion of the clinical investigation, the ISF will be checked for completeness and archived together with the patient data for the duration defined in Chapter 10.3.

10.3 Storage of study documents, data storage & erasure

Records and documents that are connected with the study (e.g., informed consent forms, case report forms, other relevant documents) must be stored by the PI for a period of 15 years after the conclusion or termination of the clinical trial. The PI shall ensure that the documents concerning pseudonymisation are stored for a period of 15 years after the conclusion or termination of the clinical investigation. The study-related data and documents will be stored in secure premises and on secure servers. The patient forms and other original data must be stored for the longest period of time permitted for the institution. Access to the data and documents will be limited to the PI and investigator, and members of the study team as authorised by the PI. The documents will be destroyed after 15 years have passed.

10.4 Documentation

Source data and documents

All information from original documents and reports as well as their certified copies that are required to reconstruct and evaluate the study are considered source data. Source data will be contained in source documents.

The source documents in this study are:

e.g., patient records, informed consent forms, treatment documentation, information in patient diaries, MRI/CT images etc.

All source documents identifying patients shall remain at the investigational site. When these are transferred to the sponsor, the patient identifier will be removed and replaced with the patient ID.

Case report forms (CRF)

All patient data and examination results will be entered into the CRFs (case report forms) created specifically for this study.

The survey forms must only be completed with a pen or fineliner (black or blue). Corrections must be made in such a way that the old entry remains legible (the use of correctors is not permitted). Corrections must be signed and dated by the authorised person who makes them. Data that is not available or was not collected must be clearly recognisable as such (M or ND). Any reasons for this should be documented.

The study PI shall ensure that all data is entered into the CRFs immediately, legibly, completely, correctly and in accordance with the patient files. The original pages completed will be given to the person in charge of data input and evaluation.

10.5 Data Management

Data collection will be performed by the data management team using a systematic and structured procedure described in Chapters 4.6 and 10.1. The data management team will consist of the statistician and two members of the research team at the study site.

Handling of source data is described in Chapter10.4. The data will be reviewed and cleaned involving removal of duplicates and irrelevant entries, numerical and typographical entry errors (using the data quality checks described in Chapter 10.6.), structural errors and other identified inconsistencies in the data. Outliers will be identified using statistical methods and visual checks of histograms and boxplots and dealt with appropriately. The procedure of issuing and resolving data queries is described in Chapter 10.7.

Timely and reliable processes for recording data and rectifying errors and omissions will be performed to ensure an appropriate implementation of the planned statistical and qualitative data analyses and delivering high-quality data and achieve the a priori stated objectives of the clinical investigation. Procedures for maintaining and protecting patients' privacy are described in Chapter 12.5. In addition, procedures for the data storage including the data storage period are outlined in Chapter 10.3. Access to the data and documents will be limited to the PI and investigator, and members of the study team as authorised by the PI.

10.6 Data quality assurance measures and monitoring

Data input and export

For the statistical analysis, data from questionnaires and physical performance tests will be compiled and entered into the case report form (CRF) for each measurement point (screening, baseline examination, post-intervention examination) by an authorised and trained employee. The CRF only records data that is listed in the protocol and that is required for the interpretation of the study results. The data can then be fed from Excel into the statistics programme.

Quality assurance measures and monitoring

The following measures are implemented to ensure the plausibility and integrity of the data:

- Documentation of protocol deviations and the evaluation of whether they had an impact on data quality
- Data verification – For certain data fields, spot checks are carried out of value ranges and field types, and logical checks are also performed. When checking value ranges, the fields are checked for permitted values or the allowed number of possible responses (for multiple choice). When checking field types, the check concerns whether the values entered fit with the field definition (e.g., numerical fields). Logical checks check time spans (e.g., the order of visits/treatment units) or treatment and protocol compliance (i.e., by checking free-text fields).
- Duplicate data entry – the CRF data is entered into the database by two independent investigators. Data reconciliation is then carried out to find potential errors during manual input and detect missing data. Any discrepancies identified are rectified by a third, authorized person until no more differences exist.
- Data checks by the PI – before data analysis of the primary endpoints and secondary endpoints.
- Data quality will be promoted by duplicate (10MWT) or triple measurements (MFRT).

The check of data focuses on the data for key criteria, patient safety and protocol deviations.

10.7 Handling queries

The review of data will involve the aforementioned checks for completeness and medical plausibility. Any queries that arise will be recorded on specific questionnaires and forwarded to PI. The PI must use the questionnaires to check the discrepancies and respond to them. These forms are then forwarded to the data management team, where these discrepancies can be corrected in the database accordingly. The questionnaires will be stored along with the survey forms at the trial site, initially with the data management team and finally with the study PI.

At the end of the clinical investigation, the database will closed once all entries have been made and queries have been resolved. This process is documented.

11. REPORTS

11.1. Report of the study results

After the end of the clinical investigation and following the statistical analysis, drafting of a final report of the study results will be performed by the PI and study team for all endpoints and supported and evaluated by all responsible individuals. All information contained in the report of the initial study results is strictly confidential. The final study report will be submitted to the sponsor.

11.2. Publication

The outcomes of this study will be published. Data protection for all patient data will be guaranteed in all publications. The authors shall be the PI and study team at the study site substantially contributing to the study and manuscript drafting, and all authors must consent to the publication. The draft of the publication must be sent to the sponsor for evaluation before submission to a peer-reviewed journal.

12. ETHICS AND GOVERNANCE

12.1 Compliance with ethical and regulatory requirements

The study was planned in accordance with the requirements of the Tirol Health Insurance Act (Tir KAG), the OeAWI Guidelines for Good Scientific Practice and the regulations of the General Data Protection Regulation (GDPR) and the Austrian Data Protection Act (DSG).

The protocol development is further based on the Standard Protocol Items for Clinical Trials (SPIRIT; Statement 2013), the World Medical Association (WMA) Declaration of Helsinki - Ethical Principles for Medical Research involving human subjects (2013), the guidelines of the International Organization for Standardization (ISO), ICH E6 Guideline for good clinical practice (2016), the guideline for the clinical investigation of medical devices for human subjects - Good clinical practice (ISO/DIS 14155:2018) and the Template for Intervention Description and Replication (TIDieR) (2014) guidelines.

This study is an “Other clinical investigation” according to Art. 82 paragraph 1 of the MDR (EU) 2017/745. An “other clinical investigation” according to Annex XIV Part A Number 1 lit a EU-V. 2017/745 has been defined as a clinical investigation that

a) is **not part of** a systematic and planned process for **product development** or the manufacturer's product monitoring,

b) does not aim to prove conformity, and

c) serves to answer scientific or other questions and is **outside of the clinical development plan**.

The clinical investigation will be carried out according to the principle of equipoise, in the sense that all participants are treated equally (communication, appreciation, compliance with moral principles, the same number and duration of treatments). The four ethical principles of justice, autonomy, beneficience and non-maleficience, particularly with regard to the improvement of health and the protection of health, will be adhered to (Beauchamp & Childress, 2009).

12.2 Ethics approval

Before the start of the clinical investigation, approval for the clinical investigation plan, the informed consent form, amendments to these documents and other relevant clinical investigation documents will be sought and obtained from the ethics committee (EC) of the Medical University of Innsbruck. The EC must be informed of any subsequent amendments to the aforementioned documents.

12.3 Patient information and consent form

Before the clinical investigation begins, informed consent will be obtained from the patients by the investigator after they have been fully informed verbally and in writing in a way they understand about the nature, significance, and scope of the clinical investigation. The content of this information will be documented on the informed consent form. The patient will be informed if new information about the investigated intervention arises during the clinical investigation.

The patient's informed consent to participation in the clinical trial will be signed and dated by the patient and the doctor. The patient will receive a signed copy of the patient information/informed consent form. The doctor will file the second copy in the Investigator Site File. It is explicitly stated that no examinations or interventions in connection with the clinical investigation can be performed until a legally valid informed consent form has been obtained from the patient.

12.4 Patient insurance

Personal injury insurance will be taken out for all patients on behalf of the sponsor. This insurance will cover any potential damage that the patient may suffer directly or indirectly as a result of the clinical investigation interventions or procedures in connection with the clinical investigation.

In order not to jeopardise your insurance cover, the patients must follow the trial personnel's instructions exactly. In addition, patients must not undergo any other medical

treatments during the clinical investigation except those, which they receive during inpatient rehabilitation at the Reha Zentrum Münster without the investigator's permission (except for in an emergency). Patients must inform investigators of any emergency treatment immediately. Health damage that may occur as a consequence of the clinical investigation must be reported immediately to the investigator by the patient and the insurer by the PI. In addition, the insured party must take all appropriate measures to clarify the cause and extent of the damage occurred. The patient can ask the investigator if they wish to view the insurance conditions or receive a copy of them.

12.5 Data protection and confidentiality

Personal data of the patients will be collected, forwarded, stored and evaluated as part of this clinical investigation only in accordance with the legal provisions of the General Data Protection Regulation (EU) 2016/679 (GDPR) and the Austrian Data Protection Act (DSG). This requires the voluntary consent of the patient in the informed consent form before participation in the clinical investigation. Patients will be informed of the following in the informed consent for this clinical trial:

1. Data collected as part of this clinical trial will be recorded on paper CRFs or electronic data carriers, treated strictly confidentially and forwarded without your name (pseudonymised) to

   - The principle investigator of the clinical investigation for the evaluation of adverse events,

   - The sponsor of the clinical investigation in the case of serious adverse events,

   - The statistician of the clinical investigation for scientific analysis,

   - The relevant ethics committee of the Medical University of Innsbruck to review that the clinical investigation is being conducted correctly as well as to evaluate clinical investigation results and adverse events.

2. Documents that contain personal data (e.g., the signed patient consent form, patient identification list) shall remain at the relevant clinical investigation site and is subject to investigators' confidentiality.

3. Insofar as this is necessary for reviewing the clinical study, authorised representatives of the relevant supervisory authorities bound to secrecy may inspect the personal data held by the investigator. The investigator shall be released from their duty of medical confidentiality for this purpose.

4. The consent to the collection and processing of personal data as part of this clinical study cannot be revoked. Patients will be informed that they can stop participation in the study at any time, without having to give a reason or without any subsequent disadvantages. In the event of withdrawal of consent, the data not containing my name stored up until this point may continue to be used, where this is required in order to determine the effects of the clinical investigation intervention and make sure that the legitimate interests of the person concerned are not impacted.

12.6 Changes while the clinical investigation is being conducted

Protocol amendments

In the interest of flawless data evaluation, it is not intended for there to be changes from the clinical investigation conditions arranged and included in the protocol.

In exceptional cases, changes to clinical investigation conditions are possible, however. These will only take place following mutual agreement between the PI, investigator and the sponsor. Any change to the clinical investigation procedures set out in the protocol must be made in writing, specifying the reasons in question, and signed by all clinical investigation officers. The changes will then be part of the protocol. If required (e.g., in the case of significant changes that have a direct impact on the safety of clinical investigation participants), the consent of the responsible ethics committee and/or authorities and the patient must be obtained for protocol amendments.

The investigator has the option to stop the clinical investigation overall in emergency situations between the application for a protocol amendment and such amendments being rejected or coming into effect. This is mandatory if a protocol amendment or a change in the state of knowledge for clinical investigation-specific examinations occurs that leads to a potential reassessment of the risk/benefit assessment. In this case, the clinical investigation will only be continued once an updated opinion has been received from the ethics committee.

Deviations from the protocol

Deviations from the protocol should be avoided as much as possible. If deviations have to occur, they must be documented immediately and reported to the sponsor. The deviation must be evaluated by the sponsor in terms of its effects on patient safety and data quality and, if necessary, relevant action must be taken. A list of protocol deviations will be provided to the person who performs the data analysis along with the data export.

12.7 Informing clinical investigation participants about the study results

After the end of the clinical investigation, the final data analysis and once the final clinical investigation report has been written, the study participants will be informed of the results of the clinical investigation via mail. This information will exclusively include pseudonymised data.

12.8 Declaration of conflict of interest

The members of the clinical investigation team report having no conflicts of interest of a private, commercial, or financial nature. It is a prospective, prospective comparative multiple case study involving a physiotherapeutic RAGT intervention performed in clinical routine within inpatient rehabilitation and data collection primarily related to walking and balance performance tests, self-report questionnaires and non-invasive neurological examinations. None of the investigators will receive payment for their work.

12.9 Remuneration for clinical investigation participants

The clinical investigation participants will not receive any payment or compensation for their participation in the clinical investigation.

13. REFERENCES

A. T. S. Committee on Proficiency Standards for Clinical Pulmonary Function Laboratories. (2002). ATS statement: guidelines for the six-minute walk test. *American Journal of Respiratory and Critical Care Medicine*, *166*(1), 111-117. https://doi.org/10.1164/ajrccm.166.1.at1102

Ababacar, K., Diop, S., & Liu, E. (2020). Categorization of case in case study research method: new approach. *Knowledge and Performance Management*, *4*(1), 1-14. https://doi.org/10.21511/kpm.04(1).2020.01

Abdulla, S., Vielhaber, S., Körner, S., Machts, J., Heinze, H. J., Dengler, R., & Petri, S. (2013). Validation of the German version of the extended ALS functional rating scale as a patient-reported outcome measure. *J Neurol*, *260*(9), 2242-2255. https://doi.org/10.1007/s00415-013-6955-6

Anckar, C. (2008). On the Applicability of the Most Similar Systems Design and the Most Different Systems Design in Comparative Research. *International Journal of Social Research Methodology*, *11*(5), 389-401. https://doi.org/10.1080/13645570701401552

Asbury, A. K., & Cornblath, D. R. (1990). Assessment of current diagnostic criteria for Guillain-Barré syndrome. *Annals of neurology*, *27 Suppl*, S21-24. https://doi.org/10.1002/ana.410270707

Beauchamp, T. L., & Childress, J. F. (2009). *Principles of biomedical ethics* (6th ed ed.). Oxford University Press. https://doi.org/10.1007/s00481-010-0069-9

Berger, K., Weltermann, B., Kolominsky-Rabas, P., Meves, S., Heuschmann, P., Böhner, J., . . . Büttner, T. (1999). [The reliability of stroke scales. The german version of NIHSS, ESS

and Rankin scales]. *Fortschr Neurol Psychiatr*, *67*(2), 81-93. https://doi.org/10.1055/s-2007-993985 (Untersuchung zur Reliabilität von Schlanganfallskalen. Die deutschen Versionen von NIHSS, ESS und Rankin Scale.)

Bird, T. D. (1993). Hereditary Ataxia Overview. In M. P. Adam, H. H. Ardinger, R. A. Pagon, S. E. Wallace, L. J. H. Bean, G. Mirzaa, & A. Amemiya (Eds.), *GeneReviews(®)*. University of Washington, Seattle

Copyright © 1993-2021, University of Washington, Seattle. GeneReviews is a registered trademark of the University of Washington, Seattle. All rights reserved.

Bohannon, R. W. (1997). Comfortable and maximum walking speed of adults aged 20-79 years: reference values and determinants. *Age Ageing*, *26*(1), 15-19. http://www.ncbi.nlm.nih.gov/pubmed/9143432

Bohannon, R. W., & Smith, M. B. (1987). Interrater reliability of a modified Ashworth scale of muscle spasticity. *Physical therapy*, *67*(2), 206-207. https://doi.org/10.1093/ptj/67.2.206

Bouwens, S. F., van Heugten, C. M., & Verhey, F. R. (2009). The practical use of goal attainment scaling for people with acquired brain injury who receive cognitive rehabilitation. *Clin Rehabil*, *23*(4), 310-320. https://doi.org/10.1177/0269215508101744

Boyd, L. A., Vidoni, E. D., & Wessel, B. D. (2010). Motor learning after stroke: is skill acquisition a prerequisite for contralesional neuroplastic change? *Neuroscience letters*, *482*(1), 21-25. https://doi.org/10.1016/j.neulet.2010.06.082

Braun, V., & Clarke, V. (2006). Using thematic analysis in psychology. *Qualitative Research in Psychology*, *3*(2), 77-101. https://doi.org/10.1191/1478088706qp063oa

Braun, V., & Clarke, V. (2021). Conceptual and design thinking for thematic analysis. *Qualitative Psychology*, No Pagination Specified-No Pagination Specified. https://doi.org/10.1037/qup0000196

Brooks, B. R. (1994). El Escorial World Federation of Neurology criteria for the diagnosis of amyotrophic lateral sclerosis. Subcommittee on Motor Neuron Diseases/Amyotrophic Lateral Sclerosis of the World Federation of Neurology Research Group on Neuromuscular Diseases and the El Escorial "Clinical limits of amyotrophic lateral sclerosis" workshop contributors. *Journal of the neurological sciences*, *124 Suppl*, 96-107. https://doi.org/10.1016/0022-510x(94)90191-0

Brooks, B. R., Miller, R. G., Swash, M., & Munsat, T. L. (2000). El Escorial revisited: revised criteria for the diagnosis of amyotrophic lateral sclerosis. *Amyotroph Lateral Scler Other Motor Neuron Disord*, *1*(5), 293-299. https://doi.org/10.1080/146608200300079536

Brott, T., Adams, H. P., Jr., Olinger, C. P., Marler, J. R., Barsan, W. G., Biller, J., . . . et al. (1989). Measurements of acute cerebral infarction: a clinical examination scale. *Stroke*, *20*(7), 864-870. https://doi.org/10.1161/01.str.20.7.864

Brown, L., Cai, T., & DasGupta, A. (2001). Interval estimation for a binomial proportion. *Statistical Science*, *16*(2), 101-133.

Bruni, M. F., Melegari, C., De Cola, M. C., Bramanti, A., Bramanti, P., & Calabrò, R. S. (2018). What does best evidence tell us about robotic gait rehabilitation in stroke patients: A systematic review and meta-analysis. *J Clin Neurosci*, *48*, 11-17. https://doi.org/10.1016/j.jocn.2017.10.048

Calabrò, R. S., Cacciola, A., Bertè, F., Manuli, A., Leo, A., Bramanti, A., . . . Bramanti, P. (2016). Robotic gait rehabilitation and substitution devices in neurological disorders: where are we now? *Neurol Sci*, *37*(4), 503-514. https://doi.org/10.1007/s10072-016-2474-4

Calabrò, R. S., Naro, A., Russo, M., Bramanti, P., Carioti, L., Balletta, T., . . . Bramanti, A. (2018). Shaping neuroplasticity by using powered exoskeletons in patients with stroke: a randomized clinical trial. *J Neuroeng Rehabil*, *15*(1), 35. https://doi.org/10.1186/s12984-018-0377-8

Calabrò, R. S., Portaro, S., Manuli, A., Leo, A., Naro, A., & Bramanti, A. (2019). RETHINKING THE ROBOTIC REHABILITATION PATHWAY FOR PEOPLE WITH AMYOTROPHIC LATERAL SCLEROSIS: A NEED FOR CLINICAL TRIALS. *Innov Clin Neurosci*, *16*(1-2), 11-12.

Capecci, M., Pournajaf, S., Galafate, D., Sale, P., Le Pera, D., Goffredo, M., . . . Franceschini, M. (2019). Clinical effects of robot-assisted gait training and treadmill training for Parkinson's disease. A randomized controlled trial. *Annals of Physical and Rehabilitation Medicine*, *62*(5), 303-312. https://doi.org/https://doi.org/10.1016/j.rehab.2019.06.016

Cedarbaum, J. M., Stambler, N., Malta, E., Fuller, C., Hilt, D., Thurmond, B., & Nakanishi, A. (1999). The ALSFRS-R: a revised ALS functional rating scale that incorporates assessments of respiratory function. BDNF ALS Study Group (Phase III). *Journal of the neurological sciences*, *169*(1-2), 13-21. https://doi.org/10.1016/s0022-510x(99)00210-5

Chan, A. W., Tetzlaff, J. M., Altman, D. G., Laupacis, A., Gotzsche, P. C., Krleza-Jeric, K., . . . Moher, D. (2013). SPIRIT 2013 statement: defining standard protocol items for clinical trials. *Ann Intern Med*, *158*(3), 200-207. https://doi.org/10.7326/0003-4819-158-3-201302050-00583

Cheng, Y.-Y., Hsieh, W.-L., Kao, C.-L., & Chan, R.-C. (2012). Principles of rehabilitation for common chronic neurologic diseases in the elderly. *Journal of Clinical Gerontology and Geriatrics*, *3*(1), 5-13. https://doi.org/https://doi.org/10.1016/j.jcgg.2011.11.003

Criteria for diagnosis of Guillain-Barré syndrome. (1978). *Annals of neurology*, *3*(6), 565-566. https://doi.org/10.1002/ana.410030628

de Carvalho, M., Dengler, R., Eisen, A., England, J. D., Kaji, R., Kimura, J., . . . Swash, M. (2008). Electrodiagnostic criteria for diagnosis of ALS. *Clin Neurophysiol*, *119*(3), 497-503. https://doi.org/10.1016/j.clinph.2007.09.143

de Silva, R., Greenfield, J., Cook, A., Bonney, H., Vallortigara, J., Hunt, B., & Giunti, P. (2019). Guidelines on the diagnosis and management of the progressive ataxias. *Orphanet journal of rare diseases*, *14*(1), 51-51. https://doi.org/10.1186/s13023-019-1013-9

Diagnose und Therapie des Guillain-Barré Syndroms im Kindes- und Jugendalter, 4. Auflage, Version 1.0. Gesellschaft für Neuropädiatrie. Retrieved 06.08. from https://www.awmf.org/leitlinien/detail/ll/022-008.html

Dobkin, B. H. (2005). Rehabilitation and functional neuroimaging dose-response trajectories for clinical trials. *Neurorehabilitation and neural repair*, *19*(4), 276-282. https://doi.org/10.1177/1545968305281892

Duncan, P. W., Weiner, D. K., Chandler, J., & Studenski, S. (1990). Functional reach: a new clinical measure of balance. *J Gerontol*, *45*(6), M192-197. https://doi.org/10.1093/geronj/45.6.m192

European Federation of Neurological Societies/Peripheral Nerve Society Guideline on management of chronic inflammatory demyelinating polyradiculoneuropathy: report of a joint task force of the European Federation of Neurological Societies and the

Peripheral Nerve Society--First Revision. (2010). *J Peripher Nerv Syst*, *15*(1), 1-9. https://doi.org/10.1111/j.1529-8027.2010.00245.x

Fahn, S., & Elton, R. L. (1987). Unified Parkinson's disease rating scale. In S. Fahn, G. M., M. D., & C. D.B. (Eds.), *Recent de-velopments in Parkinson's disease* (Vol. II, pp. 153–163). Mac-Millan.

Fischer, N. (2014). Trunk Control Test – Rumpfkontrolle erfassen. *ergopraxis*, *7*(06), 32-33.

Franchignoni, F. P., Tesio, L., Ricupero, C., & Martino, M. T. (1997). Trunk control test as an early predictor of stroke rehabilitation outcome. *Stroke*, *28*(7), 1382-1385. https://doi.org/10.1161/01.str.28.7.1382

Freivogel, S., Mehrholz, J., Husak-Sotomayor, T., & Schmalohr, D. (2008). Gait training with the newly developed 'LokoHelp'-system is feasible for non-ambulatory patients after stroke, spinal cord and brain injury. A feasibility study. *Brain Injury*, *22*(7-8), 625-632. https://doi.org/10.1080/02699050801941771

Gentile, A. M. (1972). A Working Model of Skill Acquisition with Application to Teaching. *Quest*, *17*(1), 3-23. https://doi.org/10.1080/00336297.1972.10519717

Goodrick, D. (2014). *Comparative Case Studies, Methodological Briefs: Impact Evaluation 9*. UNICEF Office of Research.

Graham, R. C., & Hughes, R. A. (2006). A modified peripheral neuropathy scale: the Overall Neuropathy Limitations Scale. *J Neurol Neurosurg Psychiatry*, *77*(8), 973-976. https://doi.org/10.1136/jnnp.2005.081547

Hadden, R. D., Cornblath, D. R., Hughes, R. A., Zielasek, J., Hartung, H. P., Toyka, K. V., & Swan, A. V. (1998). Electrophysiological classification of Guillain-Barré syndrome: clinical associations and outcome. Plasma Exchange/Sandoglobulin Guillain-Barré Syndrome Trial Group. *Annals of neurology*, *44*(5), 780-788. https://doi.org/10.1002/ana.410440512

Harb, A., & Kishner, S. (2021). Modified Ashworth Scale. In *StatPearls*. StatPearls Publishing Copyright © 2021, StatPearls Publishing LLC.

Heale, R., & Forbes, D. (2013). Understanding triangulation in research. *Evidence Based Nursing*, *16*(4), 98. https://doi.org/10.1136/eb-2013-101494

Hennerici, M. G., & Kern, R. e. a. (2017). *S1-Leitlinie Diagnostik akuter zerebrovaskulärer Erkrankungen*. Deutsche Gesellschaft für Neurologie Retrieved 13.02. from www.dgn.org/leitlinien

Herdman, M., Gudex, C., Lloyd, A., Janssen, M., Kind, P., Parkin, D., . . . Badia, X. (2011). Development and preliminary testing of the new five-level version of EQ-5D (EQ-5D-5L). *Qual Life Res*, *20*(10), 1727-1736. https://doi.org/10.1007/s11136-011-9903-x

Hoehn, M. M., & Yahr, M. D. (1967). Parkinsonism: onset, progression and mortality. *Neurology*, *17*(5), 427-442. https://doi.org/10.1212/wnl.17.5.427

Hsieh, C. L., Sheu, C. F., Hsueh, I. P., & Wang, C. H. (2002). Trunk control as an early predictor of comprehensive activities of daily living function in stroke patients. *Stroke*, *33*(11), 2626-2630. https://doi.org/10.1161/01.str.0000033930.05931.93

Hurn, J., Kneebone, I., & Cropley, M. (2006). Goal setting as an outcome measure: A systematic review. *Clin Rehabil*, *20*(9), 756-772. https://doi.org/10.1177/0269215506070793

Jarvis, H. L., Brown, S. J., Price, M., Butterworth, C., Groenevelt, R., Jackson, K., . . . Reeves, N. D. (2019). Return to Employment After Stroke in Young Adults. *Stroke*, *50*(11), 3198-3204. https://doi.org/doi:10.1161/STROKEAHA.119.025614

Johnsen, B. (2020). Diagnostic criteria for amyotrophic lateral sclerosis from El Escorial to Gold Coast. *Clin Neurophysiol*, *131*(8), 1962-1963. https://doi.org/10.1016/j.clinph.2020.04.012

Jørgensen, H. S., Nakayama, H., Raaschou, H. O., & Olsen, T. S. (1995). Recovery of walking function in stroke patients: the Copenhagen Stroke Study. *Archives of physical medicine and rehabilitation*, *76*(1), 27-32. https://doi.org/10.1016/s0003-9993(95)80038-7

Katz-Leurer, M., Fisher, I., Neeb, M., Schwartz, I., & Carmeli, E. (2009). Reliability and validity of the modified functional reach test at the sub-acute stage post-stroke. *Disability and rehabilitation*, *31*(3), 243-248. https://doi.org/10.1080/09638280801927830

Kiresuk, T. J., & Sherman, R. E. (1968). Goal attainment scaling: A general method for evaluating comprehensive community mental health programs. *Community Ment Health J*, *4*(6), 443-453. https://doi.org/10.1007/bf01530764

Kirshblum, S. C., Burns, S. P., Biering-Sorensen, F., Donovan, W., Graves, D. E., Jha, A., . . . Waring, W. (2011). International standards for neurological classification of spinal cord injury (revised 2011). *J Spinal Cord Med*, *34*(6), 535-546. https://doi.org/10.1179/204577211x13207446293695

Klotz, S. G. R., Petersen-Ewert, C., Ketels, G., Scherer, M., & Barzel, A. (2019). The German version of the Functional Walking Categories (FWC): translation and initial validation. *Top Stroke Rehabil*, *26*(1), 49-57. https://doi.org/10.1080/10749357.2018.1536022

Krupp, L. B., LaRocca, N. G., Muir-Nash, J., & Steinberg, A. D. (1989). The Fatigue Severity Scale: Application to Patients With Multiple Sclerosis and Systemic Lupus Erythematosus. *Arch Neurol*, *46*(10), 1121-1123. https://doi.org/10.1001/archneur.1989.00520460115022

Kurtzke, J. F. (1983). Rating neurologic impairment in multiple sclerosis: an Expanded Disability Status Scale (EDSS). *Neurology*, *33*(11), 1444-1452. http://www.ncbi.nlm.nih.gov/pubmed/6685237

Kwakkel, G. (2006). Impact of intensity of practice after stroke: issues for consideration. *Disability and rehabilitation*, *28*(13-14), 823-830. https://doi.org/10.1080/09638280500534861

Little, R. J. A. (1988). A Test of Missing Completely at Random for Multivariate Data with Missing Values. *Journal of the American Statistical Association*, *83*(404), 1198-1202. https://doi.org/10.1080/01621459.1988.10478722

Luca, A. D., Lentino, C., Vernetti, H., Checchia, G. A., Giannoni, P., Morasso, P., & Casadio, M. (2013, 24-26 June 2013). Functional evaluation of robot end-point assisted gait re-education in chronic stroke survivors. 2013 IEEE 13th International Conference on Rehabilitation Robotics (ICORR),

Ludwig, K., Graf von der Schulenburg, J. M., & Greiner, W. (2018). German Value Set for the EQ-5D-5L. *Pharmacoeconomics*, *36*(6), 663-674. https://doi.org/10.1007/s40273-018-0615-8

Ludwig, K., von der Schulenburg, J. G., & Greiner, W. (2017). Valuation of the EQ-5D-5L with composite time trade-off for the German population - an exploratory study. *Health Qual Life Outcomes*, *15*(1), 39. https://doi.org/10.1186/s12955-017-0617-9

Lyden, P., Lu, M., Jackson, C., Marler, J., Kothari, R., Brott, T., & Zivin, J. (1999). Underlying structure of the National Institutes of Health Stroke Scale: results of a factor analysis. NINDS tPA Stroke Trial Investigators. *Stroke*, *30*(11), 2347-2354. https://doi.org/10.1161/01.str.30.11.2347

Maier, M., Ballester, B. R., & Verschure, P. (2019). Principles of Neurorehabilitation After Stroke Based on Motor Learning and Brain Plasticity Mechanisms. *Front Syst Neurosci*, *13*, 74. https://doi.org/10.3389/fnsys.2019.00074

Mazzoni, P., & Krakauer, J. W. (2006). An implicit plan overrides an explicit strategy during visuomotor adaptation. *J Neurosci*, *26*(14), 3642-3645. https://doi.org/10.1523/jneurosci.5317-05.2006

Mehrholz, J., Pohl, M., Kugler, J., & Elsner, B. (2018). The Improvement of Walking Ability Following Stroke. *Dtsch Arztebl Int*, *115*(39), 639-645. https://doi.org/10.3238/arztebl.2018.0639

Mehrholz, J., Thomas, S., Werner, C., Kugler, J., Pohl, M., & Elsner, B. (2017). Electromechanical-assisted training for walking after stroke. *Cochrane Database Syst Rev*, *5*(5), Cd006185. https://doi.org/10.1002/14651858.CD006185.pub4

Mehrholz, J., Wagner, K., Rutte, K., Meissner, D., & Pohl, M. (2007). Predictive validity and responsiveness of the functional ambulation category in hemiparetic patients after stroke. *Archives of physical medicine and rehabilitation*, *88*(10), 1314-1319. https://doi.org/10.1016/j.apmr.2007.06.764

Mulligan, M., Guess, D., Holvoet, J., & Brown, F. (1980). The Individualized Curriculum Sequencing Model (I): Implications from Research on Massed, Distributed, or Spaced Trial Training. *Journal of the Association for the Severely Handicapped*, *5*(4), 325-336. https://doi.org/10.1177/154079698000500403

Newcombe, R. G. (1998). Two-sided confidence intervals for the single proportion: comparison of seven methods. *Stat Med*, *17*(8), 857-872. https://doi.org/10.1002/sim.2164

Nilsson, L., Carlsson, J., Danielsson, A., Fugl-Meyer, A., Hellström, K., Kristensen, L., . . . Grimby, G. (2001). Walking training of patients with hemiparesis at an early stage after stroke: a comparison of walking training on a treadmill with body weight support and walking training on the ground. *Clin Rehabil*, *15*(5), 515-527. https://doi.org/10.1191/026921501680425234

Noble, H., & Heale, R. (2019). Triangulation in research, with examples. *Evidence Based Nursing*, *22*(3), 67. https://doi.org/10.1136/ebnurs-2019-103145

Nordbeck, R. (2013). Die vergleichende Methode als Forschungsansatz. In *Internationaler Politiktransfer und nationaler Politikwandel: Ausbreitung und Effektivität des Umweltaktionsprogramms in Mittel- und Osteuropa* (pp. 109-144). Springer Fachmedien Wiesbaden. https://doi.org/10.1007/978-3-658-00384-5_5

Ochoa-Morales, A., Hernández-Mojica, T., Paz-Rodríguez, F., Jara-Prado, A., Trujillo-De Los Santos, Z., Sánchez-Guzmán, M. A., . . . Dávila-Ortiz de Montellano, D. J. (2019). Quality of life in patients with multiple sclerosis and its association with depressive symptoms and physical disability. *Mult Scler Relat Disord*, *36*, 101386. https://doi.org/10.1016/j.msard.2019.101386

Osterberg, L., & Blaschke, T. (2005). Adherence to medication. *N Engl J Med*, *353*(5), 487-497. https://doi.org/10.1056/NEJMra050100

Pearson, O. R., Busse, M. E., van Deursen, R. W. M., & Wiles, C. M. (2004). Quantification of walking mobility in neurological disorders. *QJM: An International Journal of Medicine*, *97*(8), 463-475. https://doi.org/10.1093/qjmed/hch084

Persson, C. U., Sunnerhagen, K. S., & Lundgren-Nilsson, A. (2014). Rasch analysis of the modified version of the postural assessment scale for stroke patients: postural stroke

study in Gothenburg (POSTGOT). *BMC neurology*, *14*, 134. https://doi.org/10.1186/1471-2377-14-134

Pfeffer, A. (2008). Einsatz bei Erschöpfung. *Physiopraxis*, *6*(10), 42-43.

Pohl, M., Werner, C., Holzgraefe, M., Kroczek, G., Wingendorf, I., Hoölig, G., . . . Hesse, S. (2007). Repetitive locomotor training and physiotherapy improve walking and basic activities of daily living after stroke: a single-blind, randomized multicentre trial (DEutsche GAngtrainerStudie, DEGAS). *Clin Rehabil*, *21*(1), 17-27. https://doi.org/10.1177/0269215506071281

Polman, C. H., Reingold, S. C., Banwell, B., Clanet, M., Cohen, J. A., Filippi, M., . . . Wolinsky, J. S. (2011). Diagnostic criteria for multiple sclerosis: 2010 revisions to the McDonald criteria [Research Support, Non-U.S. Gov't]. *Annals of neurology*, *69*(2), 292-302. https://doi.org/10.1002/ana.22366

Portaro, S., Naro, A., Chillura, A., Billeri, L., Bramanti, A., Bramanti, P., . . . Calabrò, R. S. (2017). Toward a more personalized motor function rehabilitation in Myotonic dystrophy type 1: The role of neuroplasticity. *PLoS One*, *12*(5), e0178470. https://doi.org/10.1371/journal.pone.0178470

Potter, K., Cohen, E. T., Allen, D. D., Bennett, S. E., Brandfass, K. G., Widener, G. L., & Yorke, A. M. (2014). Outcome measures for individuals with multiple sclerosis: recommendations from the American Physical Therapy Association neurology section task force. *Physical therapy*, *94*(5), 593-608. https://doi.org/10.2522/ptj.20130149

Przeworski, A., & Teune, H. (1970). *The logic of comparative social inquiry*. John Wiley.

Raggi, A., Covelli, V., Schiavolin, S., Scaratti, C., Leonardi, M., & Willems, M. (2016). Work-related problems in multiple sclerosis: a literature review on its associates and determinants. *Disability and rehabilitation*, *38*(10), 936-944. https://doi.org/10.3109/09638288.2015.1070295

Roberts, T. T., Leonard, G. R., & Cepela, D. J. (2017). Classifications In Brief: American Spinal Injury Association (ASIA) Impairment Scale. *Clinical orthopaedics and related research*, *475*(5), 1499-1504. https://doi.org/10.1007/s11999-016-5133-4

Rose, D. K., Nadeau, S. E., Wu, S. S., Tilson, J. K., Dobkin, B. H., Pei, Q., & Duncan, P. W. (2017). Locomotor Training and Strength and Balance Exercises for Walking Recovery After Stroke: Response to Number of Training Sessions. *Physical therapy*, *97*(11), 1066-1074. https://doi.org/10.1093/ptj/pzx079

Rossier, P., & Wade, D. T. (2001). Validity and reliability comparison of 4 mobility measures in patients presenting with neurologic impairment. *Archives of physical medicine and rehabilitation*, *82*(1), 9-13. https://doi.org/10.1053/apmr.2001.9396

Salbach, N. M., Mayo, N. E., Higgins, J., Ahmed, S., Finch, L. E., & Richards, C. L. (2001). Responsiveness and predictability of gait speed and other disability measures in acute stroke. *Archives of physical medicine and rehabilitation*, *82*(9), 1204-1212. https://doi.org/10.1053/apmr.2001.24907

Salmoni, A. W., Schmidt, R. A., & Walter, C. B. (1984). Knowledge of results and motor learning: a review and critical reappraisal. *Psychol Bull*, *95*(3), 355-386.

Schmidt, R. A., & Lee, T. D. (2011). *Motor control and learning: A Behavioral Emphasis* (5th ed ed.). Human Kinetics. www.HumanKinetics.com

Schmitz-Hübsch, T., du Montcel, S. T., Baliko, L., Berciano, J., Boesch, S., Depondt, C., . . . Fancellu, R. (2006). Scale for the assessment and rating of ataxia: development of a new clinical scale. *Neurology*, *66*(11), 1717-1720. https://doi.org/10.1212/01.wnl.0000219042.60538.92

Shors, T. J., Anderson, M. L., Curlik, D. M., 2nd, & Nokia, M. S. (2012). Use it or lose it: how neurogenesis keeps the brain fit for learning. *Behav Brain Res*, *227*(2), 450-458. https://doi.org/10.1016/j.bbr.2011.04.023

Spinal injury: assessment and initial management. Spinal injury assessment: assessment and imaging for spinal injury. NICE Guideline NG41. (2016). NICE, National Institute for Health and Care Excellence. Retrieved 21.12. from https://www.nice.org.uk/guidance/ng41/evidence/full-guideline-2358425776

Subramanian, S. K., Massie, C. L., Malcolm, M. P., & Levin, M. F. (2010). Does provision of extrinsic feedback result in improved motor learning in the upper limb poststroke? A systematic review of the evidence. *Neurorehabilitation and neural repair*, *24*(2), 113-124. https://doi.org/10.1177/1545968309349941

Sullivan, K. J., Mulroy, S., & Kautz, S. A. (2009). Walking recovery and rehabilitation after stroke. In J. Stein, R. L. Harvey, R. F. Macko, C. J. Winstein, & R. D. Zorowitz (Eds.), *Stroke recovery and rehabilitation*. Demos Medical Publishing.

Taub, E., Uswatte, G., & Pidikiti, R. (1999). Constraint-Induced Movement Therapy: a new family of techniques with broad application to physical rehabilitation--a clinical review. *J Rehabil Res Dev*, *36*(3), 237-251.

Thompson, A. J., Banwell, B. L., Barkhof, F., Carroll, W. M., Coetzee, T., Comi, G., . . . Cohen, J. A. (2018). Diagnosis of multiple sclerosis: 2017 revisions of the McDonald criteria. *The Lancet Neurology*, *17*(2), 162-173. https://doi.org/10.1016/s1474-4422(17)30470-2

Traumatische Querschnittlähmung. (2019). In *Referenz Neurologie*. Georg Thieme Verlag. https://doi.org/10.1055/b-0039-170638

Uncini, A., Manzoli, C., Notturno, F., & Capasso, M. (2010). Pitfalls in electrodiagnosis of Guillain-Barré syndrome subtypes. *J Neurol Neurosurg Psychiatry*, *81*(10), 1157-1163. https://doi.org/10.1136/jnnp.2010.208538

Valko, P. O., Bassetti, C. L., Bloch, K. E., Held, U., & Baumann, C. R. (2008). Validation of the fatigue severity scale in a Swiss cohort. *Sleep*, *31*(11), 1601-1607. https://doi.org/10.1093/sleep/31.11.1601

van Hedel, H. J. (2009). Gait speed in relation to categories of functional ambulation after spinal cord injury. *Neurorehabilitation and neural repair*, *23*(4), 343-350. https://doi.org/10.1177/1545968308324224

van Hedel, H. J., Wirz, M., & Curt, A. (2006). Improving walking assessment in subjects with an incomplete spinal cord injury: responsiveness. *Spinal Cord*, *44*(6), 352-356. https://doi.org/10.1038/sj.sc.3101853

van Hedel, H. J., Wirz, M., & Dietz, V. (2005). Assessing walking ability in subjects with spinal cord injury: validity and reliability of 3 walking tests. *Archives of physical medicine and rehabilitation*, *86*(2), 190-196. https://doi.org/10.1016/j.apmr.2004.02.010

van Koningsveld, R., Steyerberg, E. W., Hughes, R. A. C., Swan, A. V., van Doorn, P. A., & Jacobs, B. C. (2007). A clinical prognostic scoring system for Guillain-Barré syndrome. *The Lancet Neurology*, *6*(7), 589-594. https://doi.org/10.1016/S1474-4422(07)70130-8

Van Peppen, R. P., Kwakkel, G., Wood-Dauphinee, S., Hendriks, H. J., Van der Wees, P. J., & Dekker, J. (2004). The impact of physical therapy on functional outcomes after stroke: what's the evidence? *Clin Rehabil*, *18*(8), 833-862. https://doi.org/10.1191/0269215504cr843oa

Veerbeek, J. M., van Wegen, E., van Peppen, R., van der Wees, P. J., Hendriks, E., Rietberg, M., & Kwakkel, G. (2014). What is the evidence for physical therapy poststroke? A

systematic review and meta-analysis. *PLoS One*, *9*(2), e87987. https://doi.org/10.1371/journal.pone.0087987

Wadden, K. P., Asis, K., Mang, C. S., Neva, J. L., Peters, S., Lakhani, B., & Boyd, L. A. (2017). Predicting Motor Sequence Learning in Individuals With Chronic Stroke. *Neurorehabilitation and neural repair*, *31*(1), 95-104. https://doi.org/10.1177/1545968316662526

Wade, D. T. (1992). *Measurement in neurological rehabilitation*. Oxford Medical Publications.

Wade, D. T., Wood, V. A., Heller, A., Maggs, J., & Langton Hewer, R. (1987). Walking after stroke. Measurement and recovery over the first 3 months. *Scand J Rehabil Med*, *19*(1), 25-30.

Watson, M. J. (2002). Refining the ten-metre walking test for use with neurologically impaired people. *Physiotherapy*, *88*(7), 386-397.

Weyer, A., Abele, M., Schmitz-Hübsch, T., Schoch, B., Frings, M., Timmann, D., & Klockgether, T. (2007). Reliability and validity of the scale for the assessment and rating of ataxia: a study in 64 ataxia patients. *Mov Disord*, *22*(11), 1633-1637. https://doi.org/10.1002/mds.21544

Wickens, C. D., Hutchins, S., Carolan, T., & Cumming, J. (2013). Effectiveness of part-task training and increasing-difficulty training strategies: a meta-analysis approach. *Hum Factors*, *55*(2), 461-470. https://doi.org/10.1177/0018720812451994

Willison, H. J., Jacobs, B. C., & van Doorn, P. A. (2016). Guillain-Barré syndrome. *The Lancet*, *388*(10045), 717-727. https://doi.org/10.1016/S0140-6736(16)00339-1

Wirz, M., Colombo, G., & Dietz, V. (2001). Long term effects of locomotor training in spinal humans. *J Neurol Neurosurg Psychiatry*, *71*(1), 93-96. https://doi.org/10.1136/jnnp.71.1.93

WMA. (2013). *Declaration of Helsinki - Ethical Principles for Medical Research involving human subjects*. World Medical Association. https://www.wma.net/policies-post/wma-declaration-of-helsinki-ethical-principles-for-medical-research-involving-human-subjects/

Wu, M., Landry, J. M., Kim, J., Schmit, B. D., Yen, S.-C., & MacDonald, J. (2014). Robotic Resistance/Assistance Training Improves Locomotor Function in Individuals Poststroke: A Randomized Controlled Study. *Archives of physical medicine and rehabilitation*, *95*(5), 799-806. https://doi.org/10.1016/j.apmr.2013.12.021

Wulf, G., & Prinz, W. (2001). Directing attention to movement effects enhances learning: a review. *Psychon Bull Rev*, *8*(4), 648-660. https://doi.org/10.3758/bf03196201

Yin, R. K. (2014). *Case Study Research: Design and Methods*
by (5th ed., Vol. 5). SAGE Publications Inc.
