# Supplementary material for: Robot-assisted gait training in patients with various neurological diseases: A mixed methods feasibility study
Source: PLoS One. 2024 Aug 27;19(8):e0307434. doi: 10.1371/journal.pone.0307434 (PMC11349200; doi:10.1371/journal.pone.0307434)
Supplement: S2 File — (DOCX) [file pone.0307434.s005.docx]

**S2 File. LEXO training protocol.**

**LEXO® training protocol**

**Surname, first name**: ………………………………………………………………………………… **Date of birth**: ……………………………………………

**Diagnose**: ……………………………………………………………………………………………… wheelchair ⃝ ambulatory ⃝

**Rehab goal**: …………………………………………………………………………………………… **Departure date**: ……………………………………….

**Important information**: ……………………………………………………………………………… **Harness:** ……………… **Leg straps:** ………………

**Harness positioning**: standing ⃝ seated ⃝ **Transfer**: lifting system ⃝ slide board ⃝ saddle ⃝ **Bar height**: ……….. (holes visible from above)

| **No.** | **Date, therapist** | **Distance (m)** | **Duration (min)** | **Number of steps** | **Stance phase % ri/le** | | | **Swing phase % ri/le** | | **Ø body weight support %** | | **Ø step length m** | | **Cadence (from/to)** | | **Max. speed. km/h (from/to)** | | **Pelvis support** | | **Chest support** | | **Comments**  **(breaks, reasons for termination, delayed start, anti-slip mat, general notes, …)** | |  |
| --- | --- | --- | --- | --- | --- | --- | --- | --- | --- | --- | --- | --- | --- | --- | --- | --- | --- | --- | --- | --- | --- | --- | --- | --- |
|  |  |  |  |  |  | |  |  |  |  |  |  |  |  |  |  |  |  |  |  |  |  |  |  |
| **1** |  |  |  |  |  | |  |  |  |  | |  | |  | |  | |  | |  | |  | |  |
| **2** |  |  |  |  |  | |  |  |  |  | |  | |  | |  | |  | |  | |  | |  |
| **3** |  |  |  |  |  |  | |  |  | |  | |  | |  | |  | |  | |  | |  | |
| **4** |  |  |  |  |  |  | |  |  | |  | |  | |  | |  | |  | |  | |  | |
| **5** |  |  |  |  |  |  | |  |  | |  | |  | |  | |  | |  | |  | |  | |
| **6** |  |  |  |  |  |  | |  |  | |  | |  | |  | |  | |  | |  | |  | |
| **7** |  |  |  |  |  |  | |  |  | |  | |  | |  | |  | |  | |  | |  | |
| **8** |  |  |  |  |  |  | |  |  | |  | |  | |  | |  | |  | |  | |  | |
| **9** |  |  |  |  |  |  | |  |  | |  | |  | |  | |  | |  | |  | |  | |
| **10** |  |  |  |  |  |  | |  |  | |  | |  | |  | |  | |  | |  | |  | |
| **11** |  |  |  |  |  |  | |  |  | |  | |  | |  | |  | |  | |  | |  | |
| **12** |  |  |  |  |  |  | |  |  | |  | |  | |  | |  | |  | |  | |  | |
| **13** |  |  |  |  |  | |  |  |  |  | |  | |  | |  | |  | |  | |  | |  |
| **14** |  |  |  |  |  | |  |  |  |  | |  | |  | |  | |  | |  | |  | |  |
| **15** |  |  |  |  |  | |  |  |  |  | |  | |  | |  | |  | |  | |  | |  |
| **16** |  |  |  |  |  | |  |  |  |  | |  | |  | |  | |  | |  | |  | |  |
