# Supplementary material for: Robot-assisted gait training in patients with various neurological diseases: A mixed methods feasibility study
Source: PLoS One. 2024 Aug 27;19(8):e0307434. doi: 10.1371/journal.pone.0307434 (PMC11349200; doi:10.1371/journal.pone.0307434)
Supplement: S3 File — (DOCX) [file pone.0307434.s006.docx]

**S3 File. Description and psychometric properties of assessments.**

The Functional Ambulation Categories (FAC) [1, 2] was used in its validated German version [3] to screen patients with respect to their walking ability and to assess walking ability at post-intervention. The FAC is a 6-point functional walking test that evaluates ambulation ability, determining how much human support the patient requires when walking, regardless of whether they use a personal assistive device. The FAC is a rapid, straightforward, and cost-effective visual measurement of walking, requiring only a set of stairs and a 15-meter indoor pathway for administration.

The 10**-**Metre Walk Test (10MWT) [4] is a performance measure used to assess walking speed in metres per second over a short distance. It can be employed to determine functional mobility in various neurological patient populations. Originally designed for stroke patients [5], it has been shown to be valid and reliable in a mixed group of patients with a neurological disorder [6]. Its psychometric properties have been well investigated in adult patients with incomplete spinal cord injuries, demonstrating good reliability and excellent responsiveness [7, 8]. In these patients, walking speed is also a good predictor of functional walking performance [9]. For adult stroke patients, the 10MWT has proven to be sensitive to change [10]. The test requires only a stopwatch and a 10-meter pathway with markings at 2 and 8 metres. Participants are timed from the moment their toes cross the 2-meter mark to when they cross the 8-meter mark. It can be conducted at preferred or maximum walking speed; in this study, it was performed at maximum speed, with two trials and an average calculated [11].

The Functional Gait Assessment (FGA) [12] was to assess dynamic balance during walking and a patient's ability to perform multiple motor tasks while walking. consists of 10 items assessing various walking conditions, including walking at different speeds, with head turns, quick turns, stops, climbing stairs, and negotiating obstacles. Each item is scored on a scale of 0-3 points, with 3 indicating optimal performance. The total score ranges from 0 to 30, with higher scores indicating better dynamic balance. The assessment typically takes 5-20 minutes (Thieme et al., 2009).

The 6-Minute Walk Test (6MWT) involves participants covering as much ground as possible in 6 minutes along a 30-meter hallway, with markers at each end for turning points. Warm-up is not permitted before the test, as per American Thoracic Society (A.T.S.) guidelines [13]. Participants rest for at least 10 minutes near the starting line before the test, and a second chair for rest is positioned near the end of the hallway but out of the walking path. Assistive devices may be used but must be consistent and documented between tests. Rests are allowed at any time during the 6 minutes, and the number and duration of rests are recorded [14]. Encouragement is provided every minute with a standardised voice, but no additional verbal or non-verbal cues are given [13]. The number of laps from the starting point is counted and displayed to the participant, and the distance in meters for the final partial lap is measured with a calibrated wheel.

The Timed-Up and Go (TUG) was used to measure functional mobility [15]. and assess the risk of falling during everyday movements. It includes transferring from sitting to standing, walking, turning, returning to the chair, and sitting down. The test begins with the patient seated on a chair of approximately 46 cm height. The time it takes for the patient to stand, walk three meters, turn around, return to the chair, and sit down is recorded in seconds. Walking aids are allowed and documented. The TUG typically takes less than 5 minutes to complete.

The Four Step Square Test (FSST) [16] evaluates balance and the ability to step over obstacles in forward, sideways, and backward directions. Patients stand within a marked square on the floor and step in clockwise and counterclockwise directions. Two runs are performed: one for practice and one timed. The test takes less than 5 minutes to complete.

The Trunk Control Test (TCT) [17] which was used in its German version [18] assesses trunk mobility in non-ambulatory patients after stroke in the subacute stage. It evaluates four movements: rolling from supine to weak and strong sides, sitting up from lying down, and sitting balance. Scores range from 0 to 100 based on the patient's ability to perform these movements. Higher scores indicate better trunk mobility [17, 19].

The Modified Functional Reach Test (MFRT) measures trunk movement limits in non-ambulatory patients after stroke. It is based on the Functional Reach Test [20] and assesses how far a patient can reach forward while sitting [21, 22]. The MFRT involves three conditions, each with three trials, measuring reach in various directions without rotating or touching the wall. Results are recorded in centimetres. A 15-second rest is provided between trials [22].

Many individuals with neurological disabilities and older adults experience psychological issues related to falls, whether they have fallen before or not. To address these concerns, assessing fear of falling and self-efficacy related to falling is necessary. The Falls Efficacy Scale (FES) [23] was expanded in 2005 by the Prevention of Falls Network Europe (ProFaNE) to include more complex functional activities and social aspects of self-efficacy [24]. It consists of 16 items rated on a 4-point Likert scale ranging from 1 = “no concerns” to 4 = “very serious concerns” and takes 5-10 minutes to complete. The German FES-I version was used in this study [25].

The 5-level EQ-5D version (EQ-5D-5L) [26] was used in its validated German version [27, 28] to assess health-related quality of life (HRQoL). The EQ-5D-5L consists of two parts: the descriptive system and the EQ Visual Analogue Scale (EQ VAS). The descriptive system evaluates five dimensions of health - mobility, self-care, usual activities, pain/discomfort and anxiety/depression, each with five levels of severity. The EQ VAS measures self-rated health on a 0-100 scale, where the endpoints are labelled ‘The best health you can imagine’ and ‘The worst health you can imagine’. It provides a quantitative measure of health outcome based on the patient's judgment.

The Beck Depression Inventory revised (BDI-II) (Beck et al., 1988) was used to measure the severity of depression. It consists of 21 items rated on a 4-point Likert scale, with scores ranging from 0 to 63. It is validated for individuals aged 13 and older and takes 5-10 minutes to complete.

The Fatigue Severity Scale (FSS) [29] was utilised in its validated German version [30] to assess fatigue in this study. The FSS is one of the most frequently used inventories for measuring fatigue in people with chronic illnesses including those with neurological diseases. It consists of nine items rated on a 7-point Likert scale ranging from 1 (‘strongly disagree’) to 7 (‘strongly agree’), with a mean score used to indicate fatigue severity. A cutoff score of 5 indicates substantial fatigue [31].

Goal Attainment Scaling (GAS) is an individualised evaluative instrument used to measure changes in patients based on self-selected goals. It uses a 5-point Likert scale to rate goal achievement, ranging from much worse to much better than expected [32]. ). It has been found applicable in patients with acquired brain lesions [33] and is considered reliable in pediatric rehabilitation [34].

**References**

1. Wade DT. Measurement in neurological rehabilitation. Oxford: Oxford Medical Publications; 1992.

2. Mehrholz J, Wagner K, Rutte K, Meissner D, Pohl M. Predictive validity and responsiveness of the functional ambulation category in hemiparetic patients after stroke. Arch Phys Med Rehabil. 2007;88(10):1314-9. Epub 2007/10/03. doi: 10.1016/j.apmr.2007.06.764. PubMed PMID: 17908575.

3. Klotz SGR, Petersen-Ewert C, Ketels G, Scherer M, Barzel A. The German version of the Functional Walking Categories (FWC): translation and initial validation. Top Stroke Rehabil. 2019;26(1):49-57. Epub 2018/10/23. doi: 10.1080/10749357.2018.1536022. PubMed PMID: 30346912.

4. Bohannon RW. Comfortable and maximum walking speed of adults aged 20-79 years: reference values and determinants. Age Ageing. 1997;26(1):15-9. PubMed PMID: 9143432.

5. Wade DT, Wood VA, Heller A, Maggs J, Langton Hewer R. Walking after stroke. Measurement and recovery over the first 3 months. Scand J Rehabil Med. 1987;19(1):25-30. Epub 1987/01/01. PubMed PMID: 3576138.

6. Rossier P, Wade DT. Validity and reliability comparison of 4 mobility measures in patients presenting with neurologic impairment. Arch Phys Med Rehabil. 2001;82(1):9-13. doi: 10.1053/apmr.2001.9396. PubMed PMID: 11239279.

7. van Hedel HJ, Wirz M, Curt A. Improving walking assessment in subjects with an incomplete spinal cord injury: responsiveness. Spinal cord. 2006;44(6):352-6. Epub 2005/11/24. doi: 10.1038/sj.sc.3101853. PubMed PMID: 16304565.

8. van Hedel HJ, Wirz M, Dietz V. Assessing walking ability in subjects with spinal cord injury: validity and reliability of 3 walking tests. Arch Phys Med Rehabil. 2005;86(2):190-6. Epub 2005/02/12. doi: 10.1016/j.apmr.2004.02.010. PubMed PMID: 15706542.

9. van Hedel HJ. Gait speed in relation to categories of functional ambulation after spinal cord injury. Neurorehabil Neural Repair. 2009;23(4):343-50. Epub 2008/11/28. doi: 10.1177/1545968308324224. PubMed PMID: 19036717.

10. Salbach NM, Mayo NE, Higgins J, Ahmed S, Finch LE, Richards CL. Responsiveness and predictability of gait speed and other disability measures in acute stroke. Arch Phys Med Rehabil. 2001;82(9):1204-12. Epub 2001/09/12. doi: 10.1053/apmr.2001.24907. PubMed PMID: 11552192.

11. Watson MJ. Refining the ten-metre walking test for use with neurologically impaired people. Physiotherapy. 2002;88(7):386-97.

12. Wrisley DM, Marchetti GF, Kuharsky DK, Whitney SL. Reliability, internal consistency, and validity of data obtained with the functional gait assessment. Phys Ther. 2004;84(10):906-18. Epub 2004/09/29. PubMed PMID: 15449976.

13. A. T. S. Committee on Proficiency Standards for Clinical Pulmonary Function Laboratories. ATS statement: guidelines for the six-minute walk test. American journal of respiratory and critical care medicine. 2002;166(1):111-7. doi: 10.1164/ajrccm.166.1.at1102. PubMed PMID: 12091180.

14. Potter K, Cohen ET, Allen DD, Bennett SE, Brandfass KG, Widener GL, et al. Outcome measures for individuals with multiple sclerosis: recommendations from the American Physical Therapy Association neurology section task force. Physical Therapy. 2014;94(5):593-608. doi: 10.2522/ptj.20130149. PubMed PMID: 24363338.

15. Podsiadlo D, Richardson S. The timed "Up & Go": a test of basic functional mobility for frail elderly persons. J Am Geriatr Soc. 1991;39(2):142-8. PubMed PMID: 1991946.

16. Dite W, Temple VA. A clinical test of stepping and change of direction to identify multiple falling older adults. Arch Phys Med Rehabil. 2002;83(11):1566-71. PubMed PMID: 12422327.

17. Franchignoni FP, Tesio L, Ricupero C, Martino MT. Trunk control test as an early predictor of stroke rehabilitation outcome. Stroke. 1997;28(7):1382-5. Epub 1997/07/01. doi: 10.1161/01.str.28.7.1382. PubMed PMID: 9227687.

18. Fischer N. Trunk Control Test – Rumpfkontrolle erfassen. ergopraxis. 2014;7(06):32-3.

19. Hsieh CL, Sheu CF, Hsueh IP, Wang CH. Trunk control as an early predictor of comprehensive activities of daily living function in stroke patients. Stroke. 2002;33(11):2626-30. Epub 2002/11/02. doi: 10.1161/01.str.0000033930.05931.93. PubMed PMID: 12411652.

20. Duncan PW, Weiner DK, Chandler J, Studenski S. Functional reach: a new clinical measure of balance. J Gerontol. 1990;45(6):M192-7. Epub 1990/11/01. doi: 10.1093/geronj/45.6.m192. PubMed PMID: 2229941.

21. Persson CU, Sunnerhagen KS, Lundgren-Nilsson A. Rasch analysis of the modified version of the postural assessment scale for stroke patients: postural stroke study in Gothenburg (POSTGOT). BMC Neurol. 2014;14:134. Epub 2014/06/21. doi: 10.1186/1471-2377-14-134. PubMed PMID: 24946807; PubMed Central PMCID: PMCPMC4069346.

22. Katz-Leurer M, Fisher I, Neeb M, Schwartz I, Carmeli E. Reliability and validity of the modified functional reach test at the sub-acute stage post-stroke. Disabil Rehabil. 2009;31(3):243-8. Epub 2008/07/09. doi: 10.1080/09638280801927830. PubMed PMID: 18608433.

23. Tinetti ME, Richman D, Powell L. Falls Efficacy as a Measure of Fear of Falling. Journal of gerontology. 1990;45(6):P239-P43. doi: 10.1093/geronj/45.6.P239.

24. Yardley L, Beyer N, Hauer K, Kempen G, Piot-Ziegler C, Todd C. Development and initial validation of the Falls Efficacy Scale-International (FES-I). Age Ageing. 2005;34(6):614-9. Epub 2005/11/04. doi: 10.1093/ageing/afi196. PubMed PMID: 16267188.

25. Dias N, Kempen GIJM, Todd CJ, Beyer N, Freiberger E, Piot-Ziegler C, et al. Die Deutsche Version der Falls Efficacy Scale-International Version (FES-I). Zeitschrift fur Gerontologie und Geriatrie. 2006;39(4):297-300. doi: 10.1007/s00391-006-0400-8.

26. Herdman M, Gudex C, Lloyd A, Janssen M, Kind P, Parkin D, et al. Development and preliminary testing of the new five-level version of EQ-5D (EQ-5D-5L). Quality of life research : an international journal of quality of life aspects of treatment, care and rehabilitation. 2011;20(10):1727-36. doi: 10.1007/s11136-011-9903-x. PubMed PMID: 21479777; PubMed Central PMCID: PMC3220807.

27. Ludwig K, Graf von der Schulenburg JM, Greiner W. German Value Set for the EQ-5D-5L. PharmacoEconomics. 2018;36(6):663-74. Epub 2018/02/21. doi: 10.1007/s40273-018-0615-8. PubMed PMID: 29460066; PubMed Central PMCID: PMCPMC5954069 EuroQol Research Foundation (the copyright holders of EQ-5D-5L). J.-Matthias Graf von der Schulenburg has no conflicts of interest directly relevant to the content of this article. ETHICS APPROVAL: The study received ethical approval from the Ethics Committee of Hannover Medical School (1598–2012) and was conducted in accordance with the Declaration of Helsinki. CONSENT TO PARTICIPATE: Informed consent was obtained from all individual participants included in the study. Participants were informed about their freedom of refusal. Anonymity and confidentiality were maintained throughout the research process. DATA AVAILABILITY STATEMENT: The datasets generated during and/or analyzed during the current study are available from the corresponding author on reasonable request.

28. Ludwig K, von der Schulenburg JG, Greiner W. Valuation of the EQ-5D-5L with composite time trade-off for the German population - an exploratory study. Health Qual Life Outcomes. 2017;15(1):39. Epub 2017/02/22. doi: 10.1186/s12955-017-0617-9. PubMed PMID: 28219389; PubMed Central PMCID: PMCPMC5319015.

29. Krupp LB, LaRocca NG, Muir-Nash J, Steinberg AD. The Fatigue Severity Scale: Application to Patients With Multiple Sclerosis and Systemic Lupus Erythematosus. Archives of neurology. 1989;46(10):1121-3. doi: 10.1001/archneur.1989.00520460115022.

30. Valko PO, Bassetti CL, Bloch KE, Held U, Baumann CR. Validation of the fatigue severity scale in a Swiss cohort. Sleep. 2008;31(11):1601-7. Epub 2008/11/19. doi: 10.1093/sleep/31.11.1601. PubMed PMID: 19014080; PubMed Central PMCID: PMCPMC2579971.

31. Pfeffer A. Einsatz bei Erschöpfung. physiopraxis. 2008;6(10):42-3.

32. Kiresuk TJ, Sherman RE. Goal attainment scaling: A general method for evaluating comprehensive community mental health programs. Community Ment Health J. 1968;4(6):443-53. Epub 1968/12/01. doi: 10.1007/bf01530764. PubMed PMID: 24185570.

33. Bouwens SF, van Heugten CM, Verhey FR. The practical use of goal attainment scaling for people with acquired brain injury who receive cognitive rehabilitation. Clin Rehabil. 2009;23(4):310-20. Epub 2009/01/31. doi: 10.1177/0269215508101744. PubMed PMID: 19179354.

34. Hurn J, Kneebone I, Cropley M. Goal setting as an outcome measure: A systematic review. Clin Rehabil. 2006;20(9):756-72. Epub 2006/09/29. doi: 10.1177/0269215506070793. PubMed PMID: 17005500.
