# Supplementary material for: Robot-assisted gait training in patients with various neurological diseases: A mixed methods feasibility study
Source: PLoS One. 2024 Aug 27;19(8):e0307434. doi: 10.1371/journal.pone.0307434 (PMC11349200; doi:10.1371/journal.pone.0307434)
Supplement: S1 Table — (DOCX) [file pone.0307434.s007.docx]

**S1 Table. Good Reporting of A Mixed Methods Study (GRAMMS) checklist.**

| Guideline | Section: page |
| --- | --- |
| Describe the justification for using a mixed methods approach to the research question | Methods- study design p. 5 |
| Describe the design in terms of the purpose, priority and sequence of methods | Methods- study design p. 5 |
| Describe each method in terms of sampling, data collection and analysis | Study flow Fig 1  Study population pp. 5-6  Outcome measures pp. 7-10  Qualitative data collection p. 9  Data analyses pp. 10-13 |
| Describe where integration has occurred, how it has occurred and who has participated in it | Results – integration of findings: p. 19; Fig 6 |
| Describe any limitation of one method associated with the present of the other method | Discussion – strengths and limitations: pp. 25-26 |
| Describe any insights gained from mixing or integrating methods | Discussion and Conclusions: pp. 20-26 |

O'Cathain A, Murphy E, Nicholl J. The quality of mixed methods studies in health services research. J Health Serv Res Policy. 2008;13: 92-98
