# Supplementary material for: Robot-assisted gait training in patients with various neurological diseases: A mixed methods feasibility study
Source: PLoS One. 2024 Aug 27;19(8):e0307434. doi: 10.1371/journal.pone.0307434 (PMC11349200; doi:10.1371/journal.pone.0307434)
Supplement: S2 Table — (DOCX) [file pone.0307434.s008.docx]

## S2 Table. Semi-structured observation protocol.

Date: **…………….**

| **Semi-structured observation** | |
| --- | --- |
| **Observation parameter** | **Notes** |
| Patient set-up |  |
| Patient body position referring to trunk, upper and lower extremities |  |
| % body-weight support |  |
| Patient effort |  |
| Tiredness of patient |  |
| Patient's expressions of discomfort or pain |  |
| Patient's facial expression |  |
| Patient's face colour |  |
| Patient's verbal expressions |  |
| Patient's non-verbal expressions |  |
| Therapist's verbal expressions |  |
| Therapist's non-verbal expressions |  |
| Patient's motivation |  |
| Communication between therapist and patient |  |
| Atmosphere in the room |  |
| Spatial environment |  |
| Progress regarding the intervention |  |
| Patient closure |  |
| **Unstructured observations** | |
|  | |
|  | |
|  | |
|  | |
