# Supplementary material for: Robot-assisted gait training in patients with various neurological diseases: A mixed methods feasibility study
Source: PLoS One. 2024 Aug 27;19(8):e0307434. doi: 10.1371/journal.pone.0307434 (PMC11349200; doi:10.1371/journal.pone.0307434)
Supplement: S3 Table — (DOCX) [file pone.0307434.s009.docx]

**S3 Table. Reflexivity of the researchers carrying out the thematic analysis.**

| **Author** | **Characteristics and qualifications** |
| --- | --- |
| Barbara Seebacher | Female scientist with a PhD degree in physiotherapy including graduate-level training in quantitative, qualitative, and mixed research methods, extensive experience with conducting and teaching qualitative, qualitative and mixed method research at master’s level, and clinical training and advanced practice in neurology, pediatrics, and musculoskeletal rehabilitation |
| Isabella Hotz | Female research associate with a Master’s degree in Therapeutic Sciences, f4 and graduate-level training in qualitative and mixed research methods, and clinical training and practice as a neurological rehabilitation physiotherapist |
| Bianca Slamik | Female physiotherapy-scientist studying for a Master’s degree in Neurorehabilitation, with the present research representing a partial fulfilment of her Master’s degree; f4 and graduate-level training in qualitative research methods, and clinical training and practice as a neurological physiotherapist |
| Sarah Mildner | Female research associate with a Master’s degree in Applied Health Sciences, f4 and graduate-level training in digital technologies, health-related innovations, interdisciplinary team management, and research methods, and clinical training and practice as an orthopaedic and neurological rehabilitation physiotherapist |
