# Supplementary material for: Robot-assisted gait training in patients with various neurological diseases: A mixed methods feasibility study
Source: PLoS One. 2024 Aug 27;19(8):e0307434. doi: 10.1371/journal.pone.0307434 (PMC11349200; doi:10.1371/journal.pone.0307434)
Supplement: S4 Table — (DOCX) [file pone.0307434.s010.docx]

**S4 Table. Results of secondary outcomes.**

| Parameter | Stroke NA (n=5) | Stroke A (n=5) | MS (n=5) | PD (n=3) | MND (n=2) | SCI (n=2) | SCA (n=2) | PNP (n=2) | All (n=26) | Effect size r |
| --- | --- | --- | --- | --- | --- | --- | --- | --- | --- | --- |
| BL 10MWT m/s*^1^ | 0.51 (0.49 to 0.52) | 0.85 (0.32 to 1.09) | 0.65 (0.24 to 1.36) | 1.36 (1.28 to 1.40) | 0.71 (0.66 to 0.75) | 0.80 (0.20 to 1.40) | 0.99 (0.79 to 1.18) | 1.72 (1.31 to 2.12) | 0.83 (0.20 to 2.12) |  |
| PI 10MWT m/s*^1^ | 0.79 (0.79 to 0.79) | 1.03 (0.37 to 1.20) | 0.94 (0.44 to 1.57) | 1.98 (1.46 to 2.29) | 0.97 (0.69 to 1.24) | 1.05 (0.36 to 1.73) | 1.24 (1.12 to 1.35) | 2.24 (1.91 to 2.56) | 1.09 (0.36 to 2.59) |  |
| Diff 10MTW m/s*^1^ | 0.27 (0.27 to 0.27) | 0.09 (0.05 to 0.18) | 0.20 (0.05 to 0.29) | 0.62 (0.18 to 0.89) | 0.26 (0.03 to 0.49) | 0.25 (0.16 to 0.33) | 0.25 (0.17 to 0.33) | 0.52 (0.44 to 0.60) | 0.19 (0.03 to 0.89) | 0.876 |
| BL 6 MWT m*^1^ | - | 280.00 (132.00 to 324.00) | 235.31 (147.00 to 334.00) | 379.00 (328.00 to 515.00) | 207.00 (170.00 to 244.00) | 233.95 (40.00 to 427.90) | 276.00 (180.00 to 372.00) | 437.50 (400.00 to 475.00) | 285.00 (40.00 to 515.00) |  |
| PI 6 MWT m*^1^ | 200.00 (200.00 to 200.00) | 294.00 (143.00 to 358.00) | 350.00 (75.00 to 417.00) | 529.00 (365.00 to 595.00) | 228.00 (210.00 to 246.00) | 269.00 (92.00 to 446.00) | 361.00 (307.00 to 415.00) | 541.50 (508.00 to 575.00) | 353.50 (75.00 to 595.00) |  |
| Diff 6MTW m*^1^ | - | 14.00 (7.00 to 67.00) | 87.69 (33.00 to 145.60) | 80.00 (37.00 to 150.00) | 21.00 (2.00 to 40.00) | 35.05 (18.10 to 52.00) | 85.00 (43.00 to 127.00) | 104.00 (33.00 to 175.00) | 41.5 (2.00 to 175.00) | 0.877 |
| BL TUG s**^1^ | 90.00 (36.03 to 90.00) | 15.76 (14.60 to 36.65) | 16.26 (7.05 to 50.07) | 7.87 (6.32 to 10.78) | 27.21 (17.41 to 37.00) | 25.40 (9.62 to 41.18) | 15.52 (11.35 to 19.68) | 8.93 (7.75 to 10.10) | 16.01 (6.32 to 90.00) |  |
| PI TUG s**^1^ | 24.87 (17.23 to 32.50) | 13.34 (9.95 to 33.88) | 14.30 (6.31 to 22.44) | 6.17 (3.96 to 9.53) | 20.17 (14.78 to 25.56) | 14.52 (7.92 to 21.11) | 13.32 (9.90 to 16.73) | 6.88 (6.72 to 7.03) | 13.34 (3.96 to 33.88) |  |
| Diff TUG s**^1^ | -38.15 (-57.50 to -18.80) | -2.77 (-9.23 to -1.31) | -2.25 (-27.63 to -0.74) | -1.70 (-2.36 to -1.25) | -7.04 (-11.44 to -2.63) | -10.88 (-20.07 to -1.70) | -2.20 (-2.95 to -1.45) | -2.05 (-3.38 to -0.72) | -2.63 (-0.72 to -57.50) | 0.875 |
| BL FSST s**^1^ | - | 12.16 (8.90 to 49.09) | 11.16 (7.38 to 25.25) | 8.50 (5.18 to 12.38) | 21.00 (14.53 to 27.47) | 31.59 (8.91 to 54.26) | 30.16 (30.16 to 30.16) | 9.98 (9.82 to 10.13) | 12.16 (5.18 to 54.26) |  |
| PI FSST s**^1^ | 55.29 (55.29 to 55.29) | 9.72 (8.14 to 45.01) | 13.06 (7.53 to 27.53) | 8.06 (4.80 to 9.00) | 18.62 (11.37 to 25.87) | 18.59 (7.88 to 29.29) | 14.39 (13.22 to 15.56) | 7.85 (6.66 to 9.03) | 10.53 (4.80 to 55.29) |  |
| Diff FSST s**^1^ | - | -2.95 (-4.36 to -0.76) | -0.17 (-4.72 to -0.15) | -0.44 (-3.38 to -0.38) | -2.38 (-3.16 to -1.60) | -13.00 (-24.97 to -1.03) | -16.94 (-16.94 to -16.94) | -2.13 (-3.47 to -0.79) | -1.60 (-24.97 to 0.15) | 0.868 |
| BL Fallers**^2^ | 2.00 (7.70%) | 1.00 (3.80%) | 0.00 (0.00%) | 0.00 (0.00%) | 1.00 (3.80%) | 1.00 (3.80%) | 0.00 (0.00%) | 0.00 (0.00%) | 5.00 (19.20%) |  |
| BL Non-fallers**^2^ | 3.00 (11.50%) | 4.00 (15.40%) | 5.00 (19.20%) | 3.00 (11.50%) | 1.00 (3.80%) | 1.00 (3.80%) | 2.00 (7.70%) | 2.00 (7.70%) | 21.00 (80.80%) |  |
| PI Fallers**^2^ | 0.00 (0.00%) | 0.00 (0.00%) | 0.00 (0.00%) | 0.00 (0.00%) | 0.00 (0.00%) | 1.00 (3.80%) | 0.00 (0.00%) | 0.00 (0.00%) | 1.00 (3.80%) |  |
| PI Non-fallers**^2^ | 5.00 (19.20%) | 5.00 (19.20%) | 5.00 (19.20%) | 3.00 (11.50%) | 2.00 (7.70%) | 1.00 (3.60%) | 2.00 (7.70%) | 2.00 (7.70%) | 25.00 (96.20%) |  |
| BL FAC*^1^ | 0.00 (0.00 to 1.00) | 4.00 (3.00 to 4.00) | 4.00 (2.00 to 4.00) | 4.00 (4.00 to 4.00) | 4.00 (3.00 to 4.00) | 4.00 (4.00 to 4.00) | 4.00 (4.00 to 4.00) | 4.00 (4.00 to 4.00) | 4.00 (0.00 to 4.00) |  |
| PI FAC*^1^ | 2.00 (0.00 to 3.00) | 4.00 (4.00 to 5.00) | 4.00 (4.00 to 5.00) | 5.00 (5.00 to 5.00) | 4.00 (4.00 to 4.00) | 5.00 (4.00 to 5.00) | 5.00 (4.00 to 5.00) | 5.00 (5.00 to 5.00) | 4.00 (0.00 to 5.00) |  |
| Diff FAC*^1^ | 1.00 (0.00 to 3.00) | 0.00 (0.00 to 2.00) | 1.00 (0.00 to 2.00) | 1.00 (1.00 to 1.00) | 0.50 (0.00 to 1.00) | 0.50 (0.00 to 1.00) | 0.50 (0.00 to 1.00) | 1.00 (1.00 to 1.00) | 1.00 (0.00 to 3.00) | 0.726 |
| BL FGA*^1^ | - | 9.00 (5.00 to 14.00) | 10.00 (5.00 to 21.00) | 22.00 (10.00 to 26.00) | 7.50 (7.00 to 8.00) | 12.00 (7.00 to 17.00) | 6.50 (4.00 to 9.00) | 13.00 (12.00 to 14.00) | 9.50 (4.00 to 26.00) |  |
| PI FGA*^1^ | - | 16.00 (8.00 to 16.00) | 14.50 (7.00 to 25.00) | 27.00 (18.00 to 29.00) | 9.50 (7.00 to 12.00) | 18.00 (11.00 to 25.00) | 13.00 (12.00 to 14.00) | 22.00 (20.00 to 24.00) | 15.50 (7.00 to 29.00) |  |
| Diff FGA*^1^ | - | 3.00 (2.00 to 10.00) | 3.00 (1.00 to 8.00) | 7.00 (1.00 to 8.00) | 2.00 (0.00 to 4.00) | 6.00 (4.00 to 6.00) | 6.50 (4.00 to 9.00) | 9.00 (8.00 to 10.00) | 4.00 (0.00 to 10.00) | 0.857 |
| BL FES-I**^1^ | 52.00 (33.00 to 58.00) | 21.00 (16.00 to 43.00) | 11.16 (7.38 to 25.53) | 21.00 (17.00 to 34.00) | 34.00 (27.00 to 41.00) | 38.50 (33.00 to 44.00) | 35.50 (31.00 to 40.00) | 21.00 (21.00 to 21.00) | 33.00 (16.00 to 58.00) |  |
| PI FES-I**^1^ | 48.00 (24.00 to 51.00) | 17.00 (16.00 to 27) | 13.06 (7.53 to 27.53) | 20.00 (16.00 to 28.00) | 31.00 (26.00 to 36.00) | 34.50 (30.00 to 39.00) | 30.50 (25.00 to 36.00) | 18.00 (18.00 to 18.00) | 26.50 (16.00 to 51.00) |  |
| Diff FES-I**^1^ | -9.00 (-11.00 to -2.00) | -2.00 (-16.00 to 0.00) | -10.00 (-24.00 to 0.00) | -1.00 (-6.00 to -1.00) | -3.00 (-5.00 to -1.00) | -4.00 (-5.00 to -3.00) | -5.00 (-15.00 to 5.00) | -3.00 (-3.00 to -3.00) | -4.00 (-24.00 to 5.00) | 0.772 |
| BL FSS**^1^ | 40.00 (13.00 to 56.00) | 33.00 (9.00 to 54.00) | 54.00 (25.00 to 63.00) | 21.00 (16.00 to 37.00) | 26.00 (24.00 to 28.00) | 56.00 (30.00 to 40.00) | 49.50 (44.00 to 55.00) | 26.00 (20.00 to 32.00) | 38.50 (9.00 to 63.00) |  |
| PI FSS**^1^ | 36.00 (23.00 to 50.00) | 25.00 (9.00 to 34.00) | 37.00 (9.00 to 63.00) | 20.00 (13.00 to 22.00) | 27.50 (17.00 to 38.00) | 35.00 (30.00 to 40.00) | 29.00 (28.00 to 30.00) | 27.5 (13.00 to 42.00) | 29.00 (9.00 to 63.00) |  |
| Diff FSS**^1^ | -2.00 (-20.00 to 10.00) | 0.00 (-29.00 to 1.00) | -11.00 (-18.00 to 1.00) | -8.00 (-17.00 to 6.00) | 1.50 (-7.00 to 10.00) | -21.00 (-25.00 to -17.00) | -20.50 (-25.00 to -16.00) | 1.50 (-7.00 to 10.00) | -7.50 (-29.00 to 10.00) | 0.544 |
| BL BDI-II**^1^ | 11.00 (5.00 to 31) | 8.00 (2.00 to 22.00) | 13.00 (0.00 to 35.00) | 8.00 (7.00 to 11.00) | 10.00 (3.00 to 17.00) | 19.00 (10.00 to 28.00) | 10.50 (10.00 to 11.00) | 13.00 (5.00 to 21.00) | 10.50 (0.00 to 35.00) |  |
| PI BDI-II**^1^ | 6.00 (3.00 to 16.00) | 4.00 (2.00 to 9.00) | 5.00 (0.00 to 26.00) | 9.00 (1.00 to 11.00) | 5.50 (0.00 to 11.00) | 11.50 (2.00 to 21.00) | 4.50 (2.00 to 7.00) | 12.5 (8.00 to 17.00) | 6.50 (0.00 to 26.00) |  |
| Diff BDI-II**^1^ | -2.00 (-15.00 to -1.00) | -2.00 (-18.00 to 5.00) | -9.00 (-13.00 to 0.00) | 2.00 (-7.00 to 4.00) | -4.50 (-6.00 to -3.00) | -7.50 (-8.00 to -7.00) | -6.00 (-8.00 to -4.00) | -0.50 (-4.00 to 3.00) | -4.00 (-18.00 to 5.00) | 0.647 |
| BL EQ5D5L VAS*^1^ | 40.00 (30.00 to 70.00) | 75.00 (37.00 to 98.00) | 50.00 (20.00 to 75.00) | 70.00 (43.00 to 70.00) | 82.50 (80.00 to 85.00) | 53.50 (35.00 to 72.00) | 75.00 (70.00 to 80.00) | 77.50 (65.00 to 90.00) | 70.00 (20.00 to 98.00) |  |
| PI EQ5D5L VAS*^1^ | 40.00 (25.00 to 80.00) | 90.00 (66.00 to 95.00) | 80.00 (30.00 to 100.00) | 74.00 (70.00 to 88.00) | 92.50 (90.00 to 95.00) | 72.50 (65.00 to 80.00) | 70.00 (70.00 to 70.00) | 81.50 (68.00 to 95.00) | 77.00 (25.00 to 100.00) |  |
| Diff EQ5D5L VAS*^1^ | 0.00 (-27.00 to 20.00) | 15.00 (-3.00 to 41.00) | 20.00 (10.00 to 45.00) | 13.00 (00.00 to 31.00) | 10.00 (5.00 to 15.00) | 19.00 (8.00 to 30.00) | -5.00 (-10.00 to 0.00) | 4.00 (3.00 to 5.00) | 10.00 (-27.00 to 45.00) | 0.579 |
| BL EQ5D5L Index*^1^ | 0.29 (-0.10 to 0.80) | 0.93 (0.62 to 1.00) | 0.77 (0.09 to 1.00) | 0.97 (0.75 to 0.97) | 0.82 (0.78 to 0.86) | 0.77 (0.67 to 0.87) | 0.85 (0.69 to 1.00) | 0.83 (0.79 to 0.87) | 0.78 (-0.10 to 1.00) |  |
| PI EQ5D5L Index*^1^ | 0.31 (0.28 to 0.94) | 0.92 (0.82 to 0.94) | 0.84 (0.11 to 1.00) | 0.88 (0.88 to 0.89) | 0.96 (0.92 to 1.00) | 0.83 (0.73 to 0.94) | 0.90 (0.82 to 0.97) | 0.90 (0.89 to 0.92) | 0.88 (0.11 to 1.00) |  |
| Diff EQ5D5L Index*^1^ | 0.11 (-0.25 to 0.39) | 0.02 (-0.06 to 0.30) | 0.02 (-0.05 to 0.08) | -0.09 (-0.10 to 0.14) | 0.14 (0.14 to 0.15) | 0.07 (0.06 to 0.07) | 0.05 (-0.18 to 0.28) | 0.07 (0.05 to 0.10) | 0.07 (-0.25 to 0.39) | 0.404 |

10MWT, 10-Metre Walk Test; 6MWT, 6-Minute Walk Test; A, ambulatory patients; BDI-II, Beck Depression Inventory second edition; BL, Baseline; EQ-5D-5L, 5-level EQ-5D version; FAC, Functional Ambulation Categories; FES-I, Falls Efficacy Scale-International; FGA, Functional Gait Assessment; FSS, Fatigue Severity Scale; FSST, Four Square Step Test; m, metres; m/s, metre per second; MND, motor neuron disease; MS, multiple sclerosis; N, number; NA, non-ambulatory; PD, Parkinson’s disease; PI, post-intervention; PNP, acute or chronic inflammatory demyelinating polyneuropathy; r, correlation coefficient based on Wilcoxon signed rank test; s, seconds; SCA, spinocerebellar ataxia; SCI, spinal cord injury (spastic para- or tetraplegia); TUG, Timed Up and Go.
^1^Values represent median (minimum to maximum).
^2^Frequency (percentage).
*Higher scores represent improvement.
**Higher scores represent worsening.
