# Supplementary material for: Robot-assisted gait training in patients with various neurological diseases: A mixed methods feasibility study
Source: PLoS One. 2024 Aug 27;19(8):e0307434. doi: 10.1371/journal.pone.0307434 (PMC11349200; doi:10.1371/journal.pone.0307434)
Supplement: S5 Table — (DOCX) [file pone.0307434.s011.docx]

**S5 Table. Results of gait parameters.**

| Parameter | Stroke NA (n=5) | Stroke A (n=5) | MS (n=5) | PD (n=3 | MND (n=2) | SCI (n=2) | SCA (n=2) | PNP (n=2) | All (n=26) | Effect size r |
| --- | --- | --- | --- | --- | --- | --- | --- | --- | --- | --- |
| BL gait distance m*^1^ | 377.75 (316.25 to 433.00) | 903.00 (394.75 to 1152.00) | 454.33 (304.00 to 1072.25) | 1584.50 (878.50 to 1789.75) | 419.13 (398.75 to 439.50) | 639.38 (157.50 to 1121.25) | 629.38 (417.50 to 841.25) | 1443.38 (1090.25 to 1796.50) | 446.92 (157.50 to 1796.50) |  |
| PI gait distance m*^2^ | 565.75 (457.67 to 1192.50) | 1092.50 (931.67 to 1708.25) | 921.50 (515.75 to 1458.50) | 1872.25 (1187.50 to 1982.50) | 1045.25 (797.50 to 1293.00) | 1299.75 (600.00 to 1999.50) | 969.25 (733.25 to 1205.25) | 1580.13 (1569.75 to 1590.50) | 1066.63 (457.67 to 1999.50) |  |
| Diff gait distance m*^3^ | 188.00 (69.75 to 759.50) | 499.00 (-59.50 to 805.25) | 362.00 (112.75 to 540.34) | 309.00 (82.50 to 398.00) | 626.13 (398.75 to 853.50) | 660.38 (442.50 to 878.25) | 339.88 (315.75 to 364.00) | 136.75 (-206.00 to 479.50) | 392.13 (-206.00 to 878.25) | 0.829 |
| BL number of steps*^1^ | 922.50 (645.25 to 1014.00) | 1913.00 (1070.25 to 2104.50) | 930.67 (738.25 to 1975.00) | 3013.25 (1885.00 to 3027.75) | 879.75 (839.00 to 920.50) | 1349.88 (473.25 to 2226.50) | 1354.88 (843.00 to 1866.75) | 2746.38 (2260.25 to 3232.50) | 1073.29 (473.25 to 3232.50) |  |
| PI number of steps *^2^ | 1255.75 (1012.00 to 2267.00) | 2150.67 (2018.75 to 3054.00) | 1852.33 (1284.50 to 2466.50) | 3268.25 (2344.00 to 3401.00) | 1922.50 (1604.50 to 2240.50) | 2510.50 (1612.25 to 3408.75) | 1892.75 (1394.25 to 2391.25) | 2851.38 (2839.50 to 2863.25) | 2150.46 (1012.00 to 3408.75) |  |
| Diff number of steps*^3^ | 394.50 (89.50 to 1253.00) | 1074.34 (-85.75 to 1140.50) | 491.50 (134.25 to 921.66) | 387.75 (240.50 to 459.00) | 1042.75 (765.50 to 1320.00) | 1160.63 (1139.00 to 1182.25) | 537.88 (524.50 to 551.25) | 105.00 (-3393.00 to 603.00) | 577.13 (-393.00 to 1320.00) | 0.834 |
| BL average step length cm*^1^ | 44.00 (38.67 to 49.25) | 46.50 (36.75 to 54.00) | 46.00 (39.75 to 53.75) | 53.25 (45.75 to 58.50) | 47.00 (46.75 to 47.25) | 41.63 (33.25 to 50.00) | 46.88 (44.50 to 49.25) | 51.38 (47.75 to 55.00) | 46.63 (33.25 to 58.50) |  |
| PI average step length cm*^2^ | 48.75 (42.00 to 52.75) | 53.25 (43.33 to 55.75) | 48.75 (40.00 to 59.00) | 56.75 (50.75 to 57.75) | 53.38 (49.50 to 57.25) | 47.50 (36.75 to 58.25) | 51.38 (50.25 to 52.50) | 55.13 (54.75 to 55.50) | 51.63 (36.75 to 59.00) |  |
| Diff average step length cm*^3^ | 3.00 (0.50 to 10.25) | 5.50 (0.00 to 11.50) | 2.75 (0.25 to 5.25) | 5.00 (-1.75 to 5.50) | 6.38 (2.75 to 10.00) | 5.88 (3.50 to 8.25) | 4.50 (3.25 to 5.75) | 3.75 (0.50 to 7.00) | 3.58 (-1.75 to 11.50) | 0.831 |
| BL RAGT time min*^1^ | 22.75 (15.32 to 23.49) | 30.98 (22.91 to 32.79) | 19.39 (18.45 to 32.60) | 36.60 (32.70 to 39.29) | 18.97 (15.52 to 22.42) | 31.23 (22.45 to 40.02) | 26.93 (17.32 to 36.53) | 39.02 (38.20 to 39.85) | 23.86 (15.32 to 40.02) |  |
| PI RAGT time min*^2^ | 29.57 (20.73 to 34.03) | 36.44 (30.76 to 40.93) | 31.10 (26.15 to 34.41) | 39.35 (36.28 to 41.88) | 30.70 (30.08 to 31.32) | 41.46 (38.74 to 44.18) | 32.14 (22.58 to 41.46) | 39.31 (36.34 to 42.28) | 33.92 (20.73 to 44.18) |  |
| Diff RAGT time min*^3^ | 9.05 (-2.76 to 18.72) | 7.50 (1.23 to 16.70) | 6.77 (1.80 to 14.13) | 3.57 (0.06 to 5.28) | 11.73 (8.90 to 14.56) | 10.23 (4.16 to 16.29) | 5.21 (5.16 to 5.26) | 0.29 (-3.52 to 4.09) | 5.27 (-3.52 to 18.72) | 0.809 |
| BL maximum gait speed m/s*^1^ | 1.28 (1.13 to 1.58) | 2.03 (1.20 to 2.95) | 1.70 (1.13 to 2.78) | 3.15 (1.85 to 3.48) | 1.54 (1.38 to 1.70) | 1.44 (0.60 to 2.28) | 1.59 (1.58 to 1.60) | 2.74 (2.00 to 3.48) | 1.65 (0.60 to 3.48) |  |
| PI maximum gait speed m/s*^2^ | 1.68 (1.23 to 2.28) | 3.00 (1.67 to 3.08) | 1.83 (1.40 to 3.15) | 3.15 (2.18 to 3.65) | 2.67 (1.73 to 3.60) | 2.47 (1.25 to 3.68) | 2.12 (2.08 to 2.15) | 2.89 (2.30 to 3.48) | 2.18 (1.23 to 3.68) |  |
| Diff maximum gait speed m/s*^3^ | 0.20 (-0.10 to 1.10) | 0.97 (-0.77 to 1.70) | 0.35 (0.13 to 0.64) | 0.33 (-0.33 to 0.50) | 1.13 (0.30 to 2.22) | 1.03 (0.65 to 1.40) | 0.52 (0.50 to 0.55) | 0.15 (0.00 to 0.30) | 0.39 (-0.77 to 2.22) | 0.702 |

A, ambulatory patients; BL, Baseline; cm, centimetres; m, metres; min, minutes; MND, motor neuron disease; m/s, metre per second; MS, multiple sclerosis; N, number; NA, non-ambulatory patients; PD, Parkinson’s disease; PI, post-intervention; PNP, acute or chronic inflammatory demyelinating polyneuropathy; r, correlation coefficient based on Wilcoxon signed rank test; RAGT, Robot-assisted gait training; SCA, spinocerebellar ataxia; SCI, spinal cord injury (spastic para- or tetraplegia).
^1^Values represent median (minimum to maximum) of the first 4 sessions.
^2^Values represent median (minimum to maximum) of the last 4 sessions.
^3^Values represent median (minimum to maximum).
*Higher scores represent improvement.
**Higher scores represent worsening.
