# Supplementary material for: Robot-assisted gait training in patients with various neurological diseases: A mixed methods feasibility study
Source: PLoS One. 2024 Aug 27;19(8):e0307434. doi: 10.1371/journal.pone.0307434 (PMC11349200; doi:10.1371/journal.pone.0307434)
Supplement: S6 Table — (DOCX) [file pone.0307434.s012.docx]

**S6 Table. Coding tree and quotations.**

| **Code** | **Subcodes** | | **Quotations** |
| --- | --- | --- | --- |
| **Motivation (n=81)** | Interest (8) | | "Patient is very motivated and interested in the device" (MND_ID25, paragraph 48) |
|  | Concentration/focus (20) | | "Very concentrated and focused, laughing and smiling during the intervention (PNP_ID23, paragraph 23) |
|  | External focus of attention (12) | | "Therapist uses external focus with smiley function of stance leg – weight shift to the left side." (MS_ID6, paragraph 37) |
|  | Showing motivation (23) | | "The patient seems very motivated, which is shown by his body posture and the facial expressions" (Stroke_ID8, paragraph 35) |
|  | Active physical participation (8) | | "The patient seems motivated, always active, straightens up and cooperates well; towards the end she is proud of setting a new record today" (MS_ID6, paragraph 49) |
|  | Patients’ long-term goals (10) | | "I would like to walk unsupported with just a cane" (Stroke_ID1, paragraph 28) |
| **Self-determination during therapy (n=84)** | Patients’ goals for the session (12) | | "I am a bit tired from the eleventh minute, but would like to do fourteen minutes" (MND_ID24, paragraph 17) |
|  | Patients’ self-determination (39) | | "As the patient approaches the final intervention phase, she requests a cadence of 70 " (SCI_ID11, paragraph 27) |
|  | Patients’ active participation during set-up and closure (33) | | "The patient moves to the LEXO® device using one foot to propel his wheelchair and gets into position to stand up." (Stroke_ID4, paragraph 9)  "The patient manages to pull the right foot out of the footrest and get out of the wheelchair by herself." (Stroke_ID26, paragraph 68) |
| **Acceptance of the RAGT (n=43)** | Effectiveness and usefulness (13) | | "The device alone, the LEXO®, gives me more strength. And now I am able to take nice, brisk steps with the rollator using my right foot" (Stroke_ID26, paragraph 34) |
|  | Well-being and comfort (11) | | "[The device] runs well, I am feeling comfortable during the gait training and appreciate the settings." (MS_ID12, paragraph 22) |
|  | Wanting to continue using the LEXO® (4) | | "I want to take home the LEXO® next week, I will organise a crane" (MS_ID14, paragraph 26). |
|  | Appreciation of the patient transfer system (8) | | "The patient expresses that he would like to leave the device “flying” [using the patient transfer system]"" (PD_ID15, paragraph 59) |
|  | Initial unfamiliarity (5) | | "The first time it was difficult for me to get used to the device, in terms of the movement type and intensity" (MS_ID14, paragraph 27) |
|  | Undecided about the benefits of RAGT (2) | | "It's hard to say because the walking distances at home are different. When my legs need to decide for themselves when to take a step, it's different. The device now decides for me when I have to take a step. At home I have to walk up an incline from the main road to my house, sometimes it works better, sometimes worse. I can't say yet if it has helped me.” (MS_ID18, paragraph 28) |
| **Emotions (n=55)** | Nervousness, uncertainty and curiosity at the first session (12) | | "The patient seems a bit nervous what to expect as it is the first session, but he is interested." (SCA_ID22, paragraph 6) |
|  | Dissatisfaction with one's own performance (14) | | "I am not happy with my performance today, the first session was the best, that's where I was able to accomplish the most" (Stroke_ID8, paragraphs 34-35) |
|  | Satisfaction with one's own performance (10) | | "I have reached my goals so far; I’m able to walk safely again except stairs where I still feel weak." (MND_ID25, paragraph 27) |
|  | Pleasure during the RAGT (11) | | "This is awesome, I'm the best! I like it, now I like myself again" (Stroke_ID13, paragraphs 23-24) |
|  | Fun during the RAGT (5) | | "Towards the end of the intervention: "That would be a laugh" in response to the request to bring the two beams [indicating weight distribution of the legs] to the same level" (MS_ID6, paragraph 29) |
|  | Feeling of security because of familiarity with RAGT (1) | | "I already know what's coming" (MS_ID6, paragraph 26) |
| **Therapeutic relationship (n=160)** | Transparent and clear information (28) | | "The therapist announces all further steps in advance, asks about the correct setting of the parameters and directs the patient not to lean forward too much" (SCI_ID11, paragraphs 31-33) |
|  | Trust between therapist and patient (16) | | "The patient seems very motivated, always jokes around and lets the therapist push him further" (Stroke_ID19, paragraph 54) |
|  | Discussing results together (14) | | "There was a brief conversation about the achieved gait distance and the percentage of weight distribution to the right/left" (PD_ID15, paragraph 60) |
|  | Negative Patient-therapist interaction (8) | | "The therapist says that she would like to give the patient an additional task. The patient replies: "I won't do that. That burdens me." The therapist replies: "I would be quite different from you; I would at least want to hear the task."" (Stroke_ID19, paragraph 48) |
|  | Empathetic therapeutic response (8) | | "After talking about his goal, the patient gets into telling stories about what he was able to do and that the stroke was a dramatic change in life for him. Followed by a longer conversation about his coping strategies with the stroke" (Stroke_ID19, paragraph 45) |
|  | Loose, funny, exuberant atmosphere in the room and small talk (49) | | "The communication between patient and therapist was open and friendly. It was a very approachable atmosphere with therapeutic instruction next to exuberant stories and laughter" (Stroke_ID19, paragraphs 55-59) |
|  | Approval and encouragement through therapist (20) | | "The therapist approves for knee control of the patient: "Now you've got it! Great, the two beams [indicating weight distribution of the legs] are at the same level."" (MS_ID6, paragraph 43) |
|  | Physical support to assist therapy (17) | | "The therapist asks patient to straighten up and therefore provides physical support on the upper back" (PNP_ID9, paragraph 29) |
| **Observed improvement (n=25)** |  | | "Today, there was no break needed and the training time was increased by 8 min. Furthermore, the distance walked was increased, while the body-weight support was decreased" (MS_ID6, paragraph 54) |
| **Organisational aspects and procedure (n=10)** | Time of the day for the RAGT (4) | | "Sure, I'd prefer RAGT in the morning." (MS_ID6, paragraph 65) |
|  | Session preparation by the therapist (6) | | "The therapist has the wheelchair set up and gives exact information about the further procedure" (Stroke_ID4, paragraph 58) |
| **Perception of body position (n=5)** |  | | "I have to straighten up again. Then I’ll get the beams back up again [visual feedback about the weight distribution of the legs]" (PNP_ID23, paragraph 32) |
| **Physical symptoms during RAGT (n=47)** | Fatigue (2) | | "The patient seems generally very tired during the RAGT today." (MS_ID3, paragraph 20) |
|  | Clonus (6) | | "After 14 minutes, appears a slight clonus in the left leg." (Stroke_ID8, paragraph 17) |
|  | Rigor (1) | | "I’m experiencing a cramping sensation [rigor] in my left leg, which subsides 2 minutes after the end of the RAGT" (PD_ID17, paragraph 20) |
|  | Spasticity (9) | | “The session has to be paused again at 15:12 due to spasticity in the left leg" (MS_ID14, paragraph 14 - 15) |
|  | Pain (8) | | "The patient complains about a slight pulling in the groin after 20 minutes." (PD_ID21, paragraph 25) |
|  | Sensory loss (6) | | "Now, after we stopped the session, I have a strange feeling in my feet. It feels like a slight buzzing in my feet." (MS_ID12, paragraph 42) |
|  | Sore muscles from previous therapies (6) | | "The patient mentions muscle soreness in the buttocks at the beginning, but it does not interfere with the training" (SCI_ID11, paragraph 16) |
|  | Muscle fatigue (9) | | "The patient is very tired after the RAGT, walking becomes difficult, and he shows an increased hyperextension of the knee." (Stroke_ID8, paragraph 59) |
| **Robotic device settings that influence the therapy (n=44)** | Pelvic support to assist upright walking (2) | | "The patient is slightly kyphotic; pelvic support (without tethering) makes it easier for her to stand upright" (MS_ID6, paragraph 12) |
|  | Handrail height (2) | | "Maximum elevation of handrail on the left side, can relieve the cramp in left arm" (Stroke_ID1, paragraph 85) |
|  | Pause position (1) | | "During a break the patient explains that the pause position of the LEXO ® is not ideal. He wishes for the feet to be parallel." (PD_ID20, paragraph 27) |
|  | Automatic breaking of the gait robot to protect spastic/ataxic patients or during performance of additional tasks (e.g., throwing a ball) (4) | | "After 11 minutes of RAGT, brief faltering due to ataxia can be observed" (PNP_ID23, paragraph 59). |
|  | Heels slipping out of the shoes (2) | | "The therapist asks the patient to wear different, tighter shoes because the patient always slips out of the shoe with the left heel." (Stroke_ID28, paragraph 34) |
| **Training intensity (n=115)** | No (sufficient) exertion (3) | | "The patient shows hardly any tiredness - can talk in a relaxed manner throughout the session" (PD_ID15, paragraph 21) |
|  | Exhaustion (15) | | "The patient expresses that the therapy was very exhausting yesterday" (Stroke_ID8, paragraph 27) |
|  | Tiredness (18) | | "The patient is very tired after the RAGT." (MS_ID6, paragraph 19) |
|  | Breaks (24) | | "The patient needs a break after 14, 17 and 20 minutes of therapy, he then wishes to end the therapy" (Stroke_ID8, paragraph 65). |
|  | Body-weight support (7) | | "After nine and a half minutes the patient shows first signs of muscle fatigue, therefore more support is given to reduce body weight." (MS_ID6, paragraph 17) |
|  | Support using the handrail (7) | | "The patient holds on to the handrail with both hands from the 25th minute onwards." (PD_ID15, paragraph 15) |
|  | Vegetative signs 0 (41) | |  |
|  |  | Intensified breathing (14) | "The patient breathes very hard during the break." (SCI_ID7, paragraph 15) |
|  |  | Sweat or beads of sweat (18) | "The patient has sweat on the forehead after 16 minutes" (SCA_ID10, paragraph 22) |
|  |  | Red complexion due to exertion (9) | "The face colour of the patient was initially rosy; during the RAGT it gets slightly reddish strained" (PNP_ID9, paragraph 20). |

ID, identification number; MND, motor neuron disease; MS, multiple sclerosis; N, number of coded observations; PD, Parkinson’s disease; PNP, acute or chronic inflammatory demyelinating polyneuropathy; SCA, spinocerebellar ataxia; SCI, spinal cord injury (spastic para- or tetraplegia).
