# Supplementary material for: Robot-assisted gait training in patients with various neurological diseases: A mixed methods feasibility study
Source: PLoS One. 2024 Aug 27;19(8):e0307434. doi: 10.1371/journal.pone.0307434 (PMC11349200; doi:10.1371/journal.pone.0307434)
Supplement: Attachment — Submitted filename: Response to Reviewers.docx [file pone.0307434.s013.docx]

PONE-D-23-31858R1

Robot-assisted gait training in patients with various neurological diseases: a mixed methods feasibility study

PLOS ONE

*Dear Academic Editor,*

*Dear Dr. de Sire,*

*Dear Reviewers,*

*We thank the Editor and Reviewers for their valuable comments and feedback and for giving us a chance to submit a revised manuscript. Please find below our point-by-point responses. The main manuscript and figure S1 caption have been amended accordingly, with any changes highlighted to allow the Editor and Reviewers to check the adaptations made.*

*We thank the Editor and Reviewers for their time and effort and hope we have sufficiently responded to the Editor’s and Reviewers’ requests.*

*With kind regards,*

*Barbara Seebacher, on behalf of all the authors*

**Comments to the Author**

Reviewer #1:

Dear Authors,
Thank you for answering to the previous reviewer’s comments. I think you analized a cutting-edge topic, in my opinion the paper is well written.

*Dear Reviewer #1,*

*Thank you very much for reviewing our manuscript and acknowledging its value.*

Reviewer #2:

The authors intended to explore the feasibility, acceptability, goal attainment, and preliminary effects of RAGT in patients with common and rare neurological diseases. They recruited 28 inpatients after stroke who will receive the planned intervention. Among 26 patients, RAGT was considered highly feasible. The manuscript was well prepared for the proof of the concept purpose. Only minor comments were listed below.

*Dear Reviewer #2,*

*Thank you for reviewing our manuscript, acknowledging its value, and for offering your suggestions to enhance the quality of our manuscript. Please find our point-by-point response as follows.*

1. The authors argued no sample size calculation is needed due to the feasibility analysis, which is fine. But also argued “to account for an anticipated attrition rate of 10%”, which somewhat has sample size in mind. Please clarify.

*Response:*

*We appreciate your attention to detail in identifying this inconsistency. Previously, we mentioned that 'Given the study design, methods, and objectives, no extra patients were incorporated to accommodate an expected attrition rate of 10%.' Recognising the lack of value in this statement, we have since deleted it.*

2. Line 253. Effect sizes were calculated as correlation coefficients. Please clarify which two variables’ correlation do you refer to and how to interpret in this content.

*Response:*

*Thank you for your valuable feedback. As mentioned, the rank-biserial correlation coefficient (r) is a commonly used effect size measure in the context of the Wilcoxon signed-rank test. This coefficient provides estimates regarding the strength and direction of association between paired samples. To clarify, the paired samples we are referring to in this study are the baseline and post-intervention measures of the respective functional outcomes.*

*In response to your suggestion, we have included the following revised statement in the manuscript: ‘Effect sizes were calculated using the rank-biserial correlation coefficient (r) obtained from the Wilcoxon signed-rank test. This coefficient offers insights into the strength and direction of association between paired samples, specifically the baseline and post-intervention measures.’*

3. In the abstract, authors specifically pointed out the count for different diseases but nothing related was discussed later. What will be the main point to talk about in the abstract?

*Response:*

*Thank you for bringing this to our attention. Taking your feedback into consideration, we have removed the count for the different diseases from the abstract.*

4. As the sample size is extremely small, whether the significant results are replicable is uncertain. So the tone of the results or conclusion should be softened and focus on the feasibility of the study.

*Response:*

*We sincerely value this feedback. Taking into account your suggestion, we have revised our conclusions, which now read as follows:*

*‘To our knowledge, there is a gap in the literature regarding the exploration of quantitative RAGT effects alongside patient acceptance and satisfaction using qualitative methods across various neurological conditions. Our study aims to fill this gap by offering valuable insights into understanding patients' needs and identifying necessary adjustments to ensure that RAGT is both enjoyable and maximally effective for patients.*

*The outcomes of our study imply the feasibility of utilising the LEXO® gait trainer for RAGT among patients with various neurological conditions. This is evidenced by robust recruitment, retention, and adherence rates. Furthermore, the findings underscore the safety of this approach, as indicated by the occurrence of anticipated mild and transient adverse events, alongside its initial effectiveness in enhancing RAGT outcomes among patients with diverse neurological conditions. Moreover, the majority of patients experienced the training positively and expressed satisfaction with their performance and outcomes. While our findings are promising, it is essential to conduct subsequent adequately powered RCTs to validate our results regarding the safety and efficacy of end-effector-based RAGT in patients with MND and SCA. Additionally, further mixed-methods studies are warranted to provide more nuanced insights into patients' perceptions of RAGT with end-effector devices and the influence of the therapeutic relationship on RAGT outcomes.’*
